# *Stevia* Genus: Phytochemistry and Biological Activities Update

**DOI:** 10.3390/molecules26092733

**Published:** 2021-05-06

**Authors:** Jimena Borgo, Laura C. Laurella, Florencia Martini, Cesar A. N. Catalán, Valeria P. Sülsen

**Affiliations:** 1 Instituto de Química y Metabolismo del Fármaco (IQUIMEFA), CONICET—Universidad de Buenos Aires, Buenos Aires 1113, Argentina; jimeborgo@gmail.com (J.B.); lclposdoc@gmail.com (L.C.L.); flormartini1@gmail.com (F.M.); 2 Cátedra de Farmacognosia, Facultad de Farmacia y Bioquímica, Universidad de Buenos Aires, Buenos Aires 1113, Argentina; 3 Cátedra de Química Medicinal, Facultad de Farmacia y Bioquímica, Universidad de Buenos Aires, Buenos Aires 1113, Argentina; 4 Instituto de Química Orgánica, Facultad de Bioquímica Química y Farmacia, Universidad Nacional de Tucumán, Ayacucho 471 (T4000INI), San Miguel de Tucumán T4000, Argentina; ccatalan@fbqf.unt.edu.ar

**Keywords:** *Stevia*, sesquiterpene lactones, diterpenes, flavonoids, biological activity

## Abstract

The *Stevia* genus (Asteraceae) comprises around 230 species, distributed from the southern United States to the South American Andean region. *Stevia rebaudiana*, a Paraguayan herb that produces an intensely sweet diterpene glycoside called stevioside, is the most relevant member of this genus. Apart from *S. rebaudiana*, many other species belonging to the *Stevia* genus are considered medicinal and have been popularly used to treat different ailments. The members from this genus produce sesquiterpene lactones, diterpenes, longipinanes, and flavonoids as the main types of phytochemicals. Many pharmacological activities have been described for *Stevia* extracts and isolated compounds, antioxidant, antiparasitic, antiviral, anti-inflammatory, and antiproliferative activities being the most frequently mentioned. This review aims to present an update of the *Stevia* genus covering ethnobotanical aspects and traditional uses, phytochemistry, and biological activities of the extracts and isolated compounds.

## 1. Introduction

The genus *Stevia* represents one of the most diverse and characteristic of the tribe Eupatoriae, subfamily Asteroidae, family Asteraceae. Its distribution area ranges from the southern United States to the Andean region of South America, to northern Chile and northern Patagonia in Argentina [1,2]. Members of *Stevia* comprise herbs and shrubs that are found mainly 500–3500 m above sea level. They grow in a wide range of environments including grasslands, scrublands, forested mountain slopes, conifer forests, and subalpine vegetation, although they most often inhabit semi-dry mountainous terrains [3].

The number of species within the genus is estimated to be 230. Mexico and South America are characterized by highlands where *Stevia* species grow. Most species are found in South America, within Peru, Bolivia, southern Brazil, Paraguay, and northern Argentina, where approximately 120 species are found [3,4]. There are more than 80 species known to be in North America, and at least 70 are native to Mexico. Records show that the genus is not present in the Bahamas, the Antilles, or Amazonia [2,3].

The genus is known worldwide for the species *Stevia rebaudiana* (Bertoni) Bertoni (Asteraceae), popularly named “stevia”, which produces large amounts of stevioside, a powerful non-nutritive natural sweetener. Stevioside is composed of a mixture of several closely related *ent*-kaurene glycosides, among which stevioside, rebaudioside A, and dulcoside A and B are the most important. Stevioside is the most abundant sweetener that, together with the other diterpene glycosides, accumulates in the leaves. The aqueous extracts of *S. rebaudiana* are used commercially to sweeten different products and also for the extraction of sweet principles. *Stevia rebaudiana* has also demonstrated several biological activities in preclinical and clinical studies including antidiabetic, anticariogenic, antioxidant, antihypertensive, antimicrobial, anti-inflammatory, and antitumor activities, among others [5].

Revisions on the genus covering ethnobotany and phytochemistry data have been published previously [1,6,7]. However, in the last years, most of the scientific papers and reviews were dedicated almost exclusively to *S. rebaudiana* and its constituents. The aim of this review is to provide an update on the *Stevia* genus focusing on ethnobotanical, phytochemical, and pharmacological data published in the last decades. Articles covering the biological activities of extracts and isolated compounds of this genus will be analyzed and discussed.

## 2. Ethnobotany

Ethnobotanical data on *Stevia* species have been described since the 18th century. The latest revision covering ethnobotanical and ethnopharmacological aspects of the *Stevia* species was published in 2002 [6]. A literature survey covering the period January 2002–February 2021 was carried out here in order to update the information related to these topics.

The traditional uses of 29 species from Central and South America are available. Some of the most common popular uses of *Stevia* species are related to antidiarrheal, anti-inflammatory, antimalarial uses, as a febrifuge, a diuretic, a diaphoretic, to treat heart diseases, stomachaches, and skin conditions (Table 1).

Many plants such as *S. connata*, *S. eupatoria*, *S. puberula*, *S. serrata*, and *S. subpubescens* have been used for gastrointestinal disorders. Other species have been used as therapy for infectious diseases. In this sense, *S. bogotensis*, *S. eupatoria*, *S. glandulosa*, *S. pilosa*, and *S. salicifolia* have been employed as antipyrectic and antiparasitic. The anti-inflammatory use of *Stevia* plants has also been described in the literature; for instance, *S. eupatoria*, *S. lucida*, and *S. salicifolia* have been described as useful for the treatmet of inflammatory processes.

*Stevia eupatoria*, *S. lucida*, *S. salicifolia*, *S. serrata*, and *S. subpubescens* have been used in the United States, Mexico, and Central America and northern South America. These *Stevia* species have many associated medicinal properties and have been popularly used to treat a wide range of diseases.

*Stevia eupatoria*, known as “hierba del borrego”, “yerba del borrego”, and “cola del Borrego” has been described as antimalarial and has been used for its diuretic properties. Mexican folk medicine has used this species as an herbal remedy for stomach pain and for its hipoglycemic, analgesic, anti-inflammatory, and antihypertensive properties. This species is known as “estevia” (synonym of *S. purpurea*) [6,10,11].

*Stevia lucida* is widespread from Mexico to Venezuela. This plant is popularly known in Mexico as “yerba del aire” and “hierba de la araña”. In Guatemala, it is called “kebuj” and in Colombia, it is known as “chilca” and “golondrina de la sabanera”. In Venezuela, it is named “chilca” and “chirca”. *Stevia glutinosa*, a synonym of *S. lucida*, is called “javillo”, “javilla”, and “mariposa”. Several uses have been described for this species, including external use to relieve pain and treat wounds. In Colombia, a decoction of the aerial parts of *S. lucida* has been used to alleviate inflammatory processes. In Guatemala, it has been used to treat rheumatism. With the same purpose, in Maracaibo (Venezuela), a decoction of the leafy stems of *S. lucida* has been used. In Mexican folk medicine, “yerba del aire” has been used to treat chilly cramps [6,10].

*S. salicifolia* constitutes another species known with different common names and is distributed from southern United States to Mexico. In traditional medicine, it is known as “hierba del aire”, “hierba de la mula”, “zazal”, “zazale de olor, “yerba de la mula”, and “la envidia” in Mexico. This species, synonym of *S. stenophylla*, is also known as “hierba de la Santa Rita”. In Mercado Juarez (Toluca, Mexico), the dried aerial parts of *S. salicifolia* are marketed to prepare decoctions or alcoholic infusions that can be used “as a rub” to treat rheumatism. In Mexico, the decoction of the dried roots has been used as a cathartic, and the infusion of the roots has been recommended for intestinal upset due to parasites. The roots, mashed and placed in warm water, are used to prepare a drink employed as a purgative. The leaves are used to prepare tea (infusion) for colds and fevers [6,10].

*Stevia serrata* is known in traditional medicine as “ronino”, “uriki”, “otoninawa”, “chapo”, “yerba picante”, “hipericón”, “Q’ang’aj”, “anis silvestre”, and “hipericon arrie”. The external use of crushed roots of *S. serrata* for washes and poultices applied directly to open wounds has been reported. The whole plant crushed and rubbed has been employed in snake bites. This species has been described as a cough remedy and to treat gastrointestinal disorders [6,10,14].

In Mexico, *S. subpubescens* (synonym of *S. subpubescens* var. *subpubescens*) is commonly known as “zazal” and also as “hierba de la mula”. The aerial parts of this species have been recommended as a decoction to be used as a bath by women after parturition. The leaves are used for stomachaches and the whole fried plant can be rubbed on affected parts to treat joint pain [6,10].

Other species have also been described as medicinal. The decoction of *S. macbridei* has been used externally by women as a bath. The infusion or tea of *S. nepetifolia* has been suggested to alleviate dysmenorrhea symptoms. A drink prepared with the roots of *S. balansae* has been employed in Paraguay to treat diarrhea. The infusion of roots and flowers of *S. trifida* has been orally administrated to treat dysentery.

In the last years, several Argentinean *Stevia* species have been employed with an ornamental purpose. In this sense, *S. achalensis*, *S. fiebrigii* var. *vattuonei*, *S. mercedensis* var. *mercedensis*, *S. sanguinea*, *S. satureiifolia* var. *satureiifolia* and *S. yalae* can be mentioned. In particular, the species *S. fiebrigii* var. *vattuonei* and *S. mercedensis* var. *mercedensis* are commonly used in northern Argentina as ornamentation in religious festivities.

## 3. Phytochemistry

### 3.1. General Aspects

Being one of the largest and most easily recognizable from the tribe Eupatoriae, the genus *Stevia* is surprisingly diverse in its chemical composition. The phytochemistry of the *Stevia* genus was reviewed by Hernandez et al. [1] and by Cerda-García-Rojas and Pereda-Miranda [7]. Sesquiterpenoids are by far the major and most typical secondary metabolites found in the aerial parts and roots of *Stevia* species.

To our knowledge, phytochemical information is available to date on 61 *Stevia* species, sesquiterpene lactones (STLs), diterpenes, longipinanes, and flavonoids being the main types of compounds reported. Other phytochemical groups include triterpenes and sterols.

Sesquiterpene lactones are frequently found and isolated from different *Stevia* species [1]. This phytochemical group is the subject of numerous studies due to the extensive biological activities presented. It has shown anti-inflammatory, cytotoxic, antiviral, antimalarial, antileishmanial, and trypanocidal activities, among others [15,16].

The STLs most commonly reported in *Stevia* are germacranolides and guainolides, whose skeletons are considered to be of the first and second degree of biogenetic complexity, respectively. However, other skeletal types corresponding to the third degree of complexity such as xanthanolides, pseudoguaianolides, and eremophilanolides were also found. Both *trans*-fused lactones toward C-6 and *cis*-fused lactones toward C-8 were isolated from *Stevia* species [1]. A common characteristic of the STLs of this genus is the β-orientation of substituents at C-8. Among germacranolides, germacrolides, heliangolides, and melampolides have been described, the latter type being found exclusively in South American species [1,7]. In guaianolides, oxidation is frequently observed at the C-3, C-8, and C-14 positions and also at C-2. Pseudoguaianolides have been isolated only from Mexican species. A unique and specific structural type of STL found in *Stevia* is jujuyensolide, isolated from *Stevia jujuyensis* [1].

In *Stevia*, diterpenoids of the labdane, *ent*-labdane, *ent*-kaurane, and clerodane type have been described. Among them, the diterpenoids with kaurane skeleton predominate. The production of glycosidic diterpenes was also reported in species of this genus. In this sense, the most relevant compound is the sweetener stevioside, an *ent*-kaurane diterpene glycoside isolated from *S. rebaudiana*, as mentioned above.

Longipinanes are the other phytochemical group commonly found in *Stevia*. They are tricyclic sesquiterpenes that are frequently poliesterified. The positions C-7, C-8, C-9, and C-13 are usually oxidazed with acyloxy and/or hydroxyl groups and a keto function is present at C-1 which is α,β-unsaturated in most compounds [1]. Longipinenes are highly oxygenated tricyclic structures with a spatial configuration susceptible to different types of rearrangements [17]. The absolute configuration of most longipinanes isolated from *Stevia* spp. has been determined by chemical correlation with (+)-longipinene and by circular dichroism. The most common ester residues present in the structure are angelate, tiglate, senecioate, methacrylate, and acetate; however, other groups have also been described (epoxyangelate, isovalerate, isobutyrate) [7].

In *Stevia*, flavonoids are less frequently reported than sesquiterpene lactones and diterpenes. However, flavonoids are a group with well-known biological activities such as anti-inflammatory, antioxidant, antiarrhythmic, antihypertensive, antiviral, and antiprotozoal, among others. Most of the flavonoids reported in *Stevia* are flavones and flavonols and their glycosides, the flavonols being the most commonly produced as in other Eupatoriae genera. Flavonols and flavones have similar substitution patterns. Hydroxylation may occur at positions 5, 7, 3′, or 4′ for flavones and flavonols. This is the case, for example, for the flavones apigenin and luteolin and the flavonols kaempferol and quercetin, respectively. Polyhydroxylated and methoxylated flavones and flavonols in the A-ring are also found. Glycosylation can occur at positions 3, 7, or 4′, with glucose and galactose being the sugars most frequently found.

It is worth mentioning the differences found between the North and Central American species and the South American ones. The former have a higher amount of methoxylated aglycones and flavonoids glycosides of luteolin and quercetin than the *Stevia* species from South America.

### 3.2. Advances in the Chemistry of Stevia

As mentioned above, the chemistry of the *Stevia* genus was reviewed in 1998 and in 2002 [1,7]. Consequently, in this review, a survey of all the published literature on the chemistry, biological activity, and pharmacology of extracts and isolated compounds of this genus was carried out, covering the period from January 1998 to February 2021. According to the literature, in this period, 14 *Stevia* species were studied regarding their chemical composition (Table S1).

From the air-dried roots of *S. connata*, collected in Mexico, eight longipinenes (**1**–**8**) and stigmasterol (**9**) were isolated. The isolated compounds were longipinane-7β,8α,9α-triol-1-one-7-angelate-8-methylbutyrate (**1**), longipin-2-ene-7β,8α,9α-triol-1-one-8,9-diangelate (**2**), longipinane-7*α*,8β,9β-triol-1-one-7,9-diangelate (**3**), longipinane-7β,8α,9α-triol-1-one 7,8-diangelate (**4**), longipin-2-ene-7β,8α,9α-triol-1-one-7,8-diangelate (**5**), longipinane-7β,8α,9α-triol-1-one 8,9-diangelate (**6**), longipin-2-ene-7β,8α,9α-triol-1-one 8-angelate-9-methylbutyrate (**7**), and longipin-2-ene-7β,8α,9α-triol-1-one-7-angelate-8-methylbutyrate (**8**); (**1**), (**2**), and (**7**) being new natural compounds [18].

In 2000, Roman et al. [19] reported the isolation of grindelane diterpenoids from the leaves of Mexican *Stevia subpubescens*. Four new 9*R*,13*R*-epoxylabdane diterpenes (**10**–**13**) and a known clerodane derivative, 3,4β-epoxy-5β,10β-*cis*-17α,20α-clerod-13(14)-en-15,16-olide (**14**), were reported.

Two triterpenes, 8,14-*seco*-oleana-8(26),13-dien-3β-ol (**15**) and its acetyl derivative, 8,14-*seco*-oleana-8(26),13-dien-3β-ol acetate (**16**), were isolated from *Stevia viscida* and *Stevia eupatoria*, respectively [20].

The phytochemical study of *S. pilosa*, led to the isolation of longipinenes. The compounds (4*R*,5*S*,7*S*,8*S*,9*S*,10*R*,11*R*,2’’*S*)-7-angeloyloxy-9-hydroxy-8-(a-methyl-butyryloxy)-longipin-2-en-l-one (**17**) and (4*R*,5*S*,7*S*,8*R*,10*R*,11*R*,2’’*S*)-7-angeloyloxy -8-(a-methylbutyryloxy)-longipin-2-en-l-one (**18**), and other four logipinenes (**19**–**22**) were isolated from the roots of a Mexican collection of *S. pilosa* [21]. The presence of flavonoid glycosides of luteolin and quercetin, chromenes derivatives, longipinenes with angelate and methylbutyrate ester residues, polysaccharides (mainly glucose), and fatty acids was demonstrated in the methanol extracts of this species and in *S. eupatoria* [11].

In 2009, three longipinenes were isolated from *S. monardifolia* methanol extract: 7β,8α-diangeloyloxylongipin-2-en-1-one (**23**), 7β,8α-diangeloyloxylongipinan-1-one (**24**), and 7β-angeloyloxy-8α-isovaleroyloxylongipin-2-en-1-one (**25**) [22].

The new sesquiterpene lactone 1,5:3,4-diepoxyguaia-10(14)-en-12,8-olide (**26**) was described by Valdez-Calderón et al. [23]. This diepoxyguaianolide, isolated from the aerial parts of *S. tomentosa*, contains two β-oriented epoxide groups in the five-membered carbocyclic ring.

A chemical study of the Mexican *S. phlebophylla* was carried out by Ceunan et al. [24]. The phytochemical study of this plant led to the isolation of a new diterpene glycoside, 16β-hydroxy-17-acetoxy-*ent*-kauran-19-oic acid- (6-O-β-d-xylopyranosyl-β-d-glucopyranosyl) ester (**27**).

A bioguided fractionation of the dichloromethane extract of the Argentinean species *S. satureiifolia* var. *satureiifolia*, led to the isolation of three methoxylated flavones: eupatorin (**28**), cirsimaritin (**29**), and 5-desmethylsinensetin (**30**) [25].

Reis Simas et al. [26] reported the chemical composition of the essential oil of *S. serrata* collected in the highlands of Guatemala. The analysis of the essential oil, obtained by hydrodistillation, showed a high content of sesquiterpenes, chamazulene (**31**) (60.1%) being the major component. Other compounds identified were (E)-nerolidol (**32)**, caryophyllene oxide (**33**), and germacrene D (**34**).

From the aerial parts of *S. urticifolia*, four flavonoids were described [27]. The phytochemical investigation led to the isolation of hispidulin (5,7,4-trihydroxy-6-methoxyflavone) (**35**), nepetin (6-methoxyluteolin; 5,7,3,4-tetrahydroxy-6-methoxyflavone) (**36**), quercetin (3,5,7,3,4-pentahydroxyflavone) (**37**), and quercetin-3-O-α-L-arabinofuranoside (**38**).

Aerial parts of *S. subpubescens* var. *subpubescens*, collected in Mexico, were extracted consecutively with hexane, EtOAc, and MeOH to obtain the corresponding extracts. The purification of the extracts by chromatographic techniques led to the isolation of stigmasterol (**9**), the flavonoids 4′-*O*-methylsakuranetin (**39**), sakuranetin (**40**), 3,7,4′-*O*-trimethylkaempferol (**41**), ayanin (**42**), ermanin (**43**), hyperin (**44**), the labdanes cistenolic (**45**) and labdanolic (**46**) acids, the coumarins scoparone (**47**), melilotoside (**48**), L-*chiro* inositol (**49**), and mixtures of stigmasteryl and β-sitosteryl glucosides [28].

From the aerial parts of *Stevia jorullensis*, three sesquiterpene lactones, a germacranolide, 11β,13-dihydrocostunolide (**50**) and two eudesmanolides, 11,13-dihydroreynosin (**51**), and 1β-hydroxycolartin (**52**) were described. These sesquiterpene lactones were reported for the first time in the *Stevia* genus. Additionally, chlorogenic acid (**53**), β-sitosterol (**54**), stigmasterol (**9**), β-sitosteryl glucopyranoside (**55**), and stigmasteryl glucopyranoside (**56**) were also isolated [29].

The isolation of helenin from the aerial parts of *S. lucida* was reported by Chacón-Morales et al. [30]. The authors described the isolation and identification of helenin, a natural mixture of the isomeric eudesmanolides alantolactone (**57**) and isoalantolactone (**58**). This was the first report of this natural eudesmanolide mixture in *Stevia* and in the Eupatorieae tribe.

## 4. Biological Activity

Various biological activities of extracts and isolated compounds from the *Stevia* species have been reported. Some details of the pharmacological properties of *Stevia* extracts published up to February 2021 are presented. The activity of the compounds reported by Hernandez et al. [1], and of those published in the period January 1998–February 2021, is also described here. As evidenced by the literature, research on biological properties is primarily oriented by chemotaxonomic and ethnopharmacological aspects, among others.

### 4.1. Biological Activity of Stevia Extracts

Most of the pharmacological activities of extracts of *Stevia* species are related to antioxidant, antiparasitic, antiviral, anti-inflammatory, and antiproliferative activities.

There are numerous publications on the biological activities of *Stevia rebaudiana* Bertoni extracts. Taking into account all of the information available, the number of publications, and the potential of this species, a review dedicated exclusively to its pharmacological properties should be recommended. For this reason, only a few representative and thorough papers on *S. rebaudiana* were included in this update.

The oldest report about the biological activity of *Stevia* extracts corresponds to Fournet et al. [31]. In this study, the authors evaluated the antiprotozoal activity of Bolivian medicinal plants. The ethanol, ethyl acetate, and petroleum ether extracts of *Stevia yaconensis* were tested on *Leishmania brasiliensis*, *L. donovanni*, *L. amazonensis*, and *Trypanosoma cruzi*. The extracts were active in the range 50–100 µg/mL against the parasites.

The antimutagenic effect of the methanolic extracts obtained from the leaves, roots, and flowers of *S. pilosa* and *S. eupatoria* were evaluated by Cariño-Cortes et al. [11]. The authors found an inhibitory effect of both species on the mutagenicity induced by 2-aminoanthracene in the strain TA98. The best effect was observed with leaves of both species and the flowers of *S. eupatoria*. The mutations induced with N-ethyl-N’-nitro-N-nitrosoguanidine in the strain TA100 were also reduced. Extracts from flowers and roots of *S. pilosa* and *S. eupatoria*, respectively, showed an inhibition of about 93%. Using mitomycin-C on the strain TA102, a reduction of 87% with the extract of the leaves of *S. eupatoria* was obtained. The antioxidant potential of the extracts has also been demonstrated (>90%).

The antiviral properties of a dried extract of the leaves of *S. rebaudiana* were assessed by Kedik et al. [32]. At a concentration of 2000 µg/mL, the extract inhibited the reproduction of RNA-containing Teschen disease virus (porcine teschovirus) by 0.5 lg tissue cytopathic dose (TCD_50_), the DNA-containing infectious rhinotracheitis (IRT) virus (bovine herpesvirus 1) by 0.25 lg TCD_50_, and RNA-containing human coronavirus (Hco V-229E) by 0.33 lg TCD_50_. At a dose of 4000 µg/mL, the extract inactivated Teschen disease virus by 0.75 lg TCD_50_, IRT virus by 0.5 lg TCD_50_, and coronavirus by 0.66 lg TCD_50_.

Another investigation carried out with the aqueous leaf extract of *S. rebaudiana* demonstrated its antioxidant activity. The IC_50_ value of the aqueous extract in DPPH radical scavenging assay was 83.45 µg/mL. The total phenolic content of the aqueous leaf extract was 56.73 mg/g. The extract also inhibited the hydroxyl radical, nitric oxide, and superoxide anions with IC_50_ values of 100.86, 98.73, and 100.86 µg/mL, respectively [33].

The acetone extract of *S. rebaudiana* was evaluated for its hepato-protective efficacy on rats treated with CCl_4_ (carbon tetrachloride) as hepatotoxic. The extract showed the ability to suppress the elevation of serum ALT (*p* < 0.05) and AST (*p* < 0.001) activities. The administration of the extract allows the prevention of deleterious effects caused by CCl_4_, lowering lipid peroxidation, and the enhancement of antioxidant activities as SOD and CAT [34].

The trypanocidal activity of four Argentinean *Stevia* species was evaluated by Beer et al. [25]. The dichloromethane extracts of *Stevia satureiifolia* var *satureiifolia*, *S. aristata*, *S. multiaristata*, and *S. entreriensis* were assessed on *T. cruzi* epimastigotes. All the extracts showed antiprotozal activity at concentrations of 100 and 10 µg/mL. *S. aristata* and *S. satureiifolia* var. *satureiifolia* extracts were the most active with growth inhibition percentages of 87.3 ± 0.3% and 90.9 ± 1.2% at a concentration of 10 µg/mL.

Machado et. al. [27] evaluated the antioxidant capacity of the hexane, ethanol, and ethyl acetate extracts of *S. urticifolia*, using the DPPH assay. Crude extracts were considered active when IC_50_ < 500 g/mL. The ethanol and ethyl acetate extracts showed antioxidant capacity.

To determine the influence of the extraction solvent in the phenolic content, Medina Medrano et al. [35] macerated leaves of *S. ovata*, *S. origanoides*, and *S. viscida* with water, ethanol 50%, and ethanol 100%. The total phenolic content of each extract was determined by the colorimetric method and the antioxidant activity of the leaves extracts was evaluated using ABTS+ radical scavenging assay and DPPH free radical scavenging assay. The authors concluded that the samples with the highest phenolic content were those extracted with the solvent combination (ethanol–water). These same extracts showed greater antioxidant activity.

A three-arm single-blinded randomized crossover trial was developed by Farhat et al. in 2019 [36] to investigate the effect of the *Stevia* sweetener on postprandial glucose levels, appetite, and food intake, as a strategy for type 2 diabetes treatment. Participants received preloads of water, sugar (60 g), and *Stevia* (1 g) on three different days, followed by an ad libitum pizza lunch. The results showed that *Stevia* lowers appetite sensation and did not increase food intake or postprandial glucose levels.

The aerial parts of *S. subpubescens* var. *subpubecens* were extracted by Perez Castorena et al. in 2019 [28] for phytochemical analysis and evaluation of anti-inflammatory activity. Hexane, acetyl acetate, and methanol extracts were obtained. Anti-inflammatory activity was tested on the TPA model of induced acute ear edema. The different extracts at 1 mg/ear dose presented mild anti-inflammatory activity.

The anticancer activity of *S. pilosa* and *S. eupatoria* methanolic root extracts on prostate cancer cells was studied by Martinez-Rojo et al. [37]. The study was conducted on a human fibroblast cell line, and on androgen-dependent (LNCaP) and androgen-independent (PC-3) prostate cancer cell lines. The cell viability was evaluated using a Trypan Blue exclusion test for 48 h and the migration by a wound-healing assay. Both extracts significantly reduced the viability and migration of prostate cancer cells in all concentrations evaluated. The antiproliferative effect of the *Stevia* extracts was higher in cancer cells than in normal cells.

The essential oil of *S. serrata* demonstrated antinociceptive and anti-inflammatory activity in an in vivo experiment conducted by Cordeiro [14]. The essential oil was tested in chemical (capsaicin- and glutamate-induced licking response) or thermal (hot plate) models of nociception. The mechanism of action was evaluated using two receptor antagonists (naloxone, atropine) and an enzyme inhibitor (L-NAME). The antihyperalgesic effect was evaluated using carrageenan-induced nociception and evaluated in the hot plate. The data obtained suggested that the essential oil of *S. serrata* presents an antinociceptive effect mediated, at least in part, through activation of opioid, cholinergic, and nitrergic pathways.

Finally, the antioxidant activity of the aqueous extracts of seven wild plants collected from Peru was evaluated in 2021 by Gonzales et al. [38]. Among them, *S. macbridei* was tested. An in vitro DPPH assay was conducted along with the in vivo assay on the sensibility towards hydrogen peroxide of the yeast sod1 mutant. Both assays determined that *S. macbridei* aqueous extracts possess antioxidant activity.

### 4.2. Biological Activity of Compounds Isolated from Stevia Species

Biological activities regarding compounds isolated from *Stevia* species can be found in the literature, most of them being dedicated to the sweetener diterpene glycosides from *Stevia rebaudiana*.

Taking into consideration that sesquiterpene lactones, together with diterpenoids and flavonoids, are phytochemical groups of interest due to their pharmacological potential, details of these types of compounds strictly, isolated from *Stevia* spp., will be analyzed.

In this review we have focused on the activity of compounds isolated from *Stevia* species. However, it should be considered that many of these compounds can also be found in plant species of other genera and families.

#### 4.2.1. Biological Activity of Sesquiterpene Lactones

**Figure 1:**
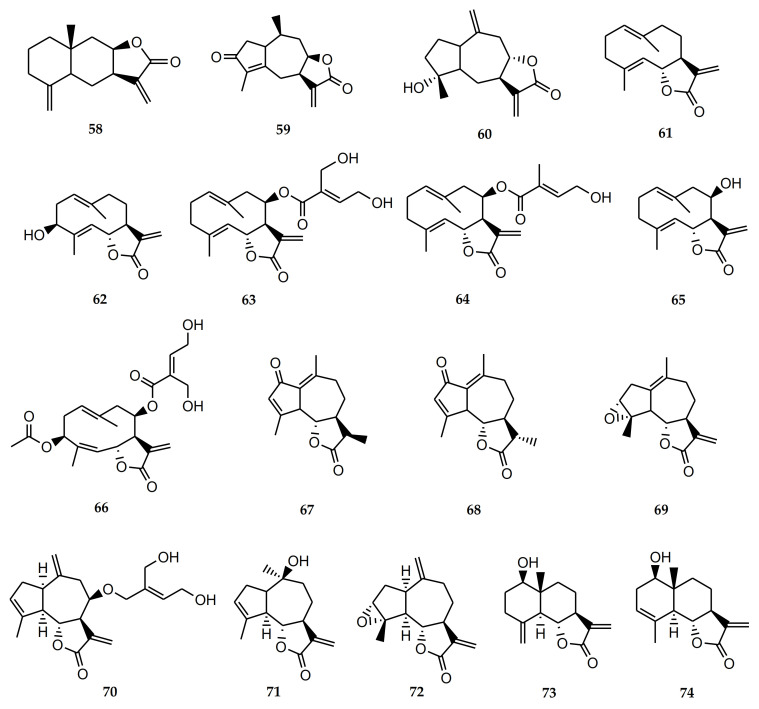
Chemical structures of bioactive sesquiterpene lactones isolated from *Stevia* species.

Sesquiterpene lactones (STLs) are one of the major phytochemical groups of compounds present in *Stevia*. Many of the biological activities attributed to medicinal plants and extracts can be related to the presence of this type of compound. Sesquiterpene lactones have a wide range of biological activities reported: antitumoral, antiparasitic, trypanocidal, leishmanicidal, antioxidant, neuroprotective, antiallergic, antidiabetic, anti-inflammatory, etc. [15,16]. The bioactive sesquiterpene lactones isolated from *Stevia* species are detailed in Table 2 and Figure 1.

The STL achalensolide (**59**) has been isolated from *S. achalensis, S. polyphylla*, and *S. satureifolia* [1]. The cytotoxic activity of this guaianolide has been tested in human tumor cell lines: U251 (glioblastoma), MCF-7 (breast), and SKLU-1 (lung). The IC_50_ values obtained were 9.5 ± 0.8, 9.5 ± 0.8, and 7.6 ± 0.3 µM, respectively. The anti-inflammatory activity was assessed in a mouse ear model of edema induced by TPA. Achalensolide presented an inhibition of 12.40% at 1 µM [39].

The isolation of the STL inuviscolide (**60**) from *S. achalensis*, *S. isomeca*, and *S. ovata* has been reported in the literature [1]. This compound showed inhibition of proinflammatory enzymes: elastase, cyclooxygenase 1, and secretory phospholipase A2. It was able to reduce the skin leukocyte infiltration in a murine model of dermatitis [40]. Iniviscolide has also shown anti-inflammatory in vivo activity in the ear and paw edema tests. The guaianoline-type sesquiterpene lactone inuviscolide reduced phospholipase A2-induced edema with an ID_50_ of 98 µmol/kg. In intact cells, it decreased the generation of leukotriene B4 (IC_50_ = 94 µM) [41]. This compound demonstrated cytotoxic activity against human melanoma cell lines, inhibiting the proliferation of the cell lines: SK-28, 624 mel, and 1363 mel in a dose-dependent manner. The compound also caused cell-cycle arrest at G2/M and induced apoptotic cell death [42].

Costunolide (**61**) is a germacranolide-type sesquiterpene lactone that has been found in the genus *Stevia* only from *S. amambayensis.* However, this compound has been obtained from many other Asteraceae species [41]. This STL exhibited antitumor activity against cancer cells. Its mode of action is related to the induction of apoptosis, regulation of the cell cycle, and inhibition of angiogenesis and metastasis. It also reversed the drug resistance mechanism [41].

Compound **61** was active against osteosarcoma and adenocarcinoma in xenografted mice. This STL has also shown potent anti-inflammatory activity, as well as antidiabetic, antihelminth, antimicrobial, antimycobacterial, antiulcer, and antioxidant effects [41,43]. Recent studies demonstrated the antiosteoarthritic and antiasthmatic effects of this sesquiterpene lactone. The evaluation of costunolide as an inducer of hair growth in mice was also proven [41].

Costunolide exhibited in vitro activity against the epimastigotes of *T. cruzi* with a minimum lethal concentration of 7 μM [44]. Costunolide was also active against amastigotes of *Leishmania mexicana* (IC_50_ = 9.4 µM). When testing against *Trypanosoma cruzi* amastigotes, it did not show significant activity. Cytotoxicity was assessed on Monkey Vero cells [45]. The antitrypanosomal activity of costunolide was also evaluated by Julianti et al. [46]. This sesquiterpene lactone was active against *Trypanosoma brucei rhodesiense* with an IC_50_ of 1.3 ± 0.4 µM and with a CC_50_ of 7.7 ± 1.3 µM in L6 cell, showing a selectivity index (SI) of 5.9.

Lee et al. [47] demonstrated the antiallergic effect of costunolide using in vitro and in vivo models. This STL was able to reduce the number of immune cells, mainly eosinophils, and diminished the expression and secretion of Th2 cytokines (IL-4 and IL-13) in the bronchoalveolar lavage fluid and lung tissues of mice with allergic asthma.

Compound **61** produced a hypolipidemic effect in streptozotocin-induced diabetic rats and would prevent osteoporosis by the enhancement of osteoblastic function [48,49]. Studies conducted by Ham et al. [50] demonstrated that costunolide produces a neuroprotective effect and inhibits dopamine-induced apoptosis. Thus, this sesquiterpene lactone has been considered a promising candidate for the treatment of neurodegenerative diseases such as Parkinson’s.

*Stevia grisebachiana* was one of the sources of the germacranolide hanphyllin (3β-hydroxycostunolide) (**62**). The cytotoxic activity of this compound was evaluated in cervical adenocarcinoma HeLa, breast adenocarcinoma MCF7, and skin epidermoid carcinoma A431 cells using the MTT assay. Hanphyllin showed moderate tumor cell-growth inhibitory activity with an IC_50_ of 14.95 ± 2.21, 12.67 ± 1.25, and 13.98 ± 1.38 μM, respectively [51]. Hanphyllin was also able to activate the antioxidant response element (related to neurodegenerative diseases) on primary mouse cortical cultures. The cytotoxycity of this STL was also evaluated. The viability of the cells was nearly 100% at concentrations of 12.5 and 25 µM [52].

Eupatoriopicrin (**63**) is a germacranolide-type sesquiterpene lactone that has been isolated from *S. alpina* var. *glutinosa*, *S. maimarensis*, *S. procumbens*, and *S. sarensis* [1]. This compound has been tested for its trypanocidal activity on epimastigotes, trypomastigotes, and amastigotes of *Trypanosoma cruzi* [53,54]. Eupatoriopicrin was active against the forms of the parasite with IC_50_ values of 4.39, 19.9, and 6.3 µM, respectively. On Vero cells, this STL presented a CC_50_ of 257.7 µM. Selectivity indexes were 12.9 and 40.6 for trypomastigotes and amastigotes, respectively. The administration of 1 mg/kg/day of eupatoriopicrin to infected mice produced a significant reduction in the parasitemia levels. Skeletal muscular tissues from eupatopicrin-treated mice displayed only focal and interstitial lymphocyte inflammatory infiltrates and small necrotic areas [54]. This sesquiterpene lactone has also demonstrated activity against *Trypanosoma brucei rhodesiense* with IC_50_ values of 1.2 and 1.4 μM and SI of 1.3 and 11.1, respectively [46,55].

Eupatoriopicrin also inhibited pro-inflammatory functions of neutrophils via suppression of IL-8 and TNF-α production (IC_50_ < 1 μM) and p38 and ERK 1/2 MAP Kinases. In an in vivo assay, it suppressed neutrophil migration in a thioglycolate-induced inflammation model [56]. Furthermore, it produced inhibition of the MurA enzyme of *Escherichia coli* and *Pseudomona aeruginosa* [57]. Eupatoriopicrin was also active on tumor cell lines [58,59].

Together with the activity reported for eupatoriopicrin, 5-deoxyeupatoriopicrin (**64**) from *S. chamaedrys* also demonstrated the capacity to suppress the production of inflammatory cytokines. [56].

Eupatolide (**65**) is a sesquiterpene lactone (germacranolide) isolated from *S. alpina* var. *glutinosa* [1]. This compound exhibited cytotoxicity against HL60 (IC_50_ = 2.91 µM), SMMC-7721 (IC_50_ = 2.46 µM), A-549 (IC_50_ = 2.86 µM), MCF-7 (IC_50_ = 2.91 µM), and SW-480 (IC_50_ = 3.01 µM) cancer cell lines. This sesquiterpene lactone also showed significant inhibitory activity against LPS-induced NO production in RAW264.7 macrophages with an IC_50_ of 2.00 µM [60]. Later, Boldbaatar et al. [61] demonstrated that eupatolide inhibited the proliferation, migration, and invasion of breast cancer cells and explained the possible molecular mechanism by which it exerted its activity.

The presence of the STL eucannabinolide (**66**) has been reported in *S. origanoides* and *S. sarensis* [1]. Eucannabinolide was active on *T. cruzi* with IC_50_ = 18 ± 3 µM (7.6 μg/mL). On *Leishmania infantum*, this compound was less active (IC_50_ > 25 µM) [62]. Eucannabinolide has also demonstrated activity against *Trypanosoma brucei rhodesiense* trypomastigotes (IC_50_ = 1.1 ± 0.1 µM). The cytotoxicity on mammalian cells (CC_50_) on the L6-cell line from rat-skeletal myoblasts was 7.8 ± 0.8 µM [55]. Eucannabinolide was also active on the parasite when it was loaded onto nanoparticles with a free drug equivalent IC_50_ value of 3.32 µM [63]. Eucannabinolide has also shown cytotoxic activity against tumor cell lines [64] and anti-inflammatory activity [65]. Recently, Zhu et al. [66] reported that this STL suppressed the growth and metastasis of triple negative breast cancer (TNBC) via inactivation of signal transducer and activator of transcription 3 (STAT3).

The guaianolide achillin (**67**) has been isolated from *Stevia alpina* var. *alpina* [1]. This guaianolide-type sesquiterpene lactone has shown anti-inflammatory, antiallergic (IC_50_ = 100 µM) and cytotoxic activity on SMMC-7721 cell lines [67]. Sanchez-Carranza et al. [68] demonstrated that achillin enhances the cytotoxic effect of paclitaxel and the induction of G2/M phase cell cycle arrest and apoptosis when associating both drugs. This guainolide also reduced P-gp levels and increased the intracellular retention of doxorubicin in Hep3B/PTX cells. This compound was moderately active and selective on *Trypanosoma cruzi* epimastigotes with an IC_50_ of 41.24 µM and a SI of 3.52 [53].

Leukodin or desacetoxymatricarin (**68**) is a sesquiterpene lactone that has been obtained from *S. pilosa* [1]. This compound has shown antiallergic effects [67] and has inhibited melanin pigment synthesis and tyrosinase activity in B16F10 melanoma cells [69]. This sesquiterpenelactone also inhibited meiosis in oocytes of amphibians [70].

From *S. yaconensis* var. *subeglandulosa*, the guaianolide ludartin (**69**) has been isolated [1]. Ludartin inhibited the viability, migration, and proliferation of osteosarcoma cell lines and it also increased cell apoptosis, with an IC_50_ 15–30 µM. The highest effects were on the Saso-2 osteosarcoma cells, with an IC_50_ of 15 µM. On normal hFOB 1.19 osteoblasts, this compound showed minor cytotoxic effects (IC_50_ > 100 µM). Ludartin exerted cell cycle arrest at the G2/M checkpoint [71]. This sesquiterpene lactone also showed effects on spinal cord injury in a rat model. Treatment with this compound improved locomotion by the inhibition of inflammatory cytokine expression and prevention of cell apoptosis [72].

Ludartin has been reported to have action on estrogen biosynthesis. The STL inhibited aromatase enzyme activity in human placental microsomes (IC_50_ = 55 µM), being a competitive inhibitor with a *K*_i_ = 23 μM [73]. Previously, Giordano et al. [74] demonstrated that this compound showed cytoprotective effects against the development of peptic ulcers, after an oral dose of 40 mg/kg.

Eupahakonenin B (**70**) is another guaianolide-type sesquiterpene lactone that has been isolated from different *Stevia* species: *S. alpina* var. *glutinosa*, *S. chamaedrys*, *S. gilliesii*, *S. mercedensis*, *S. procumbens*, *S. sarensis*, *S. satureiifolia*, and *S. setifera* [1]. This molecule has shown activity against *T. cruzi* epimastigotes, trypamoastigotes, and amastigotes with IC_50_ values of 0.78, 33.1, and 89.4 µM, respectively [53,54].

The sesquiterpene lactone 10-epi-8-deoxycumambrin B (**71**) was obtained from *S. grisebachiana* and *S. yaconensis* var. *subeglandulosa*. This guaianolide inhibited the aromatase enzyme activity in human placental microsomes (IC_50_ = 7 µM), being a competitive inhibitor with a *K*_i_ = 4 μM. 10-epi-8-deoxycumambrin B acted as type II ligand to the heme iron present in the active site of aromatase cytochrome P450 [73]. Later on, Luo et al. [75] calculated the molecular geometries and electronic structure of 10-epi-8-deoxycumambrin B as an aromatase inhibitor. Taking into account the correlation analysis, ELUMO (energy of lowest unoccupied molecular orbital) would have a positive impact on the inhibition activity.

Estafietin (**72**) is another guainolide isolated from *S. alpina* var. *alpina*, *S. boliviensis*, *S. grisebachiana*, and *S. yaconensis* [1]. The anti-*Trypanosoma cruzi* and anti-*Leishmania* activity of this STL, as well as the synthesis of derivatives, has been described [53,54,76]. Estafietin was active and selective on *T. cruzi* epimastigotes (IC_50_ = 0.24 μM, IS = 1789.25). On trypomastigotes and amastigotes, this compound showed IC_50_ of 117.5 and 109.3 μM. Estafietin was also active against promastigotes of *L. braziliensis* (IC_50_ = 1.0 μg/mL). This compound selectively inhibited T cell receptor activation [77] and showed an in vitro inhibitory effect on the meiosis reinitiation of amphibian oocytes [70].

In the *Stevia* genus, the sesquiterpene lactone isoalantolactone (**58**) has been obtained from *S. polyphylla and S. lucida* [1,28]. This compound has many biological activities described, one of them being the cytotoxic activity. Isoalantolactone has been proven to inhibit proliferation by the induction of apoptosis, autophagy, causing G1 phase arrest, or the activation of reactive oxygen species in gynecologic cancer cells, breast cancer, leukemia, and lung squamous cancer [78,79,80,81,82,83,84]. This compound induced apoptosis by targeting multiple cellular signaling pathways. The studies suggest that the simultaneous targeting could determine the effectiveness and selectivity in killing cancer cells [85].

In 2020, Yan et al. [86] reported the effect of isoalantolactone on pancreatic cancer cells lines, PANC-1 and SW-1990, from human pancreatic carcinoma. This STL inhibited the proliferation of PANC-1 and SW1990 cells at 48 h treatment. The IC_50_ was 3.75 and 3.15 µg/mL for isoalantolactone in PANC-1 and SW1990 cells, respectively.

Isoalantolactone showed synergistic effects against 21 β-lactamase-positive *S. aureus* strains (including methicillin-resistant *S. aureus*, when combining with penicillin G). The association of drugs was also effective on *S. aureus*-infected mice, increasing the survival rate (88.24%) after 144 h treatment [87].

Isoalantolactone has been able to inhibit osteoclastogenesis, without affecting osteogenesis, which is promising for osteoporosis treatment and other metabolic bone diseases [88]. Yuan et al. [89] evaluated the effect of the sesquiterpene lactone on lung inflammation, using a mouse model of acute lung injury. This compound was able to diminish the injury of lung tissues induced by lipopolysaccharide (LPS) and also reduced the production of inflammatory cytokines TNF-α and IL-1β, among other effects.

The trypanocidal activity of isoalantolactone was reported by Schmidt et al. [90]. This compound showed activity against *Trypanosoma cruzi* and *T. brucei rhodesiense* with an IC_50_ of 22.26 and 23.62 µM, respectively. On L6 cells (rat skeletal myoblasts), the CC_50_ was 3.97 µM.

Reynosin (**73**) is a sesquiterpene lactone isolated from *S. chamaedrys*. Turk et al. [91] evaluated the effect of this compound on the NF-κB transcriptional activity induced by LPS in RAW 264.7 macrophages cells. Reynosin presented an IC_50_ of 13.9 ± 1.6 µM.

Reynosin also showed mycobactericidal activity with a minimal bactericidal concentration (MBC) of 128 µg/mL against the H37Rv, 366-2009, and 104-2010 Mtb strains and a minimal inhibitory concentration (MIC) of 64, 64, 128, 128, and 128 µg/mL against the H37Rv, 104-2010, 63-2009, 366-2009, and 430-2010 Mtb strains, respectively [92].

The hepatoprotective effect of reynosin was evaluated in vitro and in vivo by Lim et al. [93]. The compound inhibited thioacetamide-induced apoptosis in primary hepatocytes and in a mouse model.

The effects of reynosin on dopamine (DA)-induced neuronal toxicity and the regulation of E6-associated protein and α-synuclein proteins were evaluated on in vitro and in vivo models of Parkinson’s disease. The compound showed a protective effect against DA-induced cell death [94].

Santamarine (**74**) has been isolated from *S. chamaedrys* [1]. The anticancer activity and its mechanism of action on HepG2 cells have been evaluated by Mehmood et al. [95]. This compound inhibited proliferation and induced apoptosis with an IC_50_ ~70 μM. The sesquiterpene lactone promoted reactive oxygen species (ROS) generation, diminished the activity of thioredoxin reductase (TrxR), produced depletion of glutathione (GSH) and mitochondrial membrane potential (ΔΨm) dissipation, modulation of Bcl-2 family proteins, cytochrome C release, caspases-9, -8, and -3 activation, and PARP cleavage.

Santamarine showed anti-inflammatory activity on lipopolysaccharide (LPS)-induced macrophages cells. This compound acted through the expression of heme oxygenase-1 [96]. This sesquiterpene lactone was also assessed by measuring the effect on the NF-κB transcriptional activity induced by LPS. Santamarine exhibited an IC_50_ value of 9.2 ± 0.5 µM [91].

Santamarine was active on *Mycobacterium tuberculosis* (Mtb) with a minimal bactericidal concentration (MIC) of 128 µg/mL against the H3Rv and 104-2010 Mtb strains and MICs of 128 µg/mL against the H37Rv, 366-2009, and 104-2010 Mtb strains [92].

**Table 2:** Sesquiterpene lactones isolated from *Stevia* species with biological activity reported.

Comp. N°	Common Name	Species	Reported Activity
**58**	Isoalantolactone	*S. polyphylla, S. lucida*	Antineoplastic. Antitumor. Antimicrobial. Anti-inflammatory. Anti-*Trypanosoma cruzi* and *T. brucei.* Inhibits osteoclastogenesis [85,86,87,88,89,90].
**59**	Achalensolide	*S. achalensis*, *S. polyphylla*, *S. satureifolia*	Anti-inflammatory [39].
**60**	Inuviscolide	*S. achalensis*, *S. isomeca*, *S. ovata*	Anti-inflammatory. Cytotoxic against melanoma cells [40,41,42]
**61**	Costunolide	*S. amambayensis*	Anti-inflammatory. Antitumor. Anti-*Trypanosoma.* Anti-*Leishmania.* Antioxidant. Antipyretic. Neuroprotecive. Antiallergic. Osteoporosis prevention. Antimycobacterial. Anti-*Helicobacter pylori*. Normoglycemic. Hypolipidemic [41,42,43,44,45,46,47,48,49,50].
**62**	Hanphyllin	*S. grisebachiana*	Antitumoral. Antioxidant [51,52].
**63**	Eupatoriopicrin	*S. alpina* var. *glutinosa*, *S. maimarensis*, *S. procumbens*, *S. sarensis*	Anti-*Trypanosoma cruzi*. Anti-*T. brucei.* Anti-*Leishmania.* Anti-*P. falciparum.* Anti-inflammatory. Antitumor. Antibacterial [46,53,54,55,56,57,58,59].
**64**	5′deoxy-eupatoriopicrin	*S. chamaedrys*	Anti-inflammatory [56].
**65**	Eupatolide	*S. alpina* var. *glutinosa*	Antimetastatic. Antineoplastic [60,61].
**66**	Eucannabinolide	*S. origanoides*, *S. sarensis*	Anti-*T. brucei.* Anti-inflammatory. Antibacterial. Antimetastatic [55,62,63,64,65].
**67**	Achillin	*S. alpina* var. *alpina*	Anti-*Trypanosoma cruzi*. Antineoplastic. Antitumor. Anti-inflammatory. Antiallergic [43,67,68].
**68**	Leukodin o desacetoxymatricarin	*S. pilosa*	Antiallergic. Inhibitory activity on melanoma cells. Meiosis inhibition in oocytes of amphibians [67,69,70].
**69**	Ludartin	*S. yaconensis var. subeglandulosa*	Antineoplastic. Anti-inflammatory. Gastric cytoprotective. Aromatase inhibition [71,72,73,74].
**70**	Eupahakonenin B	*S. alpina* var. *glutinosa*, *S. chamaedrys*, *S. gilliesii*, *S. mercedensis*, *S. procumbens*, *S. sarensis*, *S. satureiifolia*, *S. setifera*	Anti-*T. cruzi* [53,54].
**71**	10- epi-8- deoxycumambrin B	*S. grisebachiana*, *S. yaconensis* var. *subeglandulosa*	Inhibition of aromatase [73,75].
**72**	Estafietin	*S. alpina* var. *alpina*, *S. boliviensis*, *S. grisebachiana*, *S. yaconensis*	Anti-*T. cruzi.* Anti-*Leishmania brasiliensis.* Immunomodulator [53,54,76,77].
**73**	Reynosin	*S. chamaedrys*	Anti-inflammatory. Antimycobacterial. Hepatoprotective. Protective effect against dopamine-induced neuronal cell death [91,92,93,94].
**74**	Santamarine	*S. chamaedrys*	Antitumor. Anti-inflammatory. Antimycobacterial [91,92,95,96].

#### 4.2.2. Biological Activity of Diterpenes

**Figure 2:**
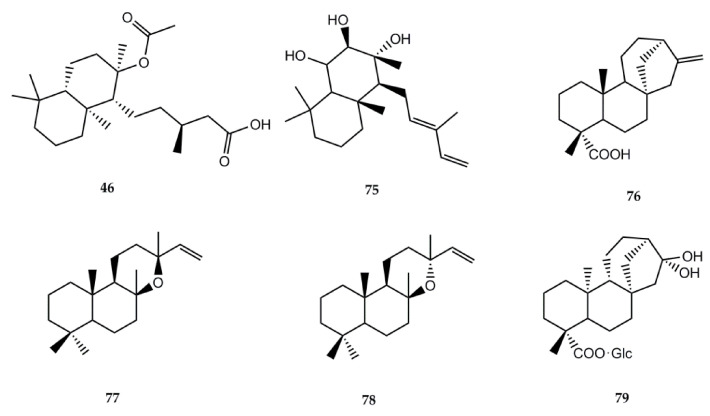
Chemical structures of bioactive diterpenes isolated from *Stevia* species.

The information regarding biological activity of diterpenes isolated from *Stevia* is lower than that of sesquiterpene lactones. The structures of the compounds and the biological activities can be found in Figure 2 and Table 3, respectively.

Labdanolic acid (**46**) has been isolated from *S. salicifolia* [1] and *S. subpubescens* var. *subpubescens* [28]. In 2007, Jayaprakasam et al. [97] assayed the compound for its anti-inflammatory activity using cyclooxygenase-1 (COX-1) and cyclooxygenase-2 (COX-2) enzymes. At 100 ppm, compound **46** showed selective COX-2 enzyme inhibition by 43%.

Austroinulin (**75**) was isolated from *S. rebaudiana* [1]. Its anti-inflammatory effects were documented by Cho et al. [98]. The authors tested the effects of austroinulin on nitric oxide (NO) production and its molecular mechanism in LPS-stimulated RAW264.7 macrophages. Results showed that this terpenoid inhibits NO production and iNOS expression by blocking the activation of STAT1, IRF3, and NF-кB in LPS-stimulated RAW264.7 macrophages. Another investigation also reported this activity, proving that austroinulin inhibited the enhanced production of nitric oxide (NO) and inducible nitric oxide synthase (iNOS) expression in RAW264.7 cells (10 μg/mL = 67.9 and 45.1%, respectively) [99].

Kaurenic acid (**76**) has been extracted from *S. monardaefolia* and *S. setifera* [1]. Its antiprotozoal activities were investigated by several authors. In 2002, Vieira et al. [100] first reported the in vitro trypanocidal activity towards *Trypanosoma cruzi* trypomastigote erythrocytic forms. In 2012, Brito et al. [101] developed in vitro and in vivo assays against *L. braziliensis*. The compound had a lethal effect on axenic amastigotes and promastigotes with DL_50_ of 0.25 and 0.78 µg/mL, respectively. Low toxicity was observed on J774-G8 macrophages with a DL_50_ of 25 µg/mL and high viability (70–92%), while a moderate viability was observed for infected macrophages (37–81%), with concentrations of 25 µg/mL or less. Additionally, a 70% reduction was observed in the size of the skin lesions in Balb/c mice with no evident toxic effect.

The antimalarial activity of compound **76** was assessed by Villasmil et al. [102]. In vitro testing measured its capacity to inhibit the formation of β-hematin, with 73.5% inhibition. The in vivo assay on mice showed an 8.5% parasitemia reduction at the 4th day post infection.

To determine the antioxidant effect of compound **76**, Mendoza et al. [103] conducted an in vivo experiment to test the compound on induced fatty liver mice. Gathered data suggest that kaurenic acid (**76**) acts as an antioxidant and reduces the genesis of lipid peroxidation. In 2020, Sarwar et al. [104] published a review on the anticancer effects of *ent*-kauranes in which they described that compound **76** exhibited antimelanoma effects with an IC_50_ value of 0.79 µM in B16F1 cells. The in vivo study showed that **76** (160 mg/kg) markedly reduced tumor sizes (49.51%) in a C57BL/6 mice model.

The anti-inflammatory and antipyretic biological activities of compound **76** were documented by Sosa et al. [105]; they determined anti-inflammatory activity in rats using egg albumin-induced paw edema (acute test) and Freund’s complete adjuvant-induced paw edema (subacute test), whereas the antipyretic effect was studied in rabbits by peptone-induced pyresis.

A small number of biological activities has been reported for the labdane type diterpene manoyl oxide (**77**) isolated from *Stevia berlandieri* [1]. Radical scavenging activity was reported by Venditti et al. [106]. Manoyl oxide was also able to inhibit prostaglandin E2 generation in cultured mouse peritoneal macrophages stimulated by zymosan, ionophore A23187, melittin, and PMA. Results showed that the compound interacts with the eicosanoid system [107].

The diterpenoid epi-manoyl oxide (**78**) isolated from *Stevia salicifolia* showed cytotoxicity against different cancerous cell lines [108]. The study, using MTT assay, revealed that epi-manoyl oxide was active against lung cancer cell line A549 and breast cancer cell lines MCF-7 and MDA-MB-231, with IC_50s_ = 19.37, 15.79, and 22.24 µM, respectively.

The *ent*–kaurane type diterpene paniculoside IV (**79**) isolated from *Stevia paniculata* [1] showed α-glucosidase activator activity. In vitro quantitative results on paniculoside IV against α-glucosidase showed an IC_50_ of 406.7 ± 20 mM [109].

#### 4.2.3. Biological Activity of Flavonoids

**Figure 3:**
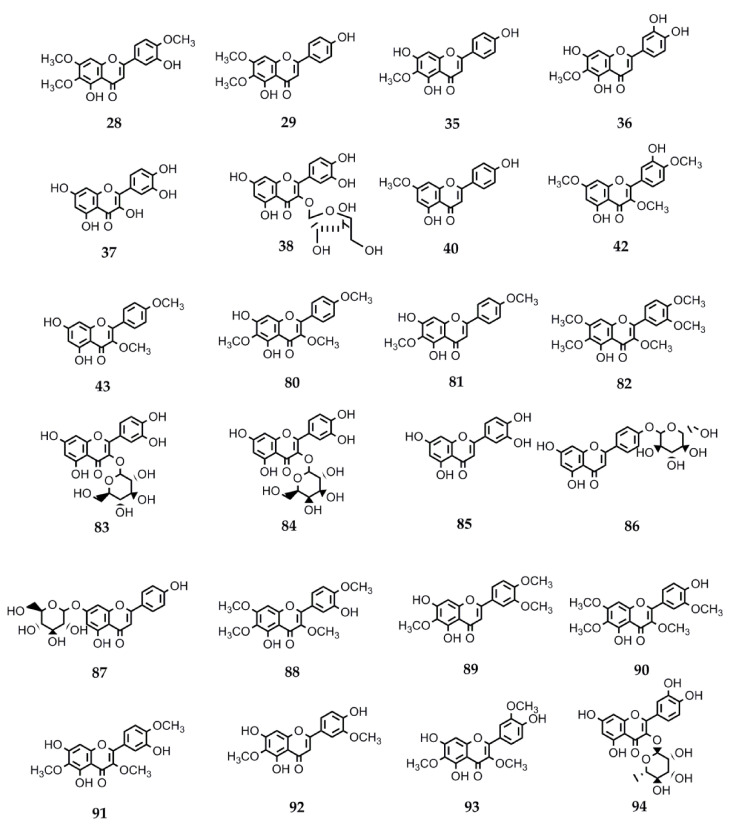
Chemical structures of bioactive flavonoids isolated from *Stevia* species.

Flavonoids constitute another relevant phytochemical group found in the genus *Stevia*. These polyphenols have shown several and diverse biological activities. Therefore, the interest in their pharmacological properties has increased significantly in the recent decades and currently, a lot of information is available. In this review, we collected the data on the biological activities of flavonoids isolated from *Stevia* spp. Many of these compounds can be found in other genera of Asteraceae as well as in members belonging to other families. The information about the activity and structures of the flavonoids is summarized in Table 4 and Figure 3.

Various biological activities have been reported for the flavonoid eupatorin (**28**) (3′,5-dihydroxy-4′,6,7-trimethoxyflavone), isolated from *S. satureiifolia* var. *satureiifolia*, *S. breviaristata*, *S. procumbens*, and *S. vaga* [1]. The antimycobacterial activity of eupatorin was reported by Castellar et al. [110]. The compound showed activity against *Mycobacterium tuberculosis* H37Rv with a MIC = 50 µg/mL.

The trypanocidal and leishmanicidal activities of compound **28** were evaluated by Beer et al. [25]. Eupatorin showed IC_50_ values of 0.2 μg/mL and 61.8 μg/mL on *T. cruzi* epimastigotes and trypomastigotes, respectively. Nevertheless, this compound was not active agains the amastigote forms of the parasites. The flavone showed activity against *L. braziliensis* promastigotes (IC_50_ = 55.1 μg/mL). This compound showed no cytotoxicity on Vero cells up to a concentration of 500 µg/mL.

Shafaei et al. [111] evaluated the in vitro angiotensin-converting enzyme (ACE) inhibition activity of different flavonoids. In vitro ACE inhibition activity was determined by measuring the concentration of hippuric acid (HA) formation from an ACE-specific substrate [hippuryl-histidyl-leucine (HHL)] by the action of ACE enzyme using a high performance liquid chromatography method. Among the tested flavonoids, compound **28** demonstrated the highest inhibition against ACE with IC_50_ 15.35 ± 4.49 µg/mL and binding ability with Zn (II) (56.03 ± 1.26%). ACE inhibition activity is directly related to compounds’ ability to bind with zinc ions in the active site of ACE enzyme.

The vasorelaxant activity and the underlying mechanisms of action of eupatorin were investigated by Yam et al. [112]. The study demonstrated that eupatorin exerts a vasorelaxant effect in thoracic aortic rings isolated from Sprague Dawley rats through the NO/sGC/cGMP and PGI2 pathways, calcium and potassium channels, and muscarinic and beta-adrenergic receptors.

Lee et al. [113] characterized the cytotoxic effect of compound **28** in HeLa cervical carcinoma cells. They suggested that this flavone would induce G2/M cell cycle arrest through the deregulation of cell cycle regulatory proteins and triggers apoptosis through the activation of the p53-dependent and p53-independent pathways.

Eupatorin has also been proposed as a potent candidate for an anti–breast cancer agent. Abd Razak et al. [114] evaluated the antitumor effect of **28** in 4T1-challenged mice by MTT assay. The study demonstrated that eupatorin was effective for delaying the 4T1-induced breast tumor growth in the animal model at the highest dosage of 20 mg/kg BW. This study showed the in vivo efficacy and the potential of eupatorin for breast cancer therapeutic purposes.

The antidiabetic and antiparasitic activities of eupatorin were evaluated by Gulcin et al. [115]. Eupatorin was tested, among others, for the inhibition of α-amylase and α-glycosidase enzymes to determine if they can reduce the level of glucose uptake in diabetes therapy. Eupatorin showed an effective inhibition profile with IC_50_ values of 175.01 nM and of 365.50 nM for the inhibitory potential of α-amylase and α-glycosidase, respectively. For the glutathione transferase enzyme, this phenolic compound showed an IC_50_ of 23.88 µM.

Numerous biological activities are reported for the flavonoid santin (5,7-dihydroxy-3,6-dimethoxy-2-(4-methoxyphenyl)-4H-chromen-4-one) (**80**) isolated from *S. microchaeta*, *S. monardifolia*, and *S. origanoides* [116]. In vitro trypanocidal and leishmanicidal activities of santin were reported by Sülsen et al. [117]. The IC_50_ values on *Trypanosoma cruzi* epimastigotes and trypomastigotes were 47.7 and 42.1 µM, respectively. Santin was also active against promastigotes of *Leishmania mexicana* (IC_50_ = 32.5 µM). Antiplasmodial activity of flavanol santin was evaluated by Melaku et al. [118]. This compound was found to be active against *Plasmodium berghei*-infected mice when tested in vivo using Peter’s four-day suppressive method. Santin was found to induce inhibition of parasitemia by 85.50% and 80.95% at doses of 100 mg/kg and 50 mg/kg, respectively.

Teffo et al. [119] evaluated the antioxidant potential of the compound **80** using a DPPH spectrophotometric assay and determined the antibacterial activity using a serial dilution microplate technique. Santin exhibited a weak antioxidant activity, at the highest concentration (200 µM) assayed, barely showing an antioxidant activity of 8.23%. The minimum inhibitory concentration (MIC) against *Staphylococcus aureus*, *Enterococcus faecalis*, *Escherichia coli*, and *Pseudomonas aeruginosa* varied from 63 µg/mL to 125 µg/mL.

Santin showed significant inhibitory activity on tubulin polymerization. When Mai et al. [120] performed a screening test of several flavonoids, santin was the strongest inhibitor of tubulin polymerization (IC_50_ = 5.7 mM). Furthermore, this compound was not toxic, even at high concentrations, to human peripheral mononuclear blood cells and mice lymphoid cells, while it exhibited strong cytotoxicity against various cancer cell lines. When the antiparasitic activity was evaluated in vitro against *T. brucei gambiense*, good inhibition levels were observed at 50 mM. Zhong et al. [121] demonstrated that this flavonoid compound inhibits influenza A virus replication through regulating MAPKs and NF-κB pathways. Santin showed anti-influenza activity in MDCK and THP-1 cells. Mechanistic studies revealed that santin depressed the phosphorylation of p38 MAPK, JNK/SAPK, ERK, and NF-κB factor and subsequently attenuated the expression of inflammatory cytokines in IAV-infected cells.

*Stevia subpubescens* var. *subpubescens* led to the isolation of sakuranetin (**40**) (5-hydroxy-2-(4-hydroxyphenyl)-7-methoxy-4H-chromen-4-one) [28]. Several biological activities have been reported for this compound. A review of sources and pharmacological aspects of sakuranetin performed by Stompor, M. [122] describes sakuranetin as having antiproliferative activity against the typical human cell lines of melanoma B16BL6, esophageal squamous cell carcinoma (ESCC), and the colon cancer (Colo 320). Moreover, it shows antiviral activity towards human rhinovirus 3 and influenza B virus, and is reported to have antioxidant, antimicrobial, anti-inflammatory, antiparasitic, antimutagenic, and antiallergic properties.

Ugocsai et al. [123] showed that sakuranetin (**40**) inhibits tumor growth through the apoptosis pathway both in vitro and in vivo in colon cancer cells expressing MDR1/LRP.

Park et al. [124] observed that compound **40** inhibits the growth of human colon carcinoma (HCT-116) cells with an IC_50_ value of 68.8 ± 5.2 μg/mL. Drira and Sakamoto [125] reported that 15 μmol/L of sakuranetin had cytotoxic effects on B16BL6 melanoma cells (MTT assay, after 72 h of treatment). They proved that sakuranetin influences melanogenesis inhibiting the ERK1/2 and PI3K/AKT signaling pathways, involved in the regulation of proliferation, differentiation, and apoptosis. In this study, the upregulating effect of sakuranetin on tyrosinase, tyrosinase-related protein 1, and tyrosinase-related protein 2 was also proven.

Hong and Ying [126] found that sakuranetin has strong effects on the inhibition of cell proliferation in esophageal squamous cell carcinoma by inducing DNA damage as well as mitochondrial membrane potential loss in esophageal cancer cells.

The antifungal activity of sakuranetin was demonstrated by Grecco et al. [127]. This compound inhibited the growth of all tested *Candida* strains by 98% and 99% at a concentration of 0.63 μg/μL, except *C. albicans* which was found to be more sensitive at 0.32 μg/μL (99% of inhibition). *Cryptococcus* species displayed a similar behavior: in the presence of 0.32 μg/μL of sakuranetin, *C. neoformans* serotype A (var. *grubii*) and *C. gatti* (R265) strains were inhibited by 99% and 97%, respectively. Strain *C. neoformans* serotype D (JEC21) showed 98% inhibition with a concentration of 0.08 μg/μL.

Pacciaroni et al. [128] reported that sakuranetin showed activity against standardized *Trichophyton rubrum* (MIC = 31.2 μg/mL) as well as clinical isolates of *T. rubrum* and *T. mentagrophytes* (MIC ranges 31.2–62.5 μg/mL and 31.2–125 μg/mL, respectively). It was demonstrated that this flavonoid not only possesses fungistatic but also fungicidal properties.

Zhang et al. [129] described compound **40** as a competitive inhibitor of the β-hydroxyacyl-acyl dehydratase carrier protein from *Helicobacter pylori* (HpFabZ) (IC_50_ = 2.0 ± 0.1 μM). Additionally, they showed that sakuranetin inhibited the growth of *Helicobacter pylori* ATCC 43,504 with a minimum inhibitory concentration (MIC) of 92.5 μM using the standard agar dilution method.

Sakuranetin showed antiprotozoal activity against *Leishmania amazonensis*, *L. braziliensis*, *L. major*, and *L. chagasi*, with a range of IC_50_ values 43–52 µg/mL, as well as against *T. cruzi* trypomastigotes, with an IC_50_ value of 20.17 µg/mL [130]. Consistent with Quintanilla-Licea et al. [131], compound **40** also presented antiprotozoal activity against *Entamoeba histolytica* (IC_50_ = 44.51 µg/mL).

Kwon et al. [132] described the activity of sakuranetin against the influenza B/Lee/40 virus. They reported a decrease in the cytopathic effect caused by viral invasion with an IC_50_ of 7.21 μg/mL. Furthermore, Choi [133] reported activity against human rhinoviruses HRV3 obtained from ATCC (American Type Culture Collection, Manassas, VA, USA) and propagated in human epithelioid carcinoma cervix (HeLa) cells. The compound exhibited an antiviral activity of approximately 67% against HRV3 at 100 mg/mL and of approximately 41% at 10 mg/mL.

Several authors investigated the anti-inflammatory activity of compound **40**. Bittencourt-Mernak et al. [134] reported that treatment with sakuranetin reduced the neutrophils in the peripheral blood and in the bronchial alveolar lavage of mice treated. It also reduced macrophage populations and keratinocyte-derived chemokines (IL-8 homolog) and NF-κB levels, collagen fiber formation, MMM-9 and TIMP-1-positive cells, and oxidative stress in lung tissues compared with LPS animals treated with vehicle. Sakuranetin treatment also reduced total protein and TNF-α and IL-1β levels in the lung.

Sakoda et al. [135] reported that compound **40** reverses vascular peribronchial and lung parenchyma remodeling in a murine model of chronic allergic pulmonary inflammation. They demonstrated that in vivo sakuranetin treatment with a dose of 20 mg/kg/BALB/c in mice reduced serum IgE levels, lung inflammation (eosinophils, neutrophils, and Th2/Th17 cytokines), and respiratory epithelial mucus production in ovalbumin-sensitized (for 30 days) animals in a murine experimental asthma model.

Previously, Taguchi et al. [136] investigated the anti-inflammatory and antioxidant effects of sakuranetin in lung disease using an experimental model of emphysema induced via the instillation of elastase into C57BL6 mice. Reductions in lung inflammation associated with attenuated lung parenchymal remodeling and alveolar destruction were observed in the sakuranetin-treated emphysematous animals.

Toledo et al. [137] observed that sakuranetin decreased IgE specific antibodies, eosinophil inflammation, AHR, and airway remodeling by reducing oxidative stress, Th2 pro-inflammatory cytokines and chemokines, and NF-κB activation in inflammatory cells in an experimental asthma model. Recently, Yamauchi et al. [138] observed that sakuranetin significantly inhibited NO induction and inducible nitric oxide synthase (iNOS) expression in rat hepatocytes. Moreover, this compound decreased the expression of type 1 IL-1 receptor gene and phosphorylation of Akt (protein kinase B), which is regulated by phosphatidylinositol-4,5-bisphosphate 3-kinase (PI3K). In addition, sakuranetin decreased the phosphorylation of the activator of isoforms of the CCAAT/enhancer-binding protein β (C/EBPβ), which synergistically activates the transcription of the iNOS gene with nuclear factor κB (NF-κB). Consequently, sakuranetin inhibited the co-activating activity of C/EBPβ with NF-κB, leading to the suppression of iNOS gene expression in hepatocytes. Zhang et al. [139] reported that sakuranetin had potent inhibitory activity against COX-1 (IC_50_ 196.1 μM). Hernández et al. [140] demonstrated that sakuranetin inhibits the production of leukotrienes, the strongest inflammatory mediators. It acts as the selective inhibitor of 5-lipoxygenase, the enzyme responsible for their synthesis.

The role of sakuranetin in Alzheimer’s disease was evaluated by Chen Li et al. [141]. The authors proposed that this flavonoid may exert protective effects on brain cells through an antioxidation mechanism.

The flavone pectolinaringenin (**81**) was isolated from *S. lucida* [1]. Pectolinaringenin displayed activity against the trypomastigote forms of *Trypanosoma cruzi*, exhibiting 50% inhibitory concentration (IC_50_) values of 51.61 µg/mL, when it was evaluated in vitro by the colorimetric MTT method after 24 h incubation by Grecco et al. [142]. Muthu et al. [143] evaluated the larvicidal activity of pectolinaringenin against *Culex quinquefasciatus* Say and *Aedes aegypti* L. The compound showed LC_50_ and LC_90_ values of 0.62, 2.87 ppm and 0.79, 5.31 ppm against *C. quinquefasciatus* and *A. aegypti*, respectively.

Cirsimaritin (**29**), isolated from *S. satureiifolia* var. *satureiifolia* and from *S. maimarensis* [1], has been reported to exert various activities including antiprotozoal, anti-inflammatory, antitumor, antioxidant, GABA modulator, antinociceptive, antidepressant and anxiolytic, diabetes treatment, and to alleviate heart failure, among others.

The trypanocidal and leishmanicidal activities of compound **29** were evaluated by Tasdemir et al. [144]. Cirsimaritin showed IC_50_ values of 3.9 µg/mL, 3.3 µg/mL, and 19.7 µg/mL for *L. donovani*, *T. brucei rhodesiense*, and *T. cruzi*, respectively. According to Quintanilla-Licea et al. [131], compound **29** also presented antiprotozoal activity against *Entamoeba histolytica* (IC_50_ = 154.26 µg/mL).

Abdelhalim et al. [145] demonstrated biphasic activity at α1β2γ2L GABA receptors by cirsimaritin. The flavonoid **29** acted as a positive modulator when applied in the presence of low concentrations of GABA but in the presence of high concentrations of GABA, it acted as a negative modulator (inhibiting currents due to 100 µM GABA by 23.0 ± 0.5% at 100 µM and positively modulating currents due to 10 µM GABA by 89.9 ± 1.5%). This activity permits cognition enhancement whilst offering protection from convulsant activity. Furthermore, Abdelhalim et al. [146] demonstrated antinociceptive, antidepressant, and anxiolytic activities of cirsimaritin mediated via GABA-A receptors. Central antinociceptive analgesic effects of cirsimaritin were determined in hot plate and tail immersion tests. Central analgesic effects maximum inhibition 74.71% was observed at 100 mg/kg, assessed by the tail immersion test. Compound **29** exerted significant antidepressant effects evidenced by the reduction of immobility time in both the tail suspension test and the forced swimming test. Cirsimaritin also exerted significant anxiolytic effects at the doses of 10–100 mg/kg in both the elevated plus maze and light dark tests used. This compound has previously been shown to have biphasic modulation of 122L GABA receptors, and has demonstrated central nervous system activity in mouse models of antinociception, antidepressant, and anxiolysis.

Cirsimaritin also displayed activity in the antigiardial bioassay with an IC_50_ = 3.8 M [147].

The effect of compound **29** on melanogenesis was investigated by Kim et al. [148]. They studied the melanin-inducing properties of cirsimaritin in murine B16F10 cells. Results indicated that cirsimaritin stimulated melanogenesis in B16F10 cells by activation of response element-binding protein (CREB) in addition to upregulation of the expression of microphthalmia-associated transcription factor (MITF) and tyrosinase expression, which was activated by cAMP signaling. Later, the melanogenic effect of cirsimaritin was confirmed in human epidermal melanocytes.

Wu et al. [149] reported that compound **29** mitigated cardiac remodeling and left ventricular dysfunction through augmenting myocardial autophagy and decreasing matrix metalloproteinase activities. This compound also affected the serum levels of Ang II, NE, TNF-α, and BNP in rats with heart failure and attenuated the cardiac histological changes.

Lee at al. [150] demonstrated the therapeutic potential of compound **29** in the prevention and treatment of type 1 diabetes mellitus. Protein expressions related to apoptosis and the effects against streptozotocin (STZ)-induced cytotoxicity in INS-1 cells were evaluated. Cirsimaritin demonstrated improved cell viability to near normal levels and protected INS-1 cells against STZ-induced damage. Furthermore, cirsimaritin reduced the intracellular oxidative stress induced by STZ. Later, with an IC_50_ value of 0.43 ± 0.07 μM, cirsimaritin was found to be a potent inhibitor of dipeptidyl peptidase IV (DPP-IV enzyme) for the management of type 2 diabetes [151].

Antimetastatic activity of compound **29** in breast cancer was shown by Yeon Park et al. [152]. They demonstrated cirsimaritin-inhibited angiogenesis through the downregulation of VEGF, p-Akt, and p-ERK in MDA-MB-231 cells using three in vitro cell-based assays: the cell proliferation assay, tube-formation assay, and Western blot analysis. Cirsimaritin inhibited the viability of HUVECs in a dose-dependent manner, achieving 62.04% at a level of 100. Cirsimaritin also reduced tube formation by 32.18% at the levels of 6.25 µM.

The anti-inflammatory effect of cirsimaritin was reported by Shin et al. [153]. This flavonoid was shown to inhibit nitric oxide (NO) production and inducible nitric oxide synthase expression in RAW264.7 cells. The compound inhibited interleukin-6, tumor necrosis factor-α, and NO production in a concentration-dependent manner in lipopolysaccharide (LPS)-stimulated RAW264.7 cells. In addition, **29** suppressed activation of LPS-induced transcription factors, such as c-fos and signal transducer and activator of transcription 3 (STAT3), in RAW264.7 cells. Therefore, cirsimaritin demonstrated anti-inflammatory activity regulated by the inhibition of c-fos and STAT3 phosphorylation in RAW264.7 cells.

Yan et al. [154], investigated the anti-influenza virus efficacy and antiviral mechanism of cirsimaritin. This compound was shown to inhibit the virus replication by downregulating the NF-κB signal transduction pathway. Manurung et al. [155] reported that this compound exhibited very strong anticancer and antioxidant activity. Pathak et al. [156] investigated the anticancer potential of cirsimaritin in organ specific cell lines by using MTT assay. The compound showed selective anticancer activity against the NCIH-520 cell line (IC_50_ = 23.29 µM), and also inhibited the proliferation of other cell lines up to 48% at 100 µM. Moreover, an increase in the ROS levels of 1.6 fold (10 µM) and 1.8 fold (100 µM) was observed; cirsimaritin also inhibits the activity of ODC and CATD with IC_50_ = 57.30 and 68.22 µM, respectively. It exhibited a good binding score with the selected targets, followed Lipinski’s rule of five, and is non-mutagenic. Hence, **29** inhibited the proliferation of lung squamous cell lines by inducing apoptosis. It also inhibited the activity of ODC and CATD responsible for the progression phase in the cancer cells.

When an anti-HIV-1 RT assay was performed, cirsimaritin displayed moderate activity with 52.50% inhibition at 200 μg/mL [157].

Hispidulin (**35**) (5,7-dihydroxy-2-(4-hydroxyphenyl)-6-methoxy-4H-chromen-4-one) isolated from *S. urticifolia* and *S. sanguinea* [1] has a wide range of reported biological activities, including antiparasitic, anti-inflammatory, antidiabetic, anticonvulsant, antiosteoporotic, antioxidant, and anticancer properties.

In vitro trypanocidal and leishmanicidal activities of the flavonoid hispidulin were reported by Sülsen et al. [117]. The IC_50_ values on *Trypanosoma cruzi* epimastigotes and trypomastigotes were 46.7 µM and 62.3 µM, respectively. Hispidulin was more active on promastigotes of *Leishmania mexicana* (IC_50_ = 6.0 µM). Years later, Grecco et al. [127] reported that hispidulin displayed moderate activity against trypomastigotes of *Trypanosoma cruzi* (Y strain), with IC_50_ values of 80.61 µg/mL.

Abdelhalim et al. [145] demonstrated that hispidulin acts as a positive modulator when applied at low concentrations of GABA but at high concentrations, it acts as a negative modulator. Moreover, hispidulin was found to act as a positive allosteric modulator at GABA-A receptor subtypes (α1-3,5,6β2γ2), being more potent at α1,2,5β2γ2 subtypes than at α3,6β2γ2. Hispidulin was also shown to have an anticonvulsant action in seizure-prone mongolian gerbils and to cross the blood-brain barrier.

The antidiabetic activity of hispidulin was shown by Abbas et al. [151]. With an IC_50_ value of 0.49 ± 0.1 μM, hispidulin was found to be a potent inhibitor of dipeptidyl peptidase-4 enzyme DPP-IV, as an effective therapeutic target for the management of diabetes mellitus.

Bourdillat et al. [158] demonstrated a correlation between the inhibition of platelet aggregation and the increase in cAMP levels induced by hispidulin. They showed that hispidulin inhibited platelet aggregation triggered by adenosine-5’-monophosphate, arachidonic acid, paf-acether, and collagen. Hispidulin (100 pM) increased the control cAMP level in platelets 4-fold.

Prolyl oligopeptidase’s inhibitory activity of hispidulin was reported by Marques et al. [159]. Inhibitory assays indicated that at a concentration of hispidulin 100 μM inhibited 43% of total prolyl oligopeptidase (POP) activity.

Mercader and Pomilio [160] performed a predictive analysis based on the quantitative structure–activity relationships (QSAR) of a property of hispidulin, which is the inhibition (IC_50_) of influenza H1N1 virus neuraminidase (IC_50_ = 13.90 µg/mL).

Yu et al. [161] evaluated the therapeutic role of hispidulin in gastric cancer through the induction of apoptosis via NAG-1 signaling. Results demonstrated that hispidulin inhibits the growth of AGS gastric cancer cells. They found that after hispidulin treatment, NAG-1 remained highly expressed, whereas COX-2 expression was downregulated. Flow cytometric analysis showed that hispidulin induces G1/S phase arrest and apoptosis in time- and concentration-dependent manners. G1/S arrest correlated with upregulated p21/WAF1 and p16 and downregulated cyclin D1 and cyclin E, independent of p53 pathway. Furthermore, hispidulin could elevate Egr-1 expression and ERK1/2 activity, whereas ERK1/2 inhibitor markedly attenuated NAG-1 mediated apoptosis.

It was reported that hispidulin showed potent in vitro cytotoxicity against human carcinoma A549, MCF-7, and HeLa cell lines [162]. Previously, it was also reported that hispidulin could inhibit the proliferation of human esophageal squamous carcinoma Eca-109, human nasopharyngeal carcinoma KB, and human colon carcinoma CL-187 cells in vitro. The in vivo inhibitory effect on sarcoma 180 (S-180) and hepatoma H22 cells in mice was evaluated by Xie et al. [163]. In addition, Reutrakul et al. [164] demonstrated in vitro cytotoxicity of hispidulin against murine lymphocytic leukemia P388, human colon carcinoma Col-2, human breast carcinoma 359 BCA-1, and human lung carcinoma Lu-1.

Hispidulin antiosteoporotic activity was investigated by Nepal et al. [165]. The authors demonstrated that hispidulin attenuates bone resorption and osteoclastogenesis via the RANKL-induced NF-κB and NFATc1 pathways. Hispidulin was found to inhibit RANKL-induced activation of Jun N-terminal kinase (JNK) and p38, in addition to the NF-κB in vitro experiment. Hispidulin also decreased NFATc1 transcriptional activity in RANKL-induced osteoclastogenesis.

Hispidulin could inhibit epithelial-mesenchymal transition (EMT), an important initiaton step in the process of metastasis, in breast cancer cells (MCF-7 and HCC38). This flavonoid also may inhibit cell migration by repressing the Smad2/3 signaling pathway [166].

Liu et al. [167] demonstrated that hispidulin affects cell proliferation, apoptosis, cell cycle, angiogenesis, and metastasis. In addition, hispidulin exhibited synergistic antitumor effects when combined with some common clinical anticancer drugs. It reduced the efflux of chemotherapeutic drugs, enhanced the chemosensitivity of cancer cells, and reversed drug resistance.

Several biological activities have been reported for 5-hydroxy-3,6,7,3’,4’-pentamethoxyflavone (artemetin) (**82**) isolated from *S. procumbens* and *S. jujuyensis* [1].

The anti-inflammatory activity of artemetin was reported by Serti’e et al. [168]. They demonstrated that artemetin showed anti-inflammatory activity using various experimental models in rats, including inhibiting carrageenan-induced paw edema, reduced granuloma formation, and reduced vascular permeability to intracutaneous histamine.

Artemetin can also protect endothelial function by acting as an antioxidant and antiapoptotic agent and through the activation of extracellular-signal-regulated kinases 1/2 (ERK1/2) and Akt. [169]. When the antioxidant activity of artemetin was determined by the DPPH (1, 1-diphenyl-2- picrylhydrazyl) radical-scavenging assay, it appeared to have no activity because its IC_50_ values exceeded 500 μM. On the other hand, it effectively inhibited the NO production in LPS-induced RAW264.7 cells, demonstrating anti-inflammatory activity [170].

This flavonoid was able to dose-dependently reduce the mean arterial pressure. Hypotensive effects induced by artemetin were attributed to its ability to decrease angiotensin II generation in vivo by ACE inhibition [171].

Artemetin exhibited antimalarial activity against *Plasmodium falciparum* (IC_50_ = 26.0 µM) [172]. In vitro trypanocidal activity against bloodstream forms of *Trypanosoma brucei rhodesiense* STIB 900 displayed an IC_50_ value of 4.7 µg/mL [173].

Wee et al. [174] reported that artemetin inhibited the production of both TNF-α and IL-1β cytokine production in human U937 macrophages. They observed that artemetin inhibited cell viability of U937 macrophages. Artemetin at 50 μg/mL and 100 μg/mL reduced TNF-α level to 20 and 30%, respectively, and at 50 μg/mL significantly reduced IL-1β levels to 60%. Ono et al. [175] reported that artemetin showed a GI_50_ of 2270 ng/mL in human lung cancer PC-12 cells and 2200 ng/mL in human colon cancer HCT116 cells. Artemetin also decreased the growth of human leukemia HL-60 cells in a dose-dependent manner, with an IC_50_ of 39.98 μM after 96 h.

The flavonoid quercetin (**37**) isolated from *S. urticifolia*, *S. pilosa* and *S. eupatoria* [1] demonstrated varied pharmacological functions including antiprotozoal, antioxidant, antibacterial, anti-inflammatory, antidiabetic, and anticancer properties.

Antiprotozoal in vitro activities of quercetin against *Trypanosoma brucei rhodesiense, Trypanosoma cruzi*, and *Leishmania donovani* were tested by Tasdemir et al. [144]. Remarkable leishmanicidal potential was observed, with an IC_50_ of 1.0 µg/mL. Furthermore, quercetin’s antimalarial and antileishmanial activity and activity against Dengue was reported by Boniface and Ferreira [176]. Quercetin revealed antimalarial activity against *P. falciparum* NF54/64, with an IC_50_ value of 5.5 μg/mL. It showed inhibitory effects against rCPB2.8 proteinase from *L. mexicana*, with an IC_50_ value of 18.03 μM. Quercetin also showed in vitro antileishmanial activity against promastigote (IC_50_ = 0.7 μM) and intracellular amastigote (IC_50_ = 4.3 μM) forms of *L. amazonensis*. In an in vivo test, quercetin (30 mg/kg) reduced the lesion size from 1.8 (vehicle) to 0.2 mm (treated) in *Leishmania amazonensis*-infected mice. Furthermore, quercetin showed arginase inhibitory effects on *L. (L.) amazonensis* with IC_50_ values of 3.8 μM. Quercetin also showed inhibitory effects (66% inhibition at 96 μM; IC_50_ = 31.4 μM) against *L. amazonensis*. Furthermore, quercetin (16 mg/kg) reduced the parasite (*L. amazonensis*) load in mice infected with cutaneous leishmaniasis. When the antileishmanial activity against promastigote forms of *L. (V.) braziliensis* was evaluated, quercetin showed IC_50_ values of 30.49 μM. The antileishmanial activity of quercetin against *L. donovani* AG83 showed an IC_50_ value of 45.5 μM for the promastigote forms and IC_50_ values of 10.50 μM for intracellular amastigotes. In addition, quercetin exhibited an IC_50_ value of 35.7 μg/mL against DENV-2 (after 96 h of incubation), reduced the DENV-2 RNA level by 67%, and proved to be active against DENV-2 NS2B-NS3 and DENV-3 NS2B-NS3.

Quercetin demonstrated antioxidant activity and superoxide anions scavenging activity. This flavonoid exhibited an IC_50_ value on superoxide anions generation of 207 µM and on lipid peroxidation of 5.2 µM [177]. Moreover, Mlcek et al. [178] highlighted the role of quercetin in relation to respiratory allergic diseases (in vitro, animal, and epidemiological studies) and food allergies.

Quercetin’s inhibition of the *Helicobacter pylori* (HpFabZ) β-hydroxyacyl-acyl dehydratase carrier protein carried out by enzymatic assay and crystal structure analysis showed an IC_50_ value of 39.3 mM [129].

Mercader and Pomilio [160] performed a predictive analysis based on the potential of the flavonoids to inhibit H1N1 virus neuraminidase. Quercetin showed an IC_50_ value of 17.65 µg/mL. The obtained model suggested that the activity depends on the electric charges, masses, and polarizabilities of the atoms present in the molecule as well as its conformation.

Li et al. [179] carried out a review of the main effects of quercetin on inflammation and immune function. Quercetin was reported as a long-lasting anti-inflammatory compound with strong anti-inflammatory capacities. Quercetin was also reported to play a modulating, biphasic, and regulatory role in inflammation and immunity, and to have an immunosuppressive effect on dendritic cells function. In in vivo animal models, quercetin also exhibited anti-inflammatory effects: it ameliorated the inflammatory response induced by carrageenan and a high-fat diet, reduced visceral adipose tissue TNF-α and nitric oxide production, and downregulated nitric oxide synthase (NOS) expression in obese Zucker rats, and decreased clinical signs of arthritis in chronic rat adjuvant induced arthritis.

Quercetin’s antihypertensive mechanism of action was reported by Marunaka et al. [180] and the antidiabetic potential mechanisms were reported by Eid and Haddad [181].

A comprehensive review of the anticancer potential of quercetin was carried out by Rauf et al. [182]. Since quercetin has exhibited direct proapoptotic effects on tumor cells, they present an overview of recent developments on the use of quercetin against different types of cancer and the probable mechanisms of action. The evidence revealed that quercetin is able to inhibit various types of cancers including breast, lung, nasopharyngeal, kidney, colorectal, prostate, pancreatic, and ovarian cancer. In addition, Shafabakhsh et al. [183] investigated the chemo-preventive and curative profile of quercetin for ovarian cancer.

The effects of quercetin in Alzheimer’s disease as a neuroprotective compound were reported by Khan et al. [184].

A large number of flavonoids have presented antiparasitic and antiviral activity and have been considered as candidates for the development of drugs for malaria, Chagas disease, leishmaniasis, and dengue [176]. Among them, the flavonoids quercetin-3-O-β-D-Gucopyranoside (**83**), isolated from *Stevia rebaudiana* and *Stevia nepetifolia*, and quercetin-3-O- β-D-Galactopyranoside (**84**), isolated from *S. nepetifolia*, *S. serrata*, and *S. soratensis*, have shown antiplasmodial and leishmanicidal activity.

Luteolin (**85**) has been identified in *S. pilosa* and *S. eupatoria* [11]. This flavonoid, together with quercetin (**37**) and glycoside flavonoids were described in *S. rebaudiana* [185]. Tasdemir et al. [144] demonstrated its leishmanicidal potential against *L. donovani* amastigotes in vitro (IC_50_ = 0.8 µg/mL). In 2018, Boniface and Ferreira [176] published a review on flavonoids as trending compounds to treat neglected tropical diseases (NTD), in which the authors describe luteolin’s antiprotozoal activity against *L. donovani*, *P. falciparum*, and *T. cruzi*, and its antiviral properties against Dengue virus type 1. Mercader et al. [160] also determined the antiviral activity of luteolin through QSAR studies on influenza H1N1 virus neuraminidase inhibition (IC_50_ = 9.65 µg/mL). This compound also showed COX-2 (Cyclooxygenase-2) inhibition through software tools and in vitro assays as demonstrated by IC_50_ values of 36.6 µmol/L [186] and xanthine oxidase inhibitory activity in vitro with IC_50_ = 1.24 µmol/L and Ki = 0.90 µmol/L [187]. Antidiabetic properties of this compound have also been reported by Abbas et al. [149]. The authors estimated IC_50_ values of luteolin and other flavonoids on different enzymes involved in glucose uptake and results showed that compound **85** had the best inhibitory activity on dipeptidyl peptidase IV (DPP-4) enzyme (IC_50_ = 0.12 µmol/L).

From *S. rebaudiana* [1], the isolation of the flavonoid apigenin-4′-O-glucoside (**86**) has been described. In 2015, Krasteva et al. [188] tested the compound on a t-BuOOH-induced oxidative stress model on rat hepatocytes. Flavonoid **86** preserved cell viability by 159% as compared to the t-BuOOH group, which can be translated to hepatoprotective and antioxidant activity.

The extraction of *S. sorantensis* resulted in apigetrin (apigenin-7-O-glucoside) (**87**) [1]. In order to investigate the anti-inflammatory effect of this compound, an immunohistochemical assay was conducted to evaluate the binding capability to TLR4/MD2 and nuclear translocation of NF-κB p65. Molecular docking data showed that apigetrin powerfully bound to MD2 and TLR4 via hydrogen bonding and could be used as immunomodulator for the effective treatment of LPS-mediated inflammatory diseases [189]. The in vitro modulation of the complement system was determined on **87** by the assay based on the hemolysis of erythrocytes membrane generated after complement activation, both for the classical way and for the alternative one. Apigenin-7-O-glucoside showed an IC_50_ of 52.5 mM, indicating remarkable anticomplementary activity.

Minda et al. [190] studied compound **87**′s anticancer properties, for which anti proliferative activity was assessed by standard MTT dye uptake assay on a HeLa human cervical cancer cell line, presenting an IC_50_ = 18.28 µM. In addition, the proapoptotic activity was tested on a HeLa human cervical cancer cell line; DAPI (4′,6-diamidino-2-phenylindole) nucleic acid staining and double Annexin V-FITC staining were performed, resulting in the phenomena of early apoptosis, late apoptosis, and a low percentage necrosis. In 2019, Villa-Rodriguez et al. [191] demonstrated that compound **87** induces acute inhibition of glucose absorption, which ultimately translates to the attenuation of cholesterol uptake. On the same subject, Jia et al. [192] conducted an in vitro experiment to determine the α-glucosidase inhibition effect of apigetrin, which presented an IC_50_ = 22.80 ± 0.24 μM. The authors also developed an in vivo assay that proved that this compound effectively improved insulin resistance and glucose uptake increased by approximately 73.06% relative to the model group of insulin-resistant HepG2 cells, concluding that this apigenin gucoside might serve as an insulin sensitizer.

The methoxylated flavonoid casticin (**88**) was obtained from *S. breviaristata* and *S. vaga* [1]. Recently, in 2020, a review on the potential antineoplastic effects of casticin was published [193]. This paper describes that the compound has been studied against cancers, including breast, bladder, oral, lung, leukemia, and hepatocellular carcinomas, and that casticin inhibits the invasion, migration, and proliferation of cancer cells and induces apoptosis (casticin-induced, ROS-mediated, and mitochondrial-dependent) and cell cycle arrest (G0/G1, G2/M, etc.) through different signaling pathways, namely the PI3K/Akt, NF-κB, STAT3, and FOXO3a/FoxM1 pathways.

Koh et al. [194] tested the anti-inflammatory and antiallergic potential of compound **88**. Casticin significantly suppressed eotaxin production in cytokine-activated A549 lung epithelial cells and also suppressed the mRNA expression levels of eotaxin, RANTES, VCAM-1, and ICAM-1, which subsequently contributed to the inhibition of eosinophil migration. Furthermore, compound **88** inhibited IkB-phosphorylation and nuclear translocation of p65 in A549 cells. These results suggest that casticin has the potential for use in the treatment of allergic asthma. The analgesic and spasmolytic potential of casticin has been studied through the years. In 1995, Bergendorff et al. [195] explored the spasmolytic activity of the compound, which showed a 53–63% relaxation of carbacholine induced contractions of guinea pig trachea. In 2007, Hu et al. studied compound **88** for its antinociceptive properties in vivo, exhibiting a significant dose-dependent reduction of analgesia in acetic acid-induced writhing in mice. This paper also proved the antihyperprolactinemia activity of casticin in treated mice [196].

Most recently, in 2011, Webster et al. [197] published their investigation in which they associated an opioidergic mechanism with the analgesic effect of casticin. Receptor binding assays and GTPgS binding assays were performed in order to elucidate if casticin acts as an agonist. Casticin was found to have the highest affinity to μ-opioid receptors with an IC_50_ of 2.84 ± 0.707 μM (Ki = 1.14 ± 0.167 μM). Casticin also stimulated [^35^S]GTPγS [Guanosine-5′-O-(3-thiotriphosphate)] binding (10 and 50 mM) and was inhibited by the opioid receptor selective antagonist ICI 174,864 (N,N-diallyl-Tyr-Aib-Aib-Phe-Leu-OH: Aib = alpha-aminoisobutyric acid) (10 mM), concluding that casticin acted as an opioid receptor agonist. The latest biological activity reported for this flavonoid is the attenuation of osteoarthritis-related cartilage degeneration [198]. An in vivo experiment in male BALB/c mice was conducted. Results indicated that the casticin treatments markedly reduced the destruction of cartilage and the levels of matrix metalloproteinase-13 (MMP13) in cartilage. Oxidative stress and inflammation of the cartilage were also decreased and proinflammatory cytokine production was suppressed.

According to Hernandez et al. [1] eupatilin (**89**) has been reported from *S. gilliesii*, *S. maimarensis*, and *S. lucida*. In 2018, [199] Nageen et al. published a review about the wide range of pharmacological properties of this compound. Anticancer activity has been extensively studied in compound **89**, including proapoptotic and cell cycle arrest activities, NF-κB/STAT3 signaling pathways suppression, and inhibition of PI3K/AKT and MAPK pathways. All of these mechanisms result in its reported activity against gastric cancer, leukemia cancer cells, renal carcinoma, hepatocellular cancerous cells, osteosarcoma cancer cells, glioma cancerous cells, and melanoma cancer cells, among others. The review also presents the anti-inflammatory properties of eupatilin against various disease models and its molecular targets, antioxidant activity through different molecular mechanisms, and neuroprotective activity both in vitro and in vivo. In 2020, Li et al. [200] conducted an in vitro experiment that showed that compound **89** suppressed the proliferation and migration of airway smooth muscle cells (ASMCs). Exposure of ASMCs to eupatilin increased the expression of contractile markers smooth muscle, whereas the expression of extracellular matrix (ECM) proteins, type I collagen (Coll I), and fibronectin were reduced; the activation of nuclear factor κB (NF-κB), signal transducer and activator of transcription 3 (STAT3), and AKT pathways caused by TGF-β1 in ASMCs was present. These findings suggest that eupatilin might attenuate airway remodeling, with great potential as an antiasthmatic.

Jeong et al. [201] investigated the in vivo effects of eupatilin on pain severity and cartilage degradation in an experimental rat model of osteoarthritis. Their results suggest that eupatilin suppresses oxidative damage and reciprocally enhances extracellular matrix production in articular chondrocytes, making the flavonoid **89** a promising therapeutic option for the treatment of osteoarthritis. Given the lipophilic nature of eupatilin and its antioxidant and anti-inflammatory activities, authors Zhang et al. [202] proposed to investigate the effects of eupatilin on a mouse model of Parkinson’s disease induced by 1-methyl-4-phenyl- 1,2,3,6-tetrahydropyridine (MPTP). The data obtained demonstrated that compound **89** alleviates behavioral impairment and dopaminergic neuron loss induced by MPTP through the inhibition of neuroinflammation and apoptosis. Potential as an antidiabetic agent has also been reported for eupatilin. In vitro α-glycosidase and α-amylase inhibition assays were carried out, affording IC_50_ values of 324.28 nM and 244.35 nM, respectively, in the presence of eupatilin [115]. On the other hand, in vivo studies were performed to investigate the dose–response effects on blood glucose regulation and pancreatic β-cell function in type 2 diabetic mice treated with eupatilin. This flavonoid significantly lowered blood glucose concentration while it increased hepatic glycogen content, and reduced hemoglobin A1c and plasma glucagon levels, along with a simultaneous increase in plasma insulin and adiponectin levels and increased pancreatic insulin concentrations [203].

In 2013, Son et al. [204] demonstrated compound **89** antiatherogenic properties in human aortic smooth muscle cells, showing that aortic sprouting as well as PDGF BB-induced proliferation and the migration of human aortic smooth muscle cells were significantly inhibited by eupatilin, which is likely mediated through the attenuation of PI3K, MKK3/6, and MKK4 factors activation. Lastly, Metoui et al. [205] assessed in vitro the antixanthine oxidase activity of **89**, showing an IC_50_ value of 3.3 µM vs. the result of allopurinol’s IC_50_ of 8.2 µM, constituting a promising compound for gout treatment.

The flavonoid chrysosplenetin (3, 6, 7, 3′-tetramethoxy quercetin) (**90**) has been found in *S. jujuyensis* [1]. Şöhretoğlu et al. tested its cytotoxic effect on the MCF-7 breast cancer cell line and determined its effect on DNA intercalation and on the activity of topoisomerases I and II. The compound inhibited the proliferation of the MCF-7 cell line with an IC_50_ value of 0.29 μM. Furthermore, it possessed dual topoisomerase I and II inhibitory properties. Especially, it inhibited topoisomerase II by 83–96% in the range 12.5–100 μM. Anti-inflammatory properties of this compound have been described by Chougouo et al. [206]. The authors tested its capacity to modulate the activity of anticholinesterase (AchE) and the production of nitric oxide (NO) in LPS-activated RAW264.7 macrophages. Compound **90** presented a high inhibitory capacity of NO production, with more than 100% inhibition relative to the control, with the respective cell viability values of 15.89%. Chrysosplenetin also exhibited 80% AchE inhibition (at 0.1 mg/mL) and afforded IC_50_ values of 27.14 µg/mL.

Given the previously reported anti-inflammatory activity of compound **90**, Ebada et al. [207], in the context of the coronavirus pandemic, proposed to study anti-inflammatory, antiallergic, and COVID-19 protease inhibitory in vitro activities of chrysosplenetin, which revealed a potent inhibitory effect on neutrophil elastase release and superoxide anion generation by human neutrophils with IC_50_ values of 6.66 µM and 4.32 µM, respectively. It also displayed potent inhibitory activity against antigen-induced degranulation with IC_50_ values of 5.8 µM. Molecular modeling was performed and chrysosplenetin was among the top compounds that showed the best docking in the active site of human elastase (1H1B), and has shown comparable binding affinity to the co-crystalized ligand in the active site of SARS-CoV-2 main protease (6LU7). Antiviral activity against enterovirus 71 (EV71) was also explored by Zhu et al. [208]. Results showed that chrysosplenetin presented strong in vitro activity against EV71 with low cytotoxicity. In the cytopathic effect (CPE) inhibition assays, both plaque reduction assay and virus yield inhibition assay, the compound showed an IC_50_ value of about 0.20 µM. On the other hand, antiprotozoal in vitro activity was studied against *T. brucei brucei* (IC_50_ = 95.5 µM) and *P. falciparum* (IC_50_ = 85.5 µM) and *T. congolense* (IC_50_ = 2.9 µM), by Ortiz et al. [209] and Nurbek et al. [210], respectively.

Evidence of the capacity of compound **90** to inhibit key molecular targets with pharmacological applications has been reported. Arroo et al. [211] proved that the compound inhibited tyrosinase in vitro (IC_50_ = 99.87 µM) and in silico as a competitive inhibitor, with potential applications in cosmetics, medicine, and agriculture. In 2019, Cao et al. [212] evaluated the neuraminidase inhibitory activity of several compounds, using a fluorescence-based assay. Flavonoid **90** showed comparable activity to oseltamivir acid on the neuraminidase inhibition. Through P-gp-over-expressing Caco-2 cells and a bidirectional transport experiment, Ma et al. [213] demonstrated that chrysosplenetin inhibits artemisinin efflux in mouse small intestine cells with upregulated expression of this membrane carrier, induced by the artemisinin treatment, proving that **90** can be useful to potentiate artemisinin and in multidrug resistance cases. The last activity reported about this compound involved its capacity to promote osteogenesis and inhibit estrogen defiency-induced osteoporosis [214]. Human-derived bone marrow stromal cells were cultured and treated with chrysosplenetin in the absence or presence of Wnt inhibitor dickkopf-related protein 1 (DKK1) or bone morphogenetic protein 2 (BMP2) antagonist. Results were consistent on the ability of chrysosplenetin to regulate the Wnt/β-catenin pathway.

Centaureidin (**91**) is another flavonoid isolated from *Stevia* spp. including *S. rebaudiana*, *S. nepetifolia*, *S. cuzcoensis*, and *S. galeopsidifolia* [1]. The antiproliferative effects of compound **91** were measured in vitro on three human cell lines (HeLa, MCF-7, and A431) with the MTT (3- (4,5-dimethylthiazol-2-yl)-2,5-diphenyltetrazolium bromide) assay. The highest activity was demonstrated by compound **91**, with IC_50_ values of 0.08 μM (HeLa), 0.13 μM (MCF-7), and 0.35 μM (A431) [215]. In other report, centaureidin has shown IC50 values of 0.11, 0.14 and 0.25 μM against HeLa, MCF-7 and HepG2 cell lines [216].

The anti-inflammatory and inmunomodulatory activities of this compound have been demonstrated through the years and by different mechanisms. In 2011, Li et al. [186] reported COX-2 inhibition in vitro with an IC_50_ = 45 µM. That same year, Jachak et al. [217] published their work proving both COX-1 (61.28%) inhibition and COX-2 (29.83%) inhibition in vitro, as well as antioxidant activity with DPPH radical scavenging and ABTS radical scavenging assays (IC_50_ of 7.07 and 10.88 μg/mL, respectively). Chang et al. [218] used IFN-γ promoter and T cells to characterize immunomodulatory compounds. Results showed that centaureidin (EC_50_ = 0.9 µg/mL), augmented IFN-γ promoter activity and that it induced the activity of NFAT and NFкB enhancers, which are located within the IFN-γ promoter. The antiviral action of centaureidin against Dengue virus 4 has been documented.

Qaddir et al. [219] developed an in silico investigation on phytochemicals from two local medicinal plants of Pakistan (compound **91** among them) against the non-structural protein 1 of Dengue Virus 4 (DENV4-NS1). Possible binding sites to this protein were estimated with great binding affinity (−6.4 kcal/mol) and inhibition activity was determined (Ki = 20.117 µM).

Lastly, centaureidin could be used as a melanin reductor and depigmenting agent, since Ito et al. [220] demonstrated that it induces significant morphological changes in normal human epidermal melanocytes and inhibits melanocyte dendrite elongation, resulting in a reduction of melanosome transfer in an in vitro melanocyte-keratinocyte co-culture system. Furthermore, in vitro binding assays were performed resulting in its capacity to activate the Rho pathway.

From the extraction of *S. jujuyensis*, the partially methoxylated flavonoid jaceosidin (**92**) was isolated [1]. Its anticancer potential was first reported in 2016 by Zater et al. [221]. Cytotoxic effects were investigated on three human cancer cell lines: A549 non-small-cell lung carcinoma (NSCLC), MCF7 breast adenocarcinoma, and U373 glioblastoma using a MTT colorimetric assay. The compound afforded IC_50_ of 32 and 40 µM on PC3 and B16-F10, respectively, not being active against MCF7. Most recently, in 2020, Şekerler [222] studied the effect of compound **92** in hepatocellular carcinoma cell lines, HepG2 and Hep3B, as well as in a normal cell line, NIH3T3. The in vitro antihepatocellular carcinoma activity of the compound was assessed by the MTT method. Jaseosidin had the highest anticancer activity among other compounds tested, with IC_50_ values of 137.66 µg/mL and 147.66 µg/mL on the HepG2 and Hep3B cell lines, respectively, indicating antiproliferative activity. In 2005, Lee et al. [223] reported that flavonoid **92** inhibited the binding between oncoprotein E6 of the human papillomavirus (HPV-16) and the p53 tumor suppressor protein, in addition to suppressing the binding between the E7 oncoprotein and the Rb tumor suppressor protein, performed in vitro. Furthermore, the compound inhibited the function of HPV-16 harboring cultured cervical cancer cells, suggesting that this compound might be used as a potential drug for the treatment of cervical cancers associated with human papillomavirus.

Compound **92** has been reported as an antioxidant agent. In vitro assays were conducted, resulting in the inhibition of the Cu^2+^-mediated LDL oxidation with IC_50_ values of 10.2 nM in the thiobarbituric acid-reactive substances (TBARS) assay, as well as the macrophage-mediated LDL oxidation. It also inhibited nuclear factor кB (NF- кB) activity and nitric oxide (NO) production, and it suppressed the expression of inducible nitric oxide synthase (iNOS) in LPS-induced RAW264.7 macrophages [224]. An in vivo experiment performed in 2020 by Park et al. [225] demonstrated that dietary supplementation of mice with **92** increased the expression and activity of Cu and Zn-SOD (copper and zinc-superoxide dismutase), confirming its antioxidant properties. In this paper, the authors used diabetic mice, demonstrating reduced fasting blood glucose levels and insulin resistance through the upregulation of the insulin receptor downstream pathways in the liver and skeletal muscles, as evidence for the antidiabetic activity of jaceosidin.

Lee et al. [226] investigated the antiallergic activity of compound **92** that potently inhibited the release of β-hexosaminidase from RBL–2H3 cells induced by the IgE–antigen complex, with IC_50_ values of 4.5 μM. At the same time, orally administered jaceosidin inhibited the passive cutaneous anaphylaxis (PCA) reaction in mice. Moreover, it suppressed the gene expressions of TNF-α and IL-4 in RBL–2H3 cells stimulated by IgE–antigen complex. In 2009, Min et al. [227] determined the anti-inflammatory effects of eupatilin and jaceosidin using carrageenan-induced inflammation in an air pouch on the back of mice and carrageenan-induced hind paw edema in rats. Inflammatory markers were measured. Flavonoid **92** blocked the carrageenan-induced increase in leukocyte numbers and protein levels in air pouch exudates, inhibited COX-2 expression and NF-кB activation, and markedly reduced TNF-α, IL-1 β, and prostaglandin E2 (PGE2) levels. Paw edema induced by carrageenan was reduced. Jaceosidin has been shown to attenuate osteoarthritic cartilage destruction by decreasing MMP3, MMP13, ADAMTS4, and ADAMTS5 expression levels in cultured chondrocytes and by suppressing the nuclear factor κB (NF-κB) signalling pathway, supporting its potential application as natural therapeutics for osteoarthritis [228].

The antibacterial activity of jaceosidin was first documented by Kumar et al. in 2016 [229]. This compound displayed mild antibacterial activities against glycopeptide-intermediate and vancomycin resistant *S. aureus* strains (GISA and VRSA, respectively) with a MIC = 128 µg/mL in both cultures. On the same subject, Allison et al. [230] demonstrated that **92** exhibited antibacterial activity against *E. coli* in liquid cultures with a MIC of 10 µM. Furthermore, the in vitro activity against the enoyl reductase enzyme (FabI) was measured using a spectrophotometric assay and completely inhibited FabI activity at a concentration of 100 μM. Lee et al. [231] reported that jaseocidin may be useful in developing angiogenic agents to promote the growth of collateral blood vessels in ischemic tissues. The compound stimulated the proliferation, migration, and tubulogenesis of endothelial cells as well as ex vivo sprouting from aorta rings, which are phenomena typically seen in angiogenesis. Studies were conducted to conclude that the compound activated vascular endothelial growth factor receptor 2 (VEGFR2, FLk-1/KDR) and different angiogenic signaling molecules. Human umbilical vascular endothelial cells cultures were used to determine that jaceosidin stimulates angiogenesis by activating the VEGFR2/FAK/PI3K/AKT/NF-kB signaling pathway.

According to Hernandez et al. [1], the flavonoid jaceidin (**93**) can be found in *S. cuzcoensis*. Allison et al. [230] demonstrated the antibacterial activity of jaceidin against *E. coli* (MIC = 50 μM). The antiprotozoal activity of cimpound **93** was evaluated by Elso et al. [62]. Jaceidin showed moderate activity against *T. cruzi* epimastigotes and *L. infantum* promastigotes (IC_50_ > 25 µM). Qaddir et al. [219] also determined potential inhibition against non-structural protein 1 from dengue virus 4 properties for jaceidin (Ki = 7.299 μM), as has been described for other compounds in this update. This is also the case in Nguyen et al. [187], who proved jaceidin to be a competitive inhibitor of xanthine oxidase (IC_50_ = 1.15 μM; Ki = 0.79 μM).

The antiplatelet activity of jaceidin was evaluated by Afifi and Aburjai [232]. This flavonoid showed weak activity when platelet aggregation was induced by collagen (IC_50_ = 254.7 ± 13.1 µg/mL).

In 2010, Aljančić et al. [233] applied the DPPH scavenging assay on compound **93** and found an EC_50_ value of 1.13 mM, showing its antioxidant potential. Recently, Elhady et al. [234] published a paper concerning jaceidin antitumor activity. Jaceidin showed in vitro cytotoxic effect in different cell lines and was evaluated on an in vivo experiment on mice, against Ehrlich’s ascites carcinoma. Compared to the control group, compound **93** decreased tumor weight 94.6%, improved the histological picture of tumor cells, lowered the levels of VEGF, and ameliorated the oxidative stress. Molecular docking and in silico studies suggested that jaceidin was a selective inhibitor of VEGF-mediated angiogenesis with excellent membrane permeability and oral bioavailability.

The flavonoid heteroside quercitrin (quercetin-3-O-ramnoside) (**94**) was isolated from *S. rebaudiana* as documented in the *Stevia* genus [1]. The biological activity of this compound has been extensively studied through the years, involving anti-inflammatory activity determined by Camuesco et al. [235], antiproliferative and proapoptotic effects by Cincin et al. [236], anti-Dengue virus 2 [237], osteoporosis attenuation [238], hair growth stimulation [239], improvement of hyperlipidemia and hepatic steatosis in ovariectomized mice [240], and inhibition of α-glicosidase [241]. Like other flavonoids discussed in this update, quercitrin was reviewed by Boniface and Ferreira et al. [176], showing antiprotozoal activity against *P. falciparum* (43.4% inhibition after 48 h), inhibitory effects on *L. amazonensis arginase* (IC_50_ = 10.0 µM), and activity against Dengue virus 2 (DENV-2 NS2B) (IC_50_ = 43.6 µM) and Dengue virus 3 (DENV-3 NS2B) (IC_50_ = 32.0 µM). Like other flavonoids, antiallergic properties have been attributed to quercitrin. Jegal et al. [242] described that quercitrin exhibited the most antiallergic activity against antigen-induced β-hexosaminidase release and IL-4 mRNA expression, which are markers of degranulation in RBL-2H3 cells.

Antioxidant and anti-inflammatory activities were documented for quercitrin by Razafin-drabazo et al. [243]. The authors assessed the antioxidant capacity using the DPPH free radical scavenging method and the ferric reducing antioxidant power (FRAP) assay with an IC_50_ of 20.35 µg/mL. In vivo carrageenan-induced mice paw edema was used to evaluate the anti-inflammatory activity, while the evaluation of the antalgic activity was carried out by the in vivo acetic acid-induced writhing test in mice, in which quercitrin was the most effective compound to decrease the number of writhes. In the same year, Oh et al. [244] published their investigation on the antithrombotic activity of quercitrin. Platelet aggregation, granule secretion, calcium mobilization, and integrin activation were used to assess the antiplatelet activity of quercitrin. An antithrombotic effect was determined in mice using ferric chloride (FeCl_3_)-induced arterial thrombus formation in vivo and thrombus formation on collagen-coated surfaces under arteriolar shear in vitro. Transection tail bleeding time was used to evaluate whether quercitrin inhibited primary hemostasis. Results were consistent with the antithrombotic activity of compound **94**.

Most recently, in 2021, Guo et al. [245] explored the effect of quercitrin in ostheoarthritis (OA). Molecular mechanisms of quercitrin on OA were studied in vitro in primary chondrocytes and SW1353 cells. In order to evaluate the in vivo effect, an anterior cruciate ligament transection (ACLT) rat model of OA was used. The damage degree of tibial subchondral bone and the protein level of MMP13, collagen II, and p110α in articular cartilage, was determined. The flavonoid encouraged cell proliferation and retarded extracellular matrix degradation by regulating MMP13 and the collagen II gene and protein expressions. An increase of the bone volume/tissue volume of tibial subchondral bone was observed. Quercitrin also enhanced the cartilage thickness and diminished Osteoarthritis Research Society International scores in osteoarthritic rats.

Nepetin (**36**), also known as eupafolin, can be found in *S. urticifolia* [27]. Several biological activities were described for this compound including antitumor activity [246,247], antiviral activity [248], tyrosinase inhibition [211], and α-glucosidase inhibition [249]. Regarding anti-inflammatory activity, Lee et al. [250] conducted an experiment involving human keratinocyte HaCaT cells that were treated with particulate matter (PM) in the presence or absence of eupafolin. Cyclooxygenase-2 (COX-2) levels were determined by Western blotting. Prostaglandin E2 (PGE2) production was evaluated by the enzyme immunoassay method. The generation of intracellular reactive oxygen species (ROS) was measured by the dichlorofluorescin (DCFH) oxidation assay, and nicotinamide adenine dinucleotide phosphate (NADPH) oxidase activity was determined by a chemiluminescence assay. For in vivo studies, COX-2 expression in the skin of BALB/c nude mice was analyzed by immunohistochemistry. Evidence showed that compound **36** inhibited PM-induced COX-2 protein and gene expression and PGE2 production in HaCaT cells. In addition, eupafolin suppressed PM-induced intracellular ROS generation, NADPH oxidase activity, MAPK (ERK, JNK, and p38) activation, and NK-κB activation. In vivo studies showed that topical treatment with eupafolin inhibited COX-2 expression in the epidermal keratinocytes of PM-treated mice. On the same line of investigation, Chen et al. [251] demonstrated that **36** suppresses IL-1β-induced IL-6, IL-8, and MCP-1 secretion and mRNA expression by repressing the activation of NF-κB and MAPKs, and can be exploited for its anti-inflammatory properties. Since eupafolin was proven to have anti-inflammatory and antiproliferative properties, some authors such as Chu et al. [198] studied this compound and demonstrated that it potently inhibited RANKL-induced osteoclast differentiation, formation, and bone resorption in vitro, and protected mice against the deleterious effects of titanium particle-induced calvarial osteolysis in vivo. Mechanistically, the inhibition of RANKL activated signaling pathways necessary for the induction of autophagy. This study demonstrated the potential therapeutic application of nepetin against osteoclast-mediated osteolytic diseases.

From *S. urticifolia*, Machado et al. [27] also isolated the flavonoid heteroside avicularin (**38**). Like other flavonoids, this compound has shown anti-inflammatory activity as was reported by Vo et al. [252], who tested avicularin in LPS-stimulated RAW264.7 macrophage cells. Compound **38** significantly inhibited LPS-induced excessive production of pro-inflammatory mediators such as nitric oxide (NO) and PGE2, and the protein levels of iNOS, COX-2 and pro-inflammatory cytokine IL-1β. Furthermore, avicularin significantly suppressed the LPS-induced degradation of IκB, which retains NF-κB in the cytoplasm, consequently inhibiting the transcription of pro-inflammatory genes. Antitumor activity against hepatocellular carcinoma was investigated by Wang et al. [253]. Huh7 cells were treated with avicularin in a concentration-dependent manner, and the cell proliferation was examined along with cell migration and invasion abilities, the activity of nuclear factor NF-κB (p65), cyclooxygenase-2 (COX-2), and peroxisome proliferator-activated receptor γ (PPar-γ) levels. The results indicated that avicularin treatment markedly decreased cell proliferation concentration-dependently in hepatocellular carcinoma, and inhibited cell migration and invasion in Huh7 cells. In addition, it was confirmed that the anticancer efficacy of avicularin in HCC was dependent on the regulation of NF-κB (p65), COX-2 and PPar-γ activities.

In 2018, Wang et al. [254] investigated the effect of avicularin on rheumatoid arthritis (RA) in vitro. Results demonstrated that avicularin may inhibit the inflammatory response, prevent cell viability and induce apoptosis in human rheumatoid arthritis synovial cells through preventing the activation of the MEK/NF-κB pathway. Shen et al. [255] reported the antidepressant activity of compound **38** in an in vivo mouse model induced by chronic unpredictable mild stress (CUM). Behavioral tests—sucrose preference test (SPT), forced swimming test (FST), and the tail suspension test (TST)—were performed. The levels of proinflammatory cytokines—interleukin-1β (IL-1β), IL-6, and tumor necrosis factor-α (TNF-α) in the hippocampi of mice were detected by enzyme-linked immunosorbent assay (ELISA). The apoptosis of hippocampal neuronal cells was determined using flow cytometry. The results showed that avicularin significantly relieved CUMS-induced depressive-like behaviors, significantly increased the sucrose preference of the mice, and shortened the immobility time in the FST and TST. They also found that **38** decreased CUMS-induced increases in the levels of IL-1α, IL-6, and TNF-α in the hippocampi of mice, and that it significantly decreased the apoptosis rate of hippocampal neuronal cells in mice, which was increased by CUMS. In 2013, Fujimori et al. [256] demonstrated that compound **38** suppressed intracellular lipid accumulation by repressing glucose uptake mediated by glucose transporter 4 (GLUT4) in mouse adipocytic 3T3-L1 cells.

Ayanin (**42**) was isolated from *S. subpubescens* var. *subpubescens* [28]. This compound has been reported to have antiasthmatic activity [257], protective cardiovascular effects [258], vasorelaxant activity [259], anti-inflammatory activity [260], and anticancer properties [261]. In order to evaluate the use of different natural compounds on the treatment of NTDs, Mahmoud et al. [262] tested ayanin for antiprotozoal activity. Results showed that ayanin had an IC_50_ of 8.2 µM on *L. donovani* amastigotes, IC_50_ = 11.2 µM on *T. b. rhodesiense*, and IC_50_ = 7.8 µM on *P. falciparum*.

Ermanin (**43**) was also isolated from *S. subpubescens* var. *subpubescens* by Perez Castorena et al. [26]. In 2006, Guerra et al. [263] demonstrated that this compound inhibited inducible nitric oxide synthase and cyclooxygenase-2 expression, indicating anti-inflammatory activity. In 2015, Castillo et al. [264] isolated ermanin and proved its antiproliferative activity on the human cancer cell lines A549 (lung), HBL-100 (breast), HeLa (cervix), SW1573 (lung), and T-47D (breast). Antiviral properties of this compound were reported in 2010 [265], displaying a 46.8% of inhibition on HIV-1 reverse transcriptase. Lastly, Filho et al. [266] evaluated compound **43** for its antiprotozoal activity, obtaining IC_50_ values of 40 µg/mL against *L. donovani* and 2.6 µg/mL against *P. falciparum*.

**Table 4:** Flavonoids isolated from *Stevia* species with biological activity reported.

Comp. N°	Common Name	Species	Reported Activity
28	Eupatorin	*S. satureiifolia* var. *satureiifolia*, *S. breviaristata*, *S. procumbens*, *S. vaga*	Anti-*T. cruzi* and Anti-*L. braziliensis*. Antibreast cancer. Antidiabetic. Angiotensin-converting enzyme inhibition. Antitumor. Vasorelaxant. Anti-*M. tuberculosis* [25,110,111,112,113,114,115].
29	Cirsimaritin	*S. satureiifolia* var. *satureiifolia*, *S. maimarensis*	Trypanocidal and leishmanicidal. Antioxidant activity. Anti-*E. hystolica*. Antiproliferative and antimetastatic. Anti-HIV. Antidiabetic. Anti-influenza A virus. Anti-inflammatory. Cardiac remodeling and ventricular dysfunction improvement. Antidepressant. Anxiolytic. Antinociceptive. Antiepileptic. Antigiardial activity. Melanogenesis-inducing effect [131,144,145,146,147,148,149,150,151,152,153,154,155,156,157].
35	Hispidulin	*S. urticifolia*, *S. sanguinea*	Anticancer. Anti-*T. cruzi.* Antidiabetic. Antiepileptic. Antihypnotic. Anti-influenza. Antiosteoporotic. Platelet aggregation inhibition. Antimetastatic (breast cancer cells) [117,127,151,158,159,160,161,162,163,164,165,166,167].
36	Nepetin	*S. urticifolia*	Alpha-glucosidase inhibition. Osteoclastogenesis inhibition. Tyrosinase inhibition. Anti-inflammatory. Antiangiogenic and antitumor. Antiviral [198,211,246,247,248,249,250,251].
37	Quercetin	*S. urticifolia*, *S. pilosa*, *S. aupatoria* *S. rebaudiana*	Leishmanicidal. Antimalarial. Antioxidant. Anti-influenza: neuraminidase inhibitor. Anti-*H. pylori.* Anti-inflammatory. Antiallergic. Antihypertensive. Antidiabetic. Neuroprotective. Antitumor [129,144,160,176,177,178,179,180,181,182,183,184].
38	Avicularin	*S. urticifolia*	Anti-inflammatory. Anticancer. Rheumatoid arthritis protector. Antidepressive. Adipogenic genes expression inhibitor [252,253,254,255,256].
40	Sakuranetin	*S. subpubescens* var. *subpubescens*	Anti-*T. cruzi* and anti-*L. braziliensis*. Anti-*E. hystolitica*. Antiproliferative. Antifungal. Anti- *H. pylori*. Anti-influenza B. Antiviral. Anti-inflammatory. Antioxidant. Antiasthmatic. Alzheimer’s disease treatment. Cytotoxic against melanoma cells [13,122,123,124,125,126,127,128,129,130,131,132,133,134,135,136,137,138,139,140,141].
42	Ayanin	*S. subpubescens* var. *subpubescens*	Antiasthmatic. Cardiovascular protector. Vasorelaxant. Antiallergic. Anti-inflammatory. Anticancer. Anti-*L. donovani*, -*P. falciparum*, and -*T. b. rhodesiense* [257,258,259,260,261,262].
43	Ermanin	*S. subpubescens* var. *subpubescens*	Anti-inflammatory. Antitumor. Antiviral. Anti-*L. donovani* and -*P. falciparum* [263,264,265,266]
80	Santin	*Stevia microchaeta*, *S. monardifolia*, *S. origanoides*	Anti-*T. cruzi*. Anti- *L. braziliensis.* Antiplasmodial. Antibacterial. Anti-influenza A. Anti-*T. brucei gambiense* [116,117,118,119,120,121].
81	Pectolinaringenin	*S. lucida*	Anti-*T. cruzi*. Larvicidal against *A. aegypti* and *Culex quinquefasciatus* [142,143].
82	Artemetin	*S. procumbens* *S. jujuyensis*	Anti-inflammatory activity. Antimalarial. Antioxidant. Antiapoptotic. Endothelial function protection. Anti *T. brucei rhodisiense*. Hypotensive. Antitumor [168,169,170,171,172,173,174,175].
83	Quercetin-3-O-B-D-Glc	*S. rebaudiana* *S. nepetifolia*	Anti-plasmodium and anti-*L. donovani* [176].
84	Quercetin-3-O-B-D-Gal	*S. nepetifolia*, *S. serrata*, *S. soratensis*	Anti-*L. donovani* [176].
85	Luteolin	*S. pilosa* *S. eupatoria* *S. rebaudiana*	Antiprotozoal against *P. falciparum, L. donovani*, and *T. cruzi*. Antiviral against Dengue virus type 1. Anti-influenza. Antidiabetic. Antiinflammatory. Xanthine oxidase inhibition [144,149,160,176,186,187].
86	Apigenin-4′-*O*-glucoside	*S. rebaudiana*	Antioxidant [188].
87	Apigetrin	*S. soratensis*	Anti-inflammatory. Anticomplementary. Antiproliferative and proapoptotic. Alpha-glucosidase inhibitor. Reduction of intestinal cholesterol uptake [189,190,191,192].
88	Casticin	*S. breviaristata* *S. vaga*	Anticancer. Antiasthmatic. Antihyperprolactinemia and antinociceptive. Analgesic. Spasmolytic. Osteoarthritis-related cartilage degeneration attenuation [193,194,195,196,197,198].
89	Eupatilin	*S. gilliesii* *S. maimarensis* *S. lucida*	Anticancer. Anti-inflammatory. Antiasthmatic. Antinociceptive. Chondroprotective properties. Antioxidant. Neuroprotective. Antidiabetic. Antiatherogenic. Antixanthine oxidase activity [115,199,200,201,202,203,204,205].
90	Chrysosplenetin	*S. jujuyensis*	Cytotoxic activity. Anti-inflammatory. Antiacetylcholinesterase. Antiallergic. Antiviral against SARS-CoV-2 (COVID-19) and enterovirus 71. Antiprotozoal against *T. brucei brucei*, *P. falciparum*, and *T. congolense*. Tyrosinase inhibition. Neuraminidase inhibition. Pg-P inhibition. Osteogenesis activation [206,207,208,209,210,211,212,213,214].
91	Centaureidin	*S. rebaudiana* *S. nepetifolia* *S. cuzcoensis* *S. galeopsidifolia*	Cytotoxic. Tumor cell growth inhibition. Anti-inflammatory. Antioxidant. Immunomodulatory. Anti-Dengue virus 4. Melanin pigmentation reduction [186,215,216,217,218,219,220].
92	Jaceosidin	*S. jujuyensis*	Anticancer. Antioxidant. Antidiabetic. Antiallergic. Anti-inflammatory. Osteoarthritic cartilage damage attenuation. Antibacterial. Angiogenesis stimulation [221,222,223,224,225,226,227,228,229,230,231].
93	Jaceidin	*S. cuzcoensis*	Anti-*T. cruzi*. Anti-*L. infantum*. Anti-*E. coli* activity. Anti-Dengue virus 4. Antitumor. Antiplatelet. Antioxidant activity. Xanthine oxidase inhibition [187,230,232,233,234].
94	Quercitrin	*S. rebaudiana*	Oteoarthritis alleviation. Platelet activation inhibition. Antioxidant and anti-inflammatory. Hyperlipidemia. Hepatic steatosis amelioration. Hair growth stimulation. Alpha-glycosidase inhibition. Antiallergic. Antimalarial. Antileishmanial. Anti-Dengue virus. Osteoporosis attenuation. Anticancer [176,235,236,237,238,239,240,241,242,243,244,245].

## 5. Final Remarks and Conclusions

In this review, the ethnobotanical aspects and the phytochemistry of the genus *Stevia*, as well as the biological activities reported for extracts and isolated compounds, have been summarized.

So far, data of the *Stevia* genus have shown how promising these species are as sources of natural and bioactive compounds. Ethnobotanical uses of many *Stevia* species have been part of folk medicine and popular knowledge for centuries. Of the 29 *Stevia* species that have been used in traditional medicine, only seven appear to have undergone biological evaluation of the extracts or the isolated compounds that could be related to their medicinal use.

In this sense, it was described how decoctions from *S. lucida* were used to relieve pains, treat rheumatism, and heal wounds. Isoalantolactone, a guaianolide-type sesquitepene lactone with demonstrated anti-inflammatory and antimicrobial activity has been isolated from this species. Additionally, the flavonoid eupatilin has been tested and associated with anti-inflammatory, antioxidant, antinociceptive, and chondroprotecive mechanisms. *Stevia salicifolia* was used for rheumatism and the labdanolic acid obtained from this species has been proven to have anti-inflammatory properties. Similarly, poultices prepared using *S. pilosa* were traditionally used to heal open wounds. The flavonoids quercetin and luteolin, which have shown anti-inflammatory activity, have also been reported in this species. It was reported that *S. subpubescens* has been popularly used to treat joint pains, among other uses. Extracts of this species, diterpenoid labdanolic acid and the flavonoids sakuranetin, ayanin, and ermanin have shown anti-inflamatory activity. *Stevia eupatoria* has been employed for treating paludism and as a hypoglycemiant and anti-inflammatory. These medicinal uses are consistent with the isolation of the flavonoid luteolin from this species, which has been active against *P. falciparum* and has demonstrated antidiabetic and anti-inflammatory effects.

*Stevia* extracts have shown biological activities consistent with ethnobothanical uses. For example, *S. serrata* poultices were shown to treat open wounds, snake bites, and cuts on feet. The essential oil extracted from this species has shown in vivo antinociceptive activity.

The pharmacological properties of *S. rebaudiana* extracts, as well as of its glycosylated diterpenoids, are related to the traditional uses of this species, employed as a sweetener and to decrease blood glucose levels.

In the last 22 years, only 14 *Stevia* species were studied regarding chemical composition. From these 14 species, 58 compounds have been isolated, sesquiterpene lactones, flavonoids, and longipinanes being the most frequently reported.

Many pharmacological properties have been described and documented for *Stevia* extracts and compounds isolated from them. The antiparasitic, anti-inflammatory, and antitumor potential for many of the active compounds has been reported.

Sesquiterpene lactones and flavonoids stand out for their broad range of activities reported. Although a great number of active compounds have been reported, many of them have been tested only in vitro assays. In vivo experiments, toxicological research, and studies to find out the mechanism of action of the active molecules should be conducted. In addition, further *Stevia* species should be investigated in order to contribute to the knowledge of the chemistry of the genus, as well as to discover other pharmacological properties and bioactive molecules.

This review gives the scientific background of popular and ancient knowledge about the genus *Stevia*, as well as its chemical composition and the pharmacological potential of extracts and isolated compounds. The extensive information collected highlights the diversity and richness of the *Stevia* genus and how useful its exploitation can be for the detection of new bioactive compounds.

## Figures and Tables

**Table 1:** Ethnobotanical uses of *Stevia* species.

Species	Common Name	Ethnobotanical Use	Location	Refs.
*S. achalensis*	Comadre	Ornamental.	Argentina	[8,9]
*S. balansae* Hieron.	-	Antidiarrheal.	Paraguay	[10]
*S. bogotensis* Tr. ex Cortés	Jarilla, Clavito, eupatoria	Febrifugue. Diaphoretic.	Colombia	[6,10]
*S. cardiatica* Perkins	-	Heart diseases.	Bolivia	[6,10]
*S. collina* Gardn.	Caá-ehé	Sweetener. As stomachic.	Brazil	[6]
*S. connata* Lag.	Pericón de monte	Stomachache treatment.	Guatemala	[6,10]
*S. elatior* HBK.	A-cí	To soothe burns and scratches	Mexico	[6,10]
*S. eupatoria* (Spreng.) Wild	Hierba del borrego, yerba del borrego, cola del borrego, estevia	Diuretic. Antimalarial. For stomachache. Hypoglycemiant. Analgesic. Anti-inflammatory. Antihypertensive.	Cuba	[6,10,11]
*S. fiebrigii* Hieron. var.* vattuonei* (Hicken) Cabrera	-	Ornamental.	Argentina	[8]
*S. glandulosa* Hook. et Arn.	Hierba de la pulga	Antipyretic.	Mexico	[6,10]
*S. linoides* Sch. Bip.	-	Astringent.	-	[12]
*S. lucida* Lag.	Yerba del aire, hierba de la araña, ma-li-too, kebuj, mariposa, chirca, chilca, javillo, golondrina de la sabanera	To cure wounds. To soothe pains. Rheumatism treatment. Anti-inflammatory.	Mexico, Guatemala, Colombia, Venezuela	[6,10]
*S. macbridei* B. L. Robins var. *anomala* B. L. Robins	Jauja-huancayo	Used as a bath by women.	Peru	[6,10]
*S. mercedensis* Hieron. var. *mercedensis*	Comadre	Ornamental.	Bolivia, Argentina	[9,13]
*S. nepetifolia* HBK	Zazal, anis de ratón, peracón	Dysmenorrhea treatment.	Mexico, Guatemala	[6,10]
*S. palmeri* Gray	Raniweri, raniwori	Odoriferous.	Mexico	[6]
*S. petiolata* (Cass) Sch. Bip.	Guarme-guarmi	To give flavor to meat.	Peru	[6,10]
*S. pilosa* Lag.	Flor de María	Antimalarial. Antipyretic. Cathartic. Diuretic.	Mexico	[6,10]
*S. plummerae* Gray	Ronino	To make washes and poultices for open wounds.	Mexico	[6]
*S. puberula* Hook.	Lima-lima	Used as tea substitute and stomach medicine.	Peru	[6,10]
*S. rebaudiana* Bertoni	Hierba dulce del Paraguay, estevia, stevia	Sweetener. Food additive. Contraceptive. Antidiabetic. Used to regulate arterial pressure.	Paraguay, Brazil	[6,10]
*S. rhombifolia* HKB var. *stepphanocoma* Sch. Bip.	Manka pak’I, pirq’a	Stomachache treatment. As an emetic. Used for mate.	Peru	[6]
*S. salicifolia* Cav.	Hierba del aire, hierba de la mula, la envidia, zazale de olor, yerba de la mula. Hierba de la Santa Rita	Rheumatism treatment. Cathartic. For intestinal upset due to parasites. Purgative. For fevers and colds.	Mexico, USA	[6,10]
*S. sanguinea* Hieron.	Malvisco	Ornamental.	Argentina	[8]
*S. satureiifolia* (Lam.) Sch. Bip. *ex* Klotzsch var. *satureiifolia*	Romerillo	Ornamental.	Argentina, Brazil, Uruguay	[8]
*S. serrata* Cav.	Ronino, Uriki, Otoninawa, Chapo, yerba picante, hipericón, Q’ang’aj, anis silvestre, hipericon arrie	To make washes and poultices for open wounds. Applied to cuts on feet and on snake bites. As cough remedy. For gastrointestinal disorders.	Guatemala, Mexico	[6,10,14]
*S. subpubescens* Lag.	Hierba de la mula, Zazal	As a bath after parturition. Stomachache treatment. To treat joint pains.	Mexico	[6,10]
*S. trifida* Lag.	Manzanilla de agua	Dysentery treatment.	Mexico	[6,10]
*S. yalae* Cabrera	-	Ornamental.	Argentina	[8]

**Table 3:** Diterpenes isolated from *Stevia* species with biological activity reported.

Comp. N°	Common Name	Species	Reported Activity
**46**	Labdanolic acid	*S. salicifolia*, *S. subpubescens* var. *subpubescens*	Anti-inflammatory [97].
**75**	Austroinulin	*S. rebaudiana*	Anti-inflammatory [98,99].
**76**	Kaurenic acid/kaurenoic acid	*S. monardaefolia* *S. setifera*	Anti-*T. cruzi.* Anti-*Leishmania.* Antimalarial. Antioxidant. Antitumor. Anti-inflammatory. Antipyretic [100,101,102,103,104,105].
**77**	Manoyl oxide	*S. berlandieri*	Radical scavenger. Inhibits PgE2 production [106,107].
**78**	Epi-manoyl oxide	*S. salicifolia*	Cytotoxic [108].
**79**	Paniculoside IV	*S. paniculata*	Alpha-glucosidase activator [109].

## Supplementary Materials

The following are available online, Table S1: Compounds isolated from *Stevia* species since 1998.

## References

[1] Hernández L.R., Catalán C.A.N., Joseph-Natan P. (1998). The chemistry of the genus *Stevia* (Asteraceae). Rev. Acad. Colomb. Ciencias.

[2] Rodríguez-Cravero J.F., Gutiérrez D.G., Katinas L., Grossi M.A., Bonifacino J.M., Marchesi E. (2019). A revision and morphological analysis of the Uruguayan species of *Stevia* (Compositae, Eupatorieae). Rodriguésia.

[3] Soejarto D.D., Kinghorn A.D. (2002). Botany of *Stevia* and *Stevia rebaudiana*. Stevia. The Genus Stevia.

[4] King R.M., Robinson H., King R.M., Robinson H. (1987). The Genera of the Eupatorieae (Asteraceae).

[5] Ruiz-Ruiz J.C., Moguel-Ordoñez Y.B., Segura-Campos M.R. (2017). Biological activity of *Stevia rebaudiana* Bertoni and their relationship to health. Crit. Rev. Food Sci. Nutr..

[6] Soejarto D.D., Kinghorn A.D. (2002). Ethnobotany of *Stevia* and *Stevia rebaudiana*. Stevia. The Genus Stevia.

[7] Cerda-García-Rojas C.M., Pereda-Miranda R., Kinghorn A.D. (2002). The phytochemistry of *Stevia*: A general survey. Stevia. The Genus Stevia.

[8] Rodríguez-Cravero J., Gutiérrez D., Hurrell J.A., Bayón N.D., Delucchi G. (2017). Stevia Cav. Plantas Cultivadas de la Argentina: Asteráceas-Compuestas.

[9] Cantero J.J., Núñez C.O., Bernardello G., Amuchastegui A., Mulko J., Brandolin P., Palchetti M.V., Iparraguirre J., Virginil N., y Ariza Espinar L. (2019). Las Plantas de Importancia Económica en Argentina.

[10] Soejarto D.D., Compadre C.M., Kinghorn A.D. (1983). Ethnobotanical notes on *Stevia*. Bot. Mus. Leafl. Harv. Univ..

[11] Cariño-Cortés R., Hernández-Ceruelos A., Torres-Valencia J.M., González-Avila M., Arriaga-Alba M., Madrigal-Bujaidar E. (2007). Antimutagenicity of *Stevia pilosa* and *Stevia eupatoria* evaluated with the Ames test. Toxicol. Vitro.

[12] Perez-Perez I., Valencia J.M.T. (2016). Metabolitos secundarios aislados de las raíces y las hojas de *Stevia jorullensis* H.B.K. Bachelor’s Thesis.

[13] Brown A.E., Moritán M.G., Ventura B., Hilgert N.I., Malizia L.R., Brown A.E., Moritán M.G., Ventura B., Hilgert N.I., Malizia L.R. (2007). Plantas silvestres, ámbito doméstico y subsistencia. Finca San Andrés. Un Espacio de Cambios Ambientales y Sociales en el Alto Bermejo.

[14] Cordeiro M.S., Simas D.L.R., Pérez-Sabino J.F., Mérida-Reyes M.S., Muñoz-Wug M.A., Oliva-Hernández B.E., Da Silva A.J.R., Fernandes P.D., Giorno T.B.S. (2020). Characterization of the antinociceptive activity from *Stevia serrata Cav*. Biomedicines.

[15] Sülsen V., Martino V., Sülsen V., Martino V. (2018). Overview. Sesquiterpene Lactones. Advances in Their Chemistry and Biological Aspects.

[16] Sülsen V., Elso O., Borgo J., Laurella L.C., Catalan C.A.N., Atta-ur-Rahman (2021). Recent patents involving sesquiterpene lactones with therapeutic application. Studies in Natural Product Chemistry (Bioactive Natural Products).

[17] Román L.U., Morales N.R., Hernandez J.D., Cerda-Garcia-Rojas C.M., Zepeda L.G., Flores Sandoval C.A., Joseph-Nathan P. (2001). Generation of the new quirogane skeleton by a vinylogous retro-Michael type rearrangement of longipinene derivatives. Tetrahedron.

[18] Sánchez-Arreola E., Cerda-García-Rojas C.M., Román L.U., Hernández J.D., Joseph-Nathan P. (2000). Longipinane Derivatives from Stevia connata. J. Nat. Prod..

[19] Román L.U., Cambrón J.I., Del Río R.E., Hernández J.D., Cerda-García-Rojas C.M., Joseph-Nathan P. (2000). Grindelane diterpenoids from *Stevia subpubescens*. J. Nat. Prod..

[20] Román L.U., Guerra-Ramírez D., Morán G., Martínez I., Hernández J.D., Cerda-García-Rojas C.M., Torres-Valencia J.M., Joseph-Nathan P. (2004). First seco-C Oleananes from Nature. Org. Lett..

[21] Álvarez-García R., Torres-Valencia J.M., Román L.U., Hernández J.D., Cerda-Garciía-Rojas C.M., Joseph-Nathan P. (2005). Absolute configuration of the α-methylbutyryl residue in longipinene derivatives from Stevia pilosa. Phytochemistry.

[22] Rojas-Pérez R.E., Cedillo-Portugal E., Joseph-Nathan P., Burgueño-Tapia E. (2009). A New Longipinene Diester from *Stevia monardifolia* Kunth. Nat. Prod. Commun..

[23] Valdez-Calderón A., Torres-Valencia J.M., Manríquez-Torres J.J., Velázquez-Jiménez R., Román-Marín L.U., Hernández-Hernández J.D., Cerda-García-Rojas C.M., Joseph-Nathan P. (2013). An unusual diepoxyguaianolide from *Stevia tomentosa*. Tetrahedron Lett..

[24] Ceunen S., Wim D.B., Compernolle F., Mai A.H., Geuns J.M.C. (2013). Diterpene glycosides from *Stevia phlebophylla* A. Gray. Carbohydr. Res..

[25] Beer M.F., Frank F.M., Elso O.G., Ernesto Bivona A., Cerny N., Giberti G., Malchiodi E.L., Martino V.S., Alonso M.R., Sülsen V.P. (2016). Trypanocidal and leishmanicidal activities of flavonoids isolated from *Stevia satureiifolia var. satureiifolia*. Pharm. Biol..

[26] Simas D.L.R., Mérida-Reyes M.S., Muñoz-Wug M.A., Cordeiro M.S., Giorno T.B.S., Taracena E.A., Oliva-Hernández B.E., Martínez-Arévalo J.V., Fernandes P.D., Pérez-Sabino J.F. (2019). Chemical composition and evaluation of antinociceptive activity of the essential oil of *Stevia serrata Cav.* from Guatemala. Nat. Prod. Res..

[27] Machado K.N., Tasco A.J.H., Salvador M.J., Rodrigues I.V., Pessoa C., Sousa I.J.O., Ferreira P.M.P., do Nascimento A.M. (2017). Flavonoids, Antioxidant, and Antiproliferative Activities of *Stevia urticifolia*. Chem. Nat. Compd..

[28] Perez-Castorena A.L., Arciniegas A., Nieto-Camacho A., Villasenor J.L., de Vivar A.R. (2019). Chemical Constituents of *Stevia subpubescens var. subpubescens* and Evaluation of the Anti-Inflammatory Activity. Chem. Nat. Compd..

[29] Pérez-Castorena A.L., Nieto-Camacho A., Maldonado E. (2020). Sesquiterpene lactones and other constituents from *Stevia jorullensis*. Biochem. Syst. Ecol..

[30] Chacón-Morales P.A., Dugarte C.S., Amaro-Luis J.M. (2020). Helenin from *Stevia lucida*. The first report of this natural eudesmanolide mixture in Eupatorieae tribe. Nat. Prod. Res..

[31] Fournet A., Barrios A.A., Muñoz V. (1994). Leishmanicidal and trypanocidal activities of Bolivian medicinal plants. J. Ethnopharmacol..

[32] Kedik S.A., Yartsev E.I., Stanishevskaya I.E. (2009). Antiviral activity of dried extract of *Stevia*. Pharm. Chem. J..

[33] Shukla S., Mehta A., Mehta P., Bajpai V.K. (2012). Antioxidant ability and total phenolic content of aqueous leaf extract of *Stevia rebaudiana Bert*. Exp. Toxicol. Pathol..

[34] Moselhy S.S., Ghonieim M.A., Khan J.A. (2016). In vitro and in vivo evaluation of antimicrobial and antioxidant potential of *stevia* extract. Afr. J. Tradit. Complement. Altern. Med..

[35] Medina-Medrano J.R., Torres-Contreras J.E., Valiente-Banuet J.I., Mares-Quiñones M.D., Vázquez-Sánchez M., Álvarez-Bernal D. (2019). Effect of the solid–liquid extraction solvent on the phenolic content and antioxidant activity of three species of *Stevia* leaves. Sep. Sci. Technol..

[36] Farhat G., Berset V., Moore L. (2019). Effects of *stevia* extract on postprandial glucose response, satiety and energy intake: A three-arm crossover trial. Nutrients.

[37] Martínez-Rojo E., Cariño-Cortés R., Berumen L.C., García-Alcocer G., Escobar-Cabrera J. (2020). *Stevia eupatoria* and *Stevia pilosa* extracts inhibit the proliferation and migration of prostate cancer cells. Medicina.

[38] Gonzales M., Villena G.K., Kitazono A.A. (2021). Evaluation of the antioxidant activities of aqueous extracts from seven wild plants from the Andes using an in vivo yeast assay. Results Chem..

[39] Ríos V.E., León A., Chávez M.I., Torres Y., Ramírez-Apan M.T., Toscano R.A., Bravo-Monzón Á.E., Espinosa-García F.J., Delgado G. (2014). Sesquiterpene lactones from *Mikania micrantha* and *Mikania cordifolia* and their cytotoxic and anti-inflammatory evaluation. Fitoterapia.

[40] Máñez S., Hernández V., Giner R.M., Ríos J.L., del Carmen Recio M. (2007). Inhibition of pro-inflammatory enzymes by inuviscolide, a sesquiterpene lactone from *Inula viscosa*. Fitoterapia.

[41] Moujir L., Callies O., Sousa P.M.C., Sharopov F., Seca A.M.L. (2020). Applications of sesquiterpene lactones: A review of some potential success cases. Appl. Sci..

[42] Rozenblat S., Grossman S., Bergman M., Gottlieb H., Cohen Y., Dovrat S. (2008). Induction of G2/M arrest and apoptosis by sesquiterpene lactones in human melanoma cell lines. Biochem. Pharmacol..

[43] Luna-Herrera J., Costa M.C., González H.G., Rodrigues A.I., Castilho P.C. (2007). Synergistic antimycobacterial activities of sesquiterpene lactones from *Laurus Spp*. J. Antimicrob. Chemother..

[44] Asaruddin M.R., Honda G., Tsubouchi A., Nakajima-Shimada J., Aoki T., Kiuchi F. (2003). Trypanocidal constituents from *Michelia alba*. J. Nat. Med..

[45] Sánchez L.A., Capitan Z., Romero L.I., Ortega-Barría E., Gerwick W.H., Cubilla-Rios L. (2007). Bio-Assay Guided Isolation of Germacranes with Anti-Protozoan Activity from *Magnolia sororum*. Nat. Prod. Commun..

[46] Julianti T., Hata Y., Zimmermann S., Kaiser M., Hamburger M., Adams M. (2011). Antitrypanosomal sesquiterpene lactones from *Saussurea costus*. Fitoterapia.

[47] Lee B.K., Park S.J., Nam S.Y., Kang S., Hwang J., Lee S.J., Im D.S. (2018). Anti-allergic effects of sesquiterpene lactones from *Saussurea costus* (Falc.) *Lipsch*. determined using in vivo and in vitro experiments. J. Ethnopharmacol..

[48] Eliza J., Daisy P., Ignacimuthu S. (2010). Antioxidant activity of costunolide and eremanthin isolated from *Costus speciosus* (Koen ex. Retz) Sm. Chem. Biol. Interact..

[49] Lee Y.S., Choi E.M. (2011). Costunolide stimulates the function of osteoblastic MC3T3-E1 cells. Int. Immunopharmacol..

[50] Ham A., Lee S.J., Shin J., Kim K.H., Mar W. (2012). Regulatory effects of costunolide on dopamine metabolism-associated genes inhibit dopamine-induced apoptosis in human dopaminergic SH-SY5Y cells. Neurosci. Lett..

[51] Hajdú Z., Zupkó I., Réthy B., Forgo P., Hohmann J. (2010). Bioactivity-guided isolation of cytotoxic sesquiterpenes and flavonoids from anthemis ruthenica. Planta Med..

[52] Fischedick J.T., Standiford M., Johnson D.A., De Vos R.H., Todorović S., Banjanac T., Verpoorte R., Johnson J.A. (2012). Activation of antioxidant response element in mouse primary cortical cultures with sesquiterpene lactones isolated from *Tanacetum parthenium*. Planta Med..

[53] Fabian L., Sülsen V., Frank F., Cazorla S., Malchiodi E., Martino V. (2013). In silico study of structural and geometrical requirements of natural sesquiterpene lactones with trypanocidal activity. Mini Rev. Med. Chem..

[54] Elso O.G., Bivona A.E., Alberti A.S., Cerny N., Fabian L., Morales C., Catalán C.A.N., Malchiodi E.L., Cazorla S.I., Sülsen V.P. (2020). Trypanocidal activity of four sesquiterpene lactones isolated from Asteraceae species. Molecules.

[55] Kimani N.M., Matasyoh J.C., Kaiser M., Brun R., Schmidt T.J. (2018). Antiprotozoal Sesquiterpene Lactones and Other Constituents from *Tarchonanthus camphoratus* and *Schkuhria pinnata*. J. Nat. Prod..

[56] Michalak B., Piwowarski J.P., Granica S., Waltenberger B., Atanasov A.G., Khan S.Y., Breuss J.M., Uhrin P., Zyzynska-Granica B., Stojakowska A. (2019). Eupatoriopicrin inhibits pro-inflammatory functions of neutrophils via suppression of il-8 and tnf-Alpha production and p38 and erk 1/2 map kinases. J. Nat. Prod..

[57] Bachelier A., Mayer R., Klein C.D. (2006). Sesquiterpene lactones are potent and irreversible inhibitors of the antibacterial target enzyme MurA. Bioorganic Med. Chem. Lett..

[58] Rucker G., Heiden K., Schenkel E. (2001). Antitumor-active lactones from *Kaunia rufescens* and *Eupatorium cannabinum*. J. Indian Inst. Sci..

[59] Beekman A.C., Woerdenbag H.J., Harm H., Kampinga H.H., Antonius W.T., Konings A.W.T. (1996). Cytotoxicity of Artemisinin, a Dimer of Dihydroartemisinin, Artemisitene and Eupatoriopicrin as Evaluated by the MTT and Clonogenic Assay. Phytother. Res..

[60] Wu X.D., Ding L.F., Tu W.C., Yang H., Su J., Peng L.Y., Li Y., Zhao Q.S. (2016). Bioactive sesquiterpenoids from the flowers of Inula japonica. Phytochemistry.

[61] Boldbaatar A., Lee S., Han S., Jeong A.L., Ka H.I., Buyanravjikh S., Lee J.H., Lim J.S., Lee M.S., Yang Y. (2017). Eupatolide inhibits the TGF-β1-induced migration of breast cancer cells via downregulation of SMAD3 phosphorylation and transcriptional repression of ALK5. Oncol. Lett..

[62] Elso O.G., Clavin M., Hernandez N., Sgarlata T., Bach H., Catalan C.A.N., Aguilera E., Alvarez G., Sülsen V.P. (2021). Antiprotozoal Compounds from Urolepis hecatantha (Asteraceae). Evid. Based Complement Alternat. Med..

[63] Kimani S., Backhaus J., Matasyoh J.C., Kaiser M., Herrmann F.C., Schmidt T.J., Langer K. (2019). Preparation of sesquiterpene lactone-loaded PLA nanoparticles and evaluation of their antitrypanosomal activity. Molecules.

[64] Woerdenbag H., Hendriks H.J., Malingr’e T.M., Van Stralen R., Van den Berg K.J., Konings A.W.T. (1988). In vitro cytotoxicity of sesquiterpene lactones from *Eupatorium cannabinum* L. and semi-synthetic derivatives from eupatoriopicrin. Phytother. Res..

[65] Kudumela R.G., Mazimba O., Masoko P. (2019). Isolation and characterisation of sesquiterpene lactones from *Schkuhria pinnata* and their antibacterial and anti-inflammatory activities. S. Afr. J. Bot..

[66] Zhu Z., Yuan J., Xu X., Wei Y., Yang B., Zhao H. (2021). Eucannabinolide, a novel sesquiterpene lactone, suppresses the growth, metastasis and BCSCS-like traits of TNBC via inactivation of STAT3. Neoplasia.

[67] Liu S.J., Liao Z.X., Tang Z.S., Cui C.L., Liu H.B., Liang Y.N., Zhang Y., Shi H.X., Liu Y.R. (2017). Phytochemicals and biological activities of *Artemisia sieversiana*. Phytochem. Rev..

[68] Sanchez-Carranza J.N., González-Maya L., Razo-Hernández R.S., Salas-Vidal E., Nolasco-Quintana N.Y., Clemente-Soto A.F., García-Arizmendi L., Sánchez-Ramos M., Marquina S., Alvarez L. (2019). Achillin increases chemosensitivity to paclitaxel, overcoming resistance and enhancing apoptosis in human hepatocellular carcinoma cell line resistant to paclitaxel (Hep3B/PTX). Pharmaceutics.

[69] Woo S.M., Choi W.R., Lee D.R., Kim H.S., Yi C., Kim K.H., Kim H.L., Cheng J., Le B., Yang S.H. (2019). Leukodin isolated from *Artemisia capillaris* inhibits alpha-melanocyte stimulating hormone induced melanogenesis in B16F10 melanoma cells. Eur. J. Integr. Med..

[70] Zapata-Martínez J., Sánchez-Toranzo G., Chaín F., Cataláan C.A.N., Bühler M.I. (2016). Effect of guaianolides in the meiosis reinitiation of amphibian oocytes. Zygote.

[71] Zhang S.L., Li B.L., Li W., Lu M., Ni L.Y., Ma H.L., Meng Q.G. (2018). The effects of ludartin on cell proliferation, cell migration, cell cycle arrest and apoptosis are associated with upregulation of p21WAF1 in Saos-2 osteosarcoma cells in vitro. Med. Sci. Monit..

[72] Xu W., Miao S., Feng Y. (2019). Ludartin exhibits therapeutic effect on spinal cord injury through inhibition of apoptosis and inflammation. Bangladesh J. Pharmacol..

[73] Blanco J.G., Gil R.R., Alvarez C.I., Patrito L.C., Genti-Raimondi S., Flury A. (1997). A novel activity for a group of sesquiterpene lactones: Inhibition of aromatase. FEBS Lett..

[74] Giordano O.S., Guerreiro E., Pestchanker M.J., Guzman J., Pastor D., Guardia T. (1990). The gastric cytoprotective effect of Several sesquiterpene lactones. J. Nat. Prod..

[75] Luo H.J., Wang J.Z., Deng W.Q., Zou K. (2011). DFT calculations and docking study on sesquiterpene lactones: Inhibition of aromatase. Procedia Environ. Sci..

[76] Sülsen V.P., Lizarraga E.F., Elso O.G., Cerny N., Alberti A.S., Bivona A.E., Malchiodi E.L., Cazorla S.I., Catalán C.A.N. (2019). Activity of estafietin and analogues on *Trypanosoma cruzi* and *Leishmania braziliensis*. Molecules.

[77] Schepetkin I.A., Kirpotina L.N., Mitchell P.T., Kishkentaeva A.S., Shaimerdenova Z.R., Atazhanova G.A., Adekenov S.M., Quinn M.T. (2018). The natural sesquiterpene lactones arglabin, grosheimin, agracin, parthenolide, and estafiatin inhibit T cell receptor (TCR) activation. Phytochemistry.

[78] Liu Y., Meng Q., Jing L., Feng L., Zhou Z., Ni Z. (2020). 11, 13-Dehydro Lactone Moiety in Gynecologic Cancer Cells. Iran J. Public Health..

[79] Cai H., Meng X., Li Y., Yang C., Liu Y. (2014). Growth inhibition effects of isoalantolactone on K562/A02 cells: Caspase-dependent apoptotic pathways, S phase arrest, and downregulation of Bcr/Abl. Phytother. Res..

[80] Fan Y., Weng Z., Gao H., Hu J., Wang H., Li L. (2015). Isoalantolactone Enhances the Radiosensitivity of UMSCC-10A Cells via Specific Inhibition of Erk1/2 Phosphorylation. PLoS ONE.

[81] Wang J., Cui L., Feng L., Zhang Z., Song J., Liu D., Jia X. (2016). Isoalantolactone inhibits the migration and invasion of human breast cancer MDA-MB-231 cells via suppression of the p38 MAPK/NF-κB signaling pathway. Oncol. Rep..

[82] Weng Z., Gao H., Hu J., Fan Y., Wang H., Li L. (2016). Isoalantolactone induces autophagic cell death in SKOV₃ human ovarian carcinoma cells via upregulation of PEA-15. Oncol. Rep..

[83] Jin C., Zhang G., Zhang Y., Hua P., Song G., Sun M., Li X., Tong T., Li B., Zhang X. (2017). Isoalantolactone induces intrinsic apoptosis through p53 signaling pathway in human lung squamous carcinoma cells. PLoS ONE.

[84] Khan M., Ding C., Rasul A., Yi F., Li T., Gao H., Gao R., Zhong L., Zhang K., Fang X. (2012). Isoalantolactone induces reactive oxygen species mediated apoptosis in pancreatic carcinoma PANC-1 cells. Int. J. Biol. Sci..

[85] Rasul A., Khan M., Ali M., Li J., Li X. (2013). Targeting apoptosis pathways in cancer with alantolactone and isoalantolactone. Sci. World J..

[86] Yan Y.Y., Zhang Q., Zhang B., Yang B., Lin N.M. (2020). Active ingredients of *Inula helenium* L. exhibits similar anti-cancer effects as isoalantolactone in pancreatic cancer cells. Nat. Prod. Res..

[87] Zhou Y., Guo Y., Wen Z., Ci X., Xia L., Wang Y., Deng X., Wang J. (2020). Isoalantolactone enhances the antimicrobial activity of penicillin g against *Staphylococcus aureus* by inactivating β-lactamase during protein translation. Pathogens.

[88] Lu J., Kuang Z., Chen T., Ye C., Hou W., Tang L., Chen Y., He R. (2020). Isoalantolactone inhibits RANKL-induced osteoclast formation via multiple signaling pathways. Int. Immunopharmacol..

[89] Yuan C.-B., Tian L., Yang B., Zhou H.-Y. (2018). Isoalantolactone protects LPS-induced acute lung injury through Nrf2 activation. Microb. Pathog..

[90] Schmidt T.J., Brun R., Willuhn G., Khalid S.A. (2002). Anti-trypanosomal Activity of Helenalin and Some Structurally Related Sesquiterpene Lactones. Planta Med..

[91] Turk A., Ahn J.H., Jo Y.H., Song J.Y., Khalife H.K., Gali-Muhtasib H., Kim Y., Hwang B.Y., Lee M.K. (2019). NF-κB inhibitory sesquiterpene lactones from *Lebanese Laurus nobilis*. Phytochem. Lett..

[92] Coronado-Aceves E.W., Velázquez C., Robles-Zepeda R.E., Jiménez-Estrada M., Hernández-Martínez J., Gálvez-Ruiz J.C., Garibay-Escobar A. (2016). Reynosin and santamarine: Two sesquiterpene lactones from *Ambrosia confertiflora* with bactericidal activity against clinical strains of *Mycobacterium tuberculosis*. Pharm. Biol..

[93] Lim S., Lee S.J., Nam K.W., Kim K.H., Mar W. (2013). Hepatoprotective effects of reynosin against thioacetamide-induced apoptosis in primary hepatocytes and mouse liver. Arch. Pharm. Res..

[94] Ham A., Kim D.W., Kim K.H., Lee S.J., Oh K.B., Shin J., Mar W. (2013). Reynosin protects against neuronal toxicity in dopamine-induced SH-SY5Y cells and 6-hydroxydopamine-lesioned rats as models of Parkinson’s disease: Reciprocal up-regulation of E6-AP and down-regulation of α-synuclein. Brain Res..

[95] Mehmood T., Maryam A., Tian X., Khan M., Ma T. (2017). Santamarine inhibits NF-κB and STAT3 activation and induces apoptosis in HepG2 liver cancer cells via oxidative stress. J. Cancer.

[96] Choi H.G., Lee D.S., Li B., Choi Y.H., Lee S.H., Kim Y.C. (2012). Santamarin, a sesquiterpene lactone isolated from *Saussurea lappa*, represses LPS-induced inflammatory responses via expression of heme oxygenase-1 in murine macrophage cells. Int. Immunopharmacol..

[97] Jayaprakasam B., Alexander-Lindo R.L., DeWitt D.L., Nair M.G. (2007). Terpenoids from Stinking toe (*Hymneae courbaril*) fruits with cyclooxygenase and lipid peroxidation inhibitory activities. Food Chem..

[98] Cho B.O., Ryu H.W., So Y., Cho J.K., Woo H.S., Jin C.H., Seo K.I., Park J.C., Jeong I.Y. (2013). Anti-inflammatory effect of austroinulin and 6-O-acetyl-austroinulin from *Stevia rebaudiana* in lipopolysaccharide-stimulated RAW264.7 macrophages. Food Chem. Toxicol..

[99] Byun M. (2012). Anti-Inflammatory Activity of Austroinulin from *Stevia rebaudiana* in LPS-induced RAW264.7. Cells J. Korean Soc. Food Sci. Nutr..

[100] Vieira H.S., Takahashi J.A., De Oliveira A.B., Chiari E., Boaventura M.A.D. (2002). Novel derivatives of kaurenoic acid: Preparation and evaluation of their trypanocidal activity. J. Braz. Chem. Soc..

[101] Brito S., Crescente O., Fernández A., Coronado A., Rodriguez N. (2006). Efficacy of a kaurenic acid extracted from the Venezuelan plant *Wedelia trilobata (Asteracea)* against *Leishmania (Viannia) braziliensis*. Biomédica.

[102] Villasmil T., Rojas J., Aparicio R., Gamboa N., Acosta M.E., Rodrigues J., Usubillaga A. (2017). Antimalarial activity of some kaurenes. Nat. Prod. Commun..

[103] Mendoza C., Márquez A., Matheus N., Sosa S.M., López-Ortega A. (2017). Acción protectora del ácido kaurénico en el estrés oxidativo hepático. Rev. Vet..

[104] Sarwar M.S., Xia Y.X., Liang Z.M., Tsang S.W., Zhang H.J. (2020). Mechanistic pathways and molecular targets of plant-derived anticancer ent-kaurane diterpenes. Biomolecules.

[105] Sosa-Sequera M.C., Suárez O., Daló N.L. (2010). Kaurenic acid: An in vivo experimental study of its anti-inflammatory and antipyretic effects. Indian J. Pharmacol..

[106] Venditti A., Maggi F., Quassinti L., Bramucci M., Lupidi G., Ornano L., Ballero M., Sanna C., Bruno M., Rosselli S. (2018). Bioactive Constituents of *Juniperus turbinata Guss.* from La Maddalena Archipelago. Chem. Biodivers..

[107] De Las Heras B., Hoult J.R.S. (1994). Non-cytotoxic inhibition of macrophage eicosanoid biosynthesis and effects on leukocyte functions and reactive oxygen species of two novel anti-inflammatory plant diterpenoids. Planta Med..

[108] Balaei-Kahnamoei M., Eftekhari M., Ardekani M.R.S., Akbarzadeh T., Saeedi M., Jamalifar H., Safavi M., Sam S., Zhalehjoo N., Khanavi M. (2021). Phytochemical constituents and biological activities of *Salvia macrosiphon Boiss*. BMC Chem..

[109] Rasool N., Rashid M.A., Khan S.S., Ali Z., Zubair M., Ahmad V.U., Khan S.N., Choudhary M.I., Tareen R.B. (2013). Novel α-glucosidase activator from *Pulicaria undulata*. Nat. Prod. Commun..

[110] Castellar A., Coelho T.S., Silva P.E.A., Ramos D.F., Lourenço M.C.S., Lage C.L.S., Julião L.S., Barbosa Y.G., Leitão S.G. (2011). The activity of flavones and oleanolic acid from *Lippia lacunosa* against susceptible and resistant *Mycobacterium tuberculosis* strains. Rev. Bras. Farm..

[111] Shafaei A., Khan M.S.S., Aisha A.F.A., Majid A.M.S.A., Hamdan M.R., Mordi M.N., Ismail Z. (2016). Flavonoids-rich *Orthosiphon stamineus* extract as new candidate for angiotensin I-converting enzyme inhibition: A molecular docking study. Molecules.

[112] Yam M.F., Tan C.S., Ahmad M., Shibao R. (2016). Mechanism of vasorelaxation induced by eupatorin in the rats aortic ring. Eur. J. Pharmacol..

[113] Lee K., Lee D.H., Jung Y.J., Shin S.Y., Lee Y.H. (2016). The natural flavone eupatorin induces cell cycle arrest at the G2/M phase and apoptosis in HeLa cells. Appl. Biol. Chem..

[114] Razak N.A., Yeap S.K., Alitheen N.B., Ho W.Y., Yong C.Y., Tan S.W., Tan W.S., Long K. (2020). Eupatorin Suppressed Tumor Progression and Enhanced Immunity in a 4T1 Murine Breast Cancer Model. Integr. Cancer Ther..

[115] Gulçin İ., Taslimi P., Aygün A., Sadeghian N., Bastem E., Kufrevioglu O.I., Turkan F., Şen F. (2018). Antidiabetic and antiparasitic potentials: Inhibition effects of some natural antioxidant compounds on α-glycosidase, α-amylase and human glutathione S-transferase enzymes. Int. J. Biol. Macromol..

[116] Rajbhandari A., Roberts M.F. (1985). The Flavonoids of *Stevia microchaeta*, *Stevia monardifolia*, and *Stevia origanoides*. J. Nat. Prod..

[117] Sülsen V.P., Cazorla S.I., Frank F.M., Redko F.C., Anesini C.A., Coussio J.D., Malchiodi E.L., Martino V.S., Muschietti L.V. (2007). Trypanocidal and leishmanicidal activities of flavonoids from Argentine medicinal plants. Am. J. Trop. Med. Hyg..

[118] Melaku Y., Worku T., Tadesse Y., Mekonnen Y., Schmidt J., Arnold N., Dagne E. (2017). Antiplasmodial Compounds from Leaves of *Dodonaea angustifolia*. Curr. Bioact. Compd..

[119] Teffo L.S., Aderogba M.A., Eloff J.N. (2010). Antibacterial and antioxidant activities of four kaempferol methyl ethers isolated from *Dodonaea viscosa Jacq. var. angustifolia* leaf extracts. S. Afr. J. Bot..

[120] Mai L.H., Chabot G.G., Grellier P., Quentin L., Dumontet V., Poulain C., Espindola L.S., Michel S., Vo H.T.B., Deguin B. (2015). Antivascular and anti-parasite activities of natural and hemisynthetic flavonoids from *New Caledonian Gardenia* species (Rubiaceae). Eur. J. Med. Chem..

[121] Zhong M., Wang H.Q., Yan H.Y., Wu S., Gu Z.Y., Li Y.H. (2019). Santin inhibits influenza A virus replication through regulating MAPKs and NF-κB pathways. J. Asian Nat. Prod. Res..

[122] Stompor M. (2020). A review on sources and pharmacological aspects of sakuranetin. Nutrients.

[123] Ugocsai K., Varga A., Molnar P., Antus S., Molnar J. (2005). Effects of selected flavonoids and carotenoids on drug accumulation and apoptosis induction in multidrug-resistant colon cancer cells expressing MDR1/LRP. In Vivo.

[124] Park J.H., Fu Y.Y., Chung I.S., Hahn T.R., Cho M.H. (2013). Cytotoxic property of ultraviolet-induced rice phytoalexins to human colon carcinoma HCT-116 cell. J. Korean Soc. Appl. Biol. Chem..

[125] Drira R., Sakamoto K. (2016). Sakuranetin induces melanogenesis in B16BL6 melanoma cells through inhibition of ERK and PI3K/AKT signaling pathways. Phytother. Res..

[126] Hong L., Ying S.H. (2015). Ethanol extract and isolated constituents from *Artemisia dracunculus* inhibit esophageal squamous cell carcinoma and induce apoptotic cell death. Drug Res..

[127] Grecco D.S., Dorigueto A.C., Landre I.M., Soares M.G., Martho K., Lima R., Pascon R.C., Vallim M.A., Capello T.M., Romoff P. (2014). Structural crystalline characterization of sakuranetin—An antimicrobial flavanone from twigs of *Baccharis retusa* (Asteraceae). Molecules.

[128] Pacciaroni A.V., Gette M.A., Derita M., Luis Ariza-Espinar L., Gil R.R., Zacchino S.A., Silva G.L. (2008). Antifungal Activity of *Heterothalamus alienus* Metabolites. Phytother. Res..

[129] Zhang L., Kong Y., Wu D., Zhang H., Wu J., Chen J., Ding J., Hu L., Jiang H., Shen X. (2008). Three flavonoids targeting the β-hydroxyacyl-acyl carrier protein dehydratase from *Helicobacter pylori*: Crystal structure characterization with enzymatic inhibition assay. Protein Sci..

[130] Grecco S.D.S., Reimão J.Q., Tempone A.G., Sartorelli P., Cunha R.L.O.R., Romoff P., Ferreira M.J.P., Fávero O.A., Lago J.H.G. (2012). In vitro antileishmanial and antitrypanosomal activities of flavanones from *Baccharis retusa* DC. (Asteraceae). Exp. Parasitol..

[131] Quintanilla-Licea R., Vargas-Villarreal J., Verde-Star M.J., Rivas-Galindo V.M., Torres-Hernández Á.D. (2020). Antiprotozoal Activity against *Entamoeba histolytica* of Flavonoids Isolated from *Lippia graveolens* Kunth. Molecules.

[132] Kwon D.H., Ji J.H., Yim S.H., Kim B.S., Choi H.J. (2018). Suppression of influenza B virus replication by sakuranetin and mode of its action. Phytother. Res..

[133] Choi H.J. (2017). In vitro antiviral activity of sakuranetin against human rhinovirus 3. Osong Public Health Res. Perspect..

[134] Bittencourt-Mernak M.I., Pinheiro N.M., Santana F.P.R., Guerreiro M.P., Saraiva-Romanholo B.M., Grecco S.S., Caperuto L.C., Felizardo R.J.F., Câmara N.O.S., Tibério I.F.L.C. (2017). Prophylactic and therapeutic treatment with the flavonone sakuranetin ameliorates LPS-induced acute lung injury. Am. J. Physiol. Lung C.

[135] Sakoda C.P.P., de Toledo A.C., Perini A., Pinheiro N.M., Hiyane M.I., Grecco S.d.S., de Fátima Lopes Calvo Tibério I., Câmara N.O.S., de Arruda Martins M., Lago J.G.H. (2016). Sakuranetin reverses vascular peribronchial and lung parenchyma remodeling in a murine model of chronic allergic pulmonary inflammation. Acta Histochem..

[136] Taguchi L., Pinheiro N.M., Choqueta-Toledo A., Grecco S.S., Lopes F.D., Caperuto L.C., Martins L.C., Tiberio I.F., Câmara N.O., Lago J.H. (2015). A flavanone from *Baccharis retusa* (Asteraceae) prevents elastase-induced emphysema in mice by regulating NF-κB, oxidative stress and metalloproteinases. Respir. Res..

[137] Toledo A.C., Sakoda C.P.P., Perini A., Pinheiro N.M., Magalhães R.M., Grecco S., Tibério I.F.L.C., Cãmara N.O., Martins M.A., Lago J.H.G. (2013). Flavanone treatment reverses airway inflammation and remodeling in an asthma murine model. Br. J. Pharmacol..

[138] Yamauchi Y., Okuyama T., Ishii T., Okumura T., Ikeya Y., Nishizawa M. (2019). Sakuranetin downregulated inducible nitric oxide synthase expression by affecting interleukin-1 receptor and CCAAT/enhancer-binding protein β. J. Nat. Med..

[139] Zhang X., Hung T.M., Phuong P.T., Ngoc T.M., Min B.-S., Song K.-S., Seong Y.H., Bae K. (2006). Anti-inflammatory activity of flavonoids from *Populus davidiana*. Arch. Pharm. Res..

[140] Hernández V., Recio M.C., Máñez S., Giner R.M., Rios J.L. (2007). Effects of naturally occuring dihydroflavonols from *Inula viscosa* on inflammation and enzymes involved in the arachidonic acid metabolism. Life Sci..

[141] Chen L., Hu C. (2019). Protective effect of sakuranetin in brain cells of dementia model rats. Cell. Mol. Biol..

[142] Grecco D.S., Félix M.J.P., Lago J.H.G., Pinto É.G., Tempone A.G., Romoff P., Ferreira M.J.P., Sartorelli P. (2014). Anti-trypanosomal phenolic derivatives from *Baccharis uncinella*. Nat. Prod. Commun..

[143] Muthu C., Reegan A.D., Kingsley S., Ignacimuthu S. (2012). Larvicidal activity of pectolinaringenin from *Clerodendrum phlomidis* L. against *Culex quinquefasciatus Say* and *Aedes aegypti* L. (Diptera: Culicidae). Parasitol. Res..

[144] Tasdemir D., Kaiser M., Brun R., Yardley V., Schmidt T.J., Tosun F., Ruedi P. (2006). Antitrypanosomal and Antileishmanial Activities of Flavonoids and Their Analogues: In Vitro, In Vivo, Structure-Activity Relationship, and Quantitative Structure-Activity Relationship Studies. Antimicrob. Agents Chemother..

[145] Abdelhalim A., Chebib M., Aburjai T., Johnston G.A.R., Hanrahan J.R. (2014). GABAA Receptor Modulation by Compounds Isolated from *Salvia triloba* L.. Adv. Biol. Chem..

[146] Abdelhalim A., Karim N., Chebib M., Aburjai T., Khan I., Johnston G.A.R., Hanrahan J.R. (2015). Antidepressant, anxiolytic and antinociceptive activities of constituents from *Rosmarinus officinalis*. J. Pharm. Pharm. Sci..

[147] Hernández-Bolio G.I., Torres-Tapia L.W., Moo-Puc R., Peraza-Sánchez S.R. (2015). Antigiardial activity of flavonoids from leaves of *Aphelandra scabra*. Rev. Bras. Farmacogn..

[148] Kim H.J., Kim I.S., Dong Y., Lee I.S., Kim J.S., Kim J.S., Woo J.T., Cha B.Y. (2015). Melanogenesis-inducing effect of cirsimaritin through increases in microphthalmia-associated transcription factor and tyrosinase expression. Int. J. Mol. Sci..

[149] Wu Z.K., Wang J.J., Zhu S.S., Zhang J.Y., Wei J.H., Li L. (2016). Cirsimaritin ameliorates cardiac remodeling and dysfunction through promoting myocardial autophagy in rats with heart failure. Int. J. Clin. Exp. Pathol..

[150] Lee D., Jung Y., Baek J.Y., Shin M.S., Lee S., Hahm D.H., Lee S.C., Shim J.S., Kim S.N., Kang K.S. (2017). Cirsimaritin Contributes to the Estrogenic Activity of *Cirsium japonicum* var. maackii through the Activation of Estrogen Receptor α. Bull. Korean Chem. Soc..

[151] Abbas G., Al Harrasi A., Hussain H., Hamaed A., Supuran C.T. (2019). The management of diabetes mellitus-imperative role of natural products against dipeptidyl peptidase-4, α-glucosidase and sodium-dependent glucose co-transporter 2 (SGLT2). Bioorganic Chem..

[152] Park J.Y., Kim H.Y., Shibamoto T., Jang T.S., Lee S.C., Shim J.S., Hahm D.H., Lee H.J., Lee S., Kang K.S. (2017). Beneficial effects of a medicinal herb, *Cirsium japonicum var. maackii*, extract and its major component, cirsimaritin on breast cancer metastasis in MDA-MB-231 breast cancer cells. Bioorg. Med. Chem. Lett..

[153] Shin M.S., Park J.Y., Lee J., Yoo H.H., Hahm D.H., Lee S.C., Lee S., Hwang G.S., Jung K., Kang K.S. (2017). Anti-inflammatory effects and corresponding mechanisms of cirsimaritin extracted from *Cirsium japonicum var*. Maackii Maxim. Bioorganic Med. Chem. Lett..

[154] Yan H., Wang H., Ma L., Ma X., Yin J., Wu S., Huang H., Li Y. (2018). Cirsimaritin inhibits influenza A virus replication by downregulating the NF-κB signal transduction pathway. Virol. J..

[155] Manurung K., Sulastri D., Zubir N., Ilyas S. (2020). In silico anticancer activity and in vitro antioxidant of flavonoids in *Plectranthus amboinicus*. Pharmacogn. J..

[156] Pathak G., Singh S., Kumari P., Raza W., Hussain Y., Meena A. (2020). Cirsimaritin, a lung squamous carcinoma cells (NCIH-520) proliferation inhibitor. J. Biomol. Struct. Dyn..

[157] Thanasansurapong S., Tuchinda P., Reutrakul V., Pohmakotr M., Piyachaturawat P., Chairoungdua A., Suksen K., Akkarawongsapat R., Limthongkul J., Napaswad C. (2020). Cytotoxic and anti-HIV-1 activities of triterpenoids and flavonoids isolated from leaves and twigs of *Gardenia sessiliflora*. Phytochem. Lett..

[158] Bourdillat B., Delautier D., Labat C., Benveniste J., Potier P., Brink C. (1988). Hispidulin, a natural flavone, inhibits human platelet aggregation by increasing cAMP levels. Eur. J. Pharmacol..

[159] Marques M.R., Stüker C., Kichik N., Tarragó T., Giralt E., Morel A.F., Dalcol I.I. (2010). Flavonoids with prolyl oligopeptidase inhibitory activity isolated from *Scutellaria racemosa Pers*. Fitoterapia.

[160] Mercader A.G., Pomilio A.B. (2010). QSAR study of flavonoids and biflavonoids as influenza H1N1 virus neuraminidase inhibitors. Eur. J. Med. Chem..

[161] Yu C.Y., Su K.Y., Lee P.L., Jhan J.Y., Tsao P.H., Chan D.C., Chen Y.L.S. (2013). Potential therapeutic role of hispidulin in gastric cancer through induction of apoptosis via NAG-1 signaling. Evid. Based Complementary Altern. Med..

[162] Xu Q., Xie H., Wu P., Wei X. (2013). Flavonoids from the capitula of *Eriocaulon australe*. Food Chem..

[163] Xie W., Li H., Zhu J. (2007). Antitumor effect of hispidulin in vivo and in vitro. Mater. Med..

[164] Reutrakul V., Krachangchaeng C., Tuchinda P., Pohmarkotr M., Jaipetch T., Yoosook C., Kasisit J., Sophasan S., Sujarit K., Santisuk T. (2004). Cytotoxic and anti-HIV-1 constituents from leaves and twigs of *Gardenia tubifera*. Tetrahedron.

[165] Nepal M., Choi H.J., Choi B.Y., Yang M.S., Chae J.I., Li L., Soh Y. (2013). Hispidulin attenuates bone resorption and osteoclastogenesis via the RANKL-induced NF-κB and NFATc1 pathways. Eur. J. Pharmacol..

[166] Kim H.A., Lee J. (2021). Hispidulin modulates epithelial-mesenchymal transition in breast cancer cells. Oncol. Lett..

[167] Liu K., Zhao F., Yan J., Xia Z., Jiang D., Maa P. (2020). Hispidulin: A promising flavonoid with diverse anti-cancer properties. Life Sci..

[168] Serti‘e J.A.A., Basile A.C., Panizza S., Matida A.K., Zelnik R. (1990). Anti-inflammatory activity and sub-acute toxicity of artemetin. Planta Med..

[169] Grossini E., Marotta P., Farruggio S., Sigaudo L., Qoqaiche F., Raina G., De Giuli V., Mary D., Vacca G., Pollastro F. (2015). Effects of Artemetin on Nitric Oxide Release and Protection against Peroxidative Injuries in Porcine Coronary Artery Endothelial Cells. Phytother. Res..

[170] Hu J., Ma W., Li N., Wang K.J. (2017). Antioxidant and anti-inflammatory flavonoids from the flowers of chuju, a medical cultivar of chrysanthemum morifolim ramat. J. Mex. Chem. Soc..

[171] De Souza P., Gasparotto A., Crestani S., Élida M., Stefanello A., Consuelo M., Marques A., Eduardo J., Aparecida C., Kassuya L. (2011). Phytomedicine Hypotensive mechanism of the extracts and artemetin isolated from *Achillea millefolium* L. (Asteraceae) in rats. Eur. J. Integr. Med..

[172] Liu K.C., Yang S.L., Roberts M.F., Elford B.C., Phillipson J.D. (1992). Antimalarial activity of *Artemisia annua* flavonoids from whole plants and cell cultures. Plant. Cell Rep..

[173] Nwodo N., Okoye F., Lai D., Debbab A., Kaiser M., Brun R., Proksch P. (2015). Evaluation of the in vitro trypanocidal activity of methylated flavonoid constituents of *Vitex simplicifolia* leaves. BMC Complement. Altern. Med..

[174] Wee H.N., Neo S.Y., Singh D., Yew H.C., Qiu Z.Y., Tsai X.R.C., How S.Y., Yip K.Y.C., Tan C.H., Koh H.L. (2020). Effects of *Vitex trifolia* L. Leaf extracts and phytoconstituents on cytokine production in human u937 macrophages. BMC Complement. Altern. Med..

[175] Ono M., Yanaka T., Yamamoto M., Ito Y., Nohara T. (2002). New diterpenes and norditerpenes from the fruits of *Vitex rotundifolia*. J. Nat. Prod..

[176] Boniface P.K., Ferreira E.I. (2019). Flavonoids as efficient scaffolds: Recent trends for malaria, leishmaniasis, Chagas disease, and dengue. Phytother. Res..

[177] Yuting C., Rongliang Z., Zhngjian J., Yong J. (1990). Flavonoids as superoxide scavengers and antioxidants. Free Radic. Biol. Med..

[178] Mlcek J., Jurikova T., Skrovankova S., Sochor J. (2016). Quercetin and its anti-allergic immune response. Molecules.

[179] Li Y., Yao J., Han C., Yang J., Chaudhry M.T., Wang S., Liu H., Yin Y. (2016). Quercetin, inflammation and immunity. Nutrients.

[180] Marunaka Y., Marunaka R., Sun H., Yamamoto T., Kanamura N., Inui T., Taruno A. (2017). Actions of quercetin, a polyphenol, on blood pressure. Molecules.

[181] Eid H.M., Haddad P.S. (2017). The Antidiabetic Potential of Quercetin: Underlying Mechanisms. Curr. Med. Chem..

[182] Rauf A., Imran M., Khan I.A., ur-Rehman M., Gilani S.A., Mehmood Z., Mubarak M.S. (2018). Anticancer potential of quercetin: A comprehensive review. Phytother. Res..

[183] Shafabakhsh R., Asemi Z. (2019). Quercetin: A natural compound for ovarian cancer treatment. J. Ovarian Res..

[184] Khan H., Ullah H., Aschner M., Cheang W.S., Akkol E.K. (2020). Neuroprotective effects of quercetin in Alzheimer’s disease. Biomolecules.

[185] Li J., Jiang H., Shi R. (2009). A new acylated quercetin glycoside from the leaves of *Stevia rebaudiana* Bertoni. Nat. Prod. Res..

[186] Li Y., Di Frenz C.M., Chen M.H., Wang Y.R., Li F.J., Luo C., Liang N., Yang H., Bohlin L., Wang C.L. (2011). Primary Virtual and in vitro Bioassay Screening of Natural Inhibitors from Flavonoids against COX-2. Chin. J. Nat. Med..

[187] Nguyen M.T.T., Awale S., Tezuka Y., Ueda J.Y., Le Tran Q., Kadota S. (2006). Xanthine oxidase inhibitors from the flowers of *Chrysanthemum sinense*. Planta Med..

[188] Krasteva I., Bratkov V., Bucar F., Kunert O., Kollroser M., Kondeva-Burdina M., Ionkova I. (2015). Flavoalkaloids and Flavonoids from *Astragalus monspessulanus*. J. Nat. Prod..

[189] Karunarathne W.A.H.M., Lee K.T., Choi Y.H., Jin C.Y., Kim G.Y. (2020). Anthocyanins isolated from *Hibiscus syriacus* L. attenuate lipopolysaccharide-induced inflammation and endotoxic shock by inhibiting the TLR4/MD2-mediated NF-κB signaling pathway. Phytomedicine.

[190] Minda D., Avram S., Pavel I.Z., Kis B., Ghitu A., Zupko I., Dehelean C., Buda V., DIaconeasa Z., Scurtu A. (2020). An in vitro evaluation of apigenin and apigenin-7-o-glucoside against hela human cervical cancer cell line. Rev. Chim..

[191] Villa-Rodriguez J.A., Kerimi A., Tumova S., Williamson G. (2019). Inhibition of intestinal glucose transport by polyphenols: A mechanism for indirect attenuation of cholesterol absorption. Food Funct..

[192] Jia Y., Ma Y., Cheng G., Zhang Y., Cai S. (2019). Comparative Study of Dietary Flavonoids with Different Structures as α-Glucosidase Inhibitors and Insulin Sensitizers. J. Agric. Food Chem..

[193] Ramchandani S., Naz I., Lee J.H., Khan M.R., Ahn K.S. (2020). An overview of the potential antineoplastic effects of casticin. Molecules.

[194] Koh D.J., Ahn H.S., Chung H.S., Lee H., Kim Y., Lee J.Y., Kim D.G., Hong M., Shin M., Bae H. (2011). Inhibitory effects of casticin on migration of eosinophil and expression of chemokines and adhesion molecules in A549 lung epithelial cells via NF-κB inactivation. J. Ethnopharmacol..

[195] Bergendorff O., Sterner O. (1995). Spasmolytic Flavonols from *Artemisia abrotanum*. Planta Med..

[196] Hu Y., Xin H.L., Zhang Q.Y., Zheng H.C., Rahman K., Qin L.P. (2007). Anti-nociceptive and anti-hyperprolactinemia activities of *Fructus viticis* and its effective fractions and chemical constituents. Phytomedicine.

[197] Webster D.E., He Y., Chen S.N., Pauli G.F., Farnsworth N.R., Wang Z.J. (2011). Opioidergic mechanisms underlying the actions of *Vitex agnus-castus* L.. Biochem. Pharmacol..

[198] Chu J., Yan B., Zhang J., Peng L., Ao X., Zheng Z., Jiang T., Zhang Z. (2020). Casticin Attenuates Osteoarthritis-Related Cartilage Degeneration by Inhibiting the ROS-Mediated NF-κB Signaling Pathway in vitro and in vivo. Inflammation.

[199] Nageen B., Sarfraz I., Rasul A., Hussain G., Rukhsar F., Irshad S., Riaz A., Selamoglu Z., Ali M. (2018). Eupatilin: A natural pharmacologically active flavone compound with its wide range applications. J. Asian Nat. Prod. Res..

[200] Li Y., Ren R., Wang L., Peng K. (2020). Eupatilin alleviates airway remodeling via regulating phenotype plasticity of airway smooth muscle cells. Biosci. Rep..

[201] Jeong J.H., Moon S.J., Jhun J.Y., Yang E.J., Cho M.L., Min J.K. (2015). Eupatilin exerts antinociceptive and chondroprotective properties in a rat model of osteoarthritis by downregulating oxidative damage and catabolic activity in chondrocytes. PLoS ONE.

[202] Zhang Y., Qin L., Xie J., Li J., Wang C. (2020). Eupatilin prevents behavioral deficits and dopaminergic neuron degeneration in a Parkinson’s disease mouse model. Life Sci..

[203] Kang Y.J., Jung U.J., Lee M.K., Kim H.J., Jeon S.M., Park Y.B., Chung H.G., Baek N.I., Lee K.T., Jeong T.S. (2008). Eupatilin, isolated from *Artemisia princeps Pampanini*, enhances hepatic glucose metabolism and pancreatic β-cell function in type 2 diabetic mice. Diabetes Res. Clin. Pract..

[204] Son J.E., Lee E., Seo S.G., Lee J., Kim J.E., Kim J., Lee K.W., Lee H.J. (2013). Eupatilin, a major flavonoid of artemisia, attenuates aortic smooth muscle cell proliferation and migration by inhibiting PI3K, MKK3/6, and MKK4 activities. Planta Med..

[205] Metoui R., Bouajila J., Znati M., Cazaux S., Neffati M., Akrout A. (2017). Bioactive flavones isolated from Tunisian *Artemisia campestris* L. Leaves. Cell. Mol. Biol..

[206] Chougouo R.D.K., Nguekeu Y.M.M., Dzoyem J.P., Awouafack M.D., Kouamouo J., Tane P., McGaw L.J., Eloff J.N. (2016). Anti-inflammatory and acetylcholinesterase activity of extract, fractions and five compounds isolated from the leaves and twigs of *Artemisia annua* growing in Cameroon. SpringerPlus.

[207] Ebada S.S., Al-Jawabri N.A., Youssef F.S., El-Kashef D.H., Knedel T.O., Albohy A., Korinek M., Hwang T.L., Chen B.H., Lin G.H. (2020). Anti-inflammatory, antiallergic and COVID-19 protease inhibitory activities of phytochemicals from the *Jordanian hawksbeard*: Identification, structure-Activity relationships, molecular modeling and impact on its folk medicinal uses. RSC Adv..

[208] Zhu Q.C., Wang Y., Liu Y.P., Zhang R.Q., Li X., Su W.H., Long F., Luo X.D., Peng T. (2011). Inhibition of enterovirus 71 replication by chrysosplenetin and penduletin. Eur. J. Pharm. Sci..

[209] Ortiz S., Dali-Yahia K., Vasquez-Ocmin P., Grougnet R., Grellier P., Michel S., Maciuk A., Boutefnouchet S. (2017). Heme-binding activity of methoxyflavones from Pentzia monodiana Maire (*Asteraceae*). Fitoterapia.

[210] Nurbek S., Murata T., Suganuma K., Ishikawa Y., Buyankhishig B., Kikuchi T., Byambajav T., Davaapurev B.O., Sasaki K., Batkhuu J. (2020). Isolation and evaluation of trypanocidal activity of sesquiterpenoids, flavonoids, and lignans in *Artemisia sieversiana* collected in Mongolia. J. Nat. Med..

[211] Arroo R.R.J., Sari S., Barut B., Özel A., Ruparelia K.C., Şöhretoğlu D. (2020). Flavones as tyrosinase inhibitors: Kinetic studies in vitro and in silico. Phytochem. Anal..

[212] Cao Y., Zang Y., Huang X., Cheng Z. (2019). Chemical constituents from *Artemisia rupestris* and their neuraminidase inhibitory activity. Nat. Prod. Res..

[213] Ma L., Wei S., Yang B., Ma W., Wu X., Ji H., Sui H., Chen J. (2017). Chrysosplenetin inhibits artemisinin efflux in Pgp- over-expressing Caco-2 cells and reverses P-gp/MDR1 mRNA up-regulated expression induced by artemisinin in mouse small intestine. Pharm. Biol..

[214] Hong G., He X., Shen Y., Chen X., Yang F., Yang P., Pang F., Han X., He W., Wei Q. (2019). Chrysosplenetin promotes osteoblastogenesis of bone marrow stromal cells via Wnt/β-catenin pathway and enhances osteogenesis in estrogen deficiency-induced bone loss. Stem Cell Res. Ther..

[215] Forgo P., Zupkó I., Molnár J., Vasas A., Dombi G., Hohmann J. (2012). Bioactivity-guided isolation of antiproliferative compounds from *Centaurea jacea* L.. Fitoterapia.

[216] Ahmed S., Kamel E.M. (2014). Cytotoxic activities of flavonoids from *Centaurea scoparia*. Sci. World J..

[217] Jachak S.M., Gautam R., Selvam C., Madhan H., Srivastava A., Khan T. (2011). Anti-inflammatory, cyclooxygenase inhibitory and antioxidant activities of standardized extracts of *Tridax procumbens* L.. Fitoterapia.

[218] Chang S.L., Chiang Y.M., Chang C.L.T., Yeh H.H., Shyur L.F., Kuo Y.H., Wu T.K., Yang W.C. (2007). Flavonoids, centaurein and centaureidin, from *Bidens pilosa*, stimulate IFN-γ expression. J. Ethnopharmacol..

[219] Qaddir I., Majeed A., Hussain W., Mahmood S., Rasool N. (2020). An in silico investigation of phytochemicals as potential inhibitors against non-structural protein 1 from dengue virus 4. Braz. J. Pharm..

[220] Ito Y., Kanamaru A., Tada A. (2006). Centaureidin promotes dendrite retraction of melanocytes by activating Rho. Biochim. Biophys. Acta Gen. Subj..

[221] Zater H., Huet J., Fontaine V., Benayache S., Stévigny C., Duez P., Benayache F. (2016). Chemical constituents, cytotoxic, antifungal and antimicrobial properties of *Centaurea diluta Ait. subsp. algeriensis* (Coss. Dur.) *Maire*. Asian Pac. J. Trop. Med..

[222] Şekerler T., Şen A., Bitiş L., Şener A. (2020). In vitro antihepatocellular carcinoma activity of secondary metabolites of *Centaurea kilaea boiss*. J. Res. Pharm..

[223] Lee H.G., Yu K.A., Oh W.K., Baeg T.W., Oh H.C., Ahn J.S., Jang W.C., Kim J.W., Lim J.S., Choe Y.K. (2005). Inhibitory effect of jaceosidin isolated from *Artemisia argyi* on the function of E6 and E7 oncoproteins of HPV 16. J. Ethnopharmacol..

[224] Kim M.J., Han J.M., Jin Y.Y., Baek N.I., Bang M.H., Chung H.G., Choi M.S., Lee K.T., Sok D.E., Jeong T.S. (2008). In vitro antioxidant and anti-inflammatory activities of jaceosidin from *Artemisia princeps Pampanini cv*. Sajabal. Arch. Pharm. Res..

[225] Park E., Hong K., Kwon B.M., Kim Y., Kim J.H. (2020). Jaceosidin ameliorates insulin resistance and kidney dysfunction by enhancing insulin receptor signaling and the antioxidant defense system in type 2 diabetic mice. J. Med. Food.

[226] Lee S.H., Bae E.A., Park E.K., Shin Y.W., Baek N.I., Han E.J., Chung H.G., Kim D.H. (2007). Inhibitory effect of eupatilin and jaceosidin isolated from *Artemisia princeps* in IgE-induced hypersensitivity. Int. Immunopharmacol..

[227] Min S.W., Kim N.J., Baek N.I., Kim D.H. (2009). Inhibitory effect of eupatilin and jaceosidin isolated from *Artemisia princeps* on carrageenan-induced inflammation in mice. J. Ethnopharmacol..

[228] Lee H., Jang D., Jeon J., Cho C., Choi S., Han S.J., Oh E., Nam J., Park C.H., Shin Y.S. (2020). Seomae mugwort and jaceosidin attenuate osteoarthritic cartilage damage by blocking IκB degradation in mice. J. Cell. Mol. Med..

[229] Kumar R., Lu Y., Elliott A.G., Kavanagh A.M., Cooper M.A., Davis R.A. (2016). Semi-synthesis and NMR spectral assignments of flavonoid and chalcone derivatives. Magn. Reson. Chem..

[230] Allison B.J., Allenby M.C., Bryant S.S., Min J.E., Hieromnimon M., Joyner P.M. (2017). Antibacterial activity of fractions from three Chumash medicinal plant extracts and in vitro inhibition of the enzyme enoyl reductase by the flavonoid jaceosidin. Nat. Prod. Res..

[231] Lee T.H., Jung H., Park K.H., Bang M.H., Baek N.I., Kim J. (2014). Jaceosidin, a natural flavone, promotes angiogenesis via activation of VEGFR2/FAK/PI3K/AKT/NF-iB signaling pathways in endothelial cells. Exp. Biol. Med..

[232] Afifi F.U., Aburjai T. (2004). Antiplatelet activity of *Varthemia iphionoides*. Fitoterapia.

[233] Aljančić I., Stanković M., Tešević V., Vujisić L., Vajs V., Milosavljević S. (2010). Protective effect on human lymphocytes of some flavonoids isolated from two *Achillea* species. Nat. Prod. Commun..

[234] Elhady S.S., Eltamany E.E., Shaaban A.E., Bagalagel A.A., Muhammad Y.A., El-Sayed N.M., Ayyad S.N., Ahmed A.A.M., Elgawish M.S., Ahmed S.A. (2020). Jaceidin flavonoid isolated from *Chiliadenus montanus* attenuates tumor progression in mice via vegf inhibition: In vivo and in silico studies. Plants.

[235] Camuesco D., Comalada M., Rodríguez-Cabezas M.E., Nieto A., Lorente M.D., Concha A., Zarzuelo A., Gálvez J. (2004). The intestinal anti-inflammatory effect of quercitrin is associated with an inhibition in iNOS expression. Br. J. Pharmacol..

[236] Cincin Z.B., Unlu M., Kiran B., Bireller E.S., Baran Y., Cakmakoglu B. (2014). Molecular mechanisms of quercitrin-induced apoptosis in non-small cell lung cancer. Arch. Med. Res..

[237] Chiow K.H., Phoon M.C., Putti T., Tan B.K.H., Chow V.T. (2016). Evaluation of antiviral activities of *Houttuynia cordata* Thunb. extract, quercetin, quercetrin and cinanserin on murine coronavirus and dengue virus infection. Asian Pac. J. Trop. Med..

[238] Xing L.-Z., Ni H.-J., Wang Y.-L. (2017). Quercitrin attenuates osteoporosis in ovariectomized rats by regulating mitogen-activated protein kinase (MAPK) signaling pathways. Biomed. Pharmacother..

[239] Kim J., Re Kim S., Choi Y.H., Shin J.Y., Kim C.D., Kang N.G., Park B.C., Lee S. (2020). Quercitrin Stimulates Hair Growth with Enhanced Expression of Growth Factors via Activation of MAPK/CREB Signaling Pathway. Molecules.

[240] Hur H.J., Jeong Y.H., Lee S.H., Sung M.J. (2020). Quercitrin ameliorates hyperlipidemia and hepatic steatosis in ovariectomized mice. Life.

[241] Zhang X., Cheng B., Liu X., Li Y., Hou J., Chen S., Chen J., Li S. (2020). Screening of α-Glucosidase Inhibitors from *Houttuynia cordata* and Evaluation of the Binding Mechanisms. Chem. Sel..

[242] Jegal J., Park N.J., Lee S.Y., Jo B.G., Bong S.K., Kim S.N., Yang M.H. (2020). Quercitrin, the Main Compound in *Wikstroemia indica*, Mitigates Skin Lesions in a Mouse Model of 2,4-Dinitrochlorobenzene-Induced Contact Hypersensitivity. Evid. Based Complement. Altern. Med..

[243] Razafin-drabazo F., Donno D., Tombozara N., Razafindrakoto Z.R., Rajaonarison J.F., Andrianjara C., Ramanitrahasimbola D., Beccaro G.L. (2020). Phyto-compounds and pharmacological activities of *Lygodium lanceolatum* Desv. (Schizaeaceae). S. Afr. J. Bot..

[244] Oh T.W., Do H.J., Jeon J.H., Kim K. (2021). Quercitrin inhibits platelet activation in arterial thrombosis. Phytomedicine.

[245] Guo H., Yin W., Zou Z., Zhang C., Sun M., Min L., Yang L., Kong L. (2021). Quercitrin alleviates cartilage extracellular matrix degradation and delays ACLT rat osteoarthritis development: An in vivo and in vitro study. J. Adv. Res..

[246] Militão G.C.G., Albuquerque M.R.J.R., Pessoa O.D.L., Pessoa C., Moraes M.E.A., De Moraes M.O., Costa-Lotufo L.V. (2004). Cytotoxic activity of nepetin, a flavonoid from *Eupatorium ballotaefolium* HBK. Pharmazie.

[247] Jiang H., Wu D., Xu D., Yu H., Zhao Z., Ma D., Jin J. (2017). Eupafolin exhibits potent anti-angiogenic and antitumor activity in hepatocellular carcinoma. Int. J. Biol. Sci..

[248] Wang C.Y., Huang S.C., Lai Z.R., Ho Y.L., Jou Y.J., Kung S.H., Zhang Y., Chang Y.S., Lin C.W. (2013). Eupafolin and ethyl acetate fraction of *Kalanchoe gracilis* stem extract show potent antiviral activities against enterovirus 71 and coxsackievirus A16. Evid. Based Complement. Altern. Med..

[249] Yang J., Wang X., Zhang C., Ma L., Wei T., Zhao Y., Peng X. (2021). Comparative study of inhibition mechanisms of structurally different flavonoid compounds on α-glucosidase and synergistic effect with acarbose. Food Chem..

[250] Lee C.W., Lin Z.C., Hsu L.F., Fang J.Y., Chiang Y.C., Tsai M.H., Lee M.H., Li S.Y., Hu S.C.S., Lee I.T. (2016). Eupafolin ameliorates COX-2 expression and PGE2 production in particulate pollutants-exposed human keratinocytes through ROS/MAPKs pathways. J. Ethnopharmacol..

[251] Chen X., Han R., Hao P., Wang L., Liu M., Jin M., Kong D., Li X. (2018). Nepetin inhibits IL-1β induced inflammation via NF-κB and MAPKs signaling pathways in ARPE-19 cells. Biomed. Pharmacother..

[252] Vo V.A., Lee J.W., Chang J.E., Kim J.Y., Kim N.H., Lee H.J., Kim S.S., Chun W., Kwon Y.S. (2012). Avicularin inhibits lipopolysaccharide-induced inflammatory response by suppressing ERK phosphorylation in RAW264.7 macrophages. Biomol. Ther..

[253] Wang Z., Li F., Quan Y., Shen J. (2019). Avicularin ameliorates human hepatocellular carcinoma via the regulation of NF-κB/COX-2/PPAR-γ activities. Mol. Med. Rep..

[254] Wang W.E.I., Zheng H., Zheng M., Liu X., Yu J. (2018). Protective effect of avicularin on rheumatoid arthritis and its associated mechanisms. Exp. Ther. Med..

[255] Shen Z., Xu Y., Jiang X., Wang Z., Guo Y., Pan W., Hou J. (2019). Avicularin relieves depressive-like behaviors induced by chronic unpredictable mild stress in mice. Med. Sci. Monit..

[256] Fujimori K., Shibano M. (2013). Avicularin, a plant flavonoid, suppresses lipid accumulation through repression of C/EBPα-activated GLUT4-mediated glucose uptake in 3T3-L1 cells. J. Agric. Food Chem..

[257] Ko W.C., Kuo S.W., Sheu J., Lin C., Tzeng S., Chen C. (1999). Relaxant Effects of Quercetin Methyl Ether Derivatives in Isolated Guinea Pig Trachea and their Structure-Activity Relationships. Planta Med..

[258] Guerrero M.F., Puebla P., Martín M.L., Carrón R., San Román L., Reguero M.T., Arteaga L. (2002). Inhibitory effect of N(G)-nitro-L-arginine methyl ester on the anti-adrenergic response elicited by ayanin in the pithed rat. Planta Med..

[259] Guerrero M.F., Puebla P., Carrón R., Martín M.L., Román L.S. (2002). Quercetin 3,7-dimethyl ether: A vasorelaxant flavonoid isolated from *Croton schiedeanus* Schlecht. J. Pharm. Pharmacol..

[260] Kawai M., Hirano T., Higa S., Arimitsu J., Maruta M., Kuwahara Y., Ohkawara T., Hagihara K., Yamadori T., Shima Y. (2007). Flavonoids and related compounds as anti-allergic substances. Allergol. Int..

[261] Pick A., Müller H., Mayer R., Haenisch B., Pajeva I.K., Weigt M., Bönisch H., Müller C.E., Wiese M. (2011). Structure-activity relationships of flavonoids as inhibitors of breast cancer resistance protein (BCRP). Bioorg. Med. Chem..

[262] Mahmoud A.B., Danton O., Kaiser M., Khalid S., Hamburger M., Mäser P. (2020). HPLC-Based Activity Profiling for Antiprotozoal Compounds in *Croton gratissimus* and *Cuscuta hyalina*. Front. Pharmacol..

[263] Guerra J.A., Molina M.F., Abad M.J., Villar A.M., Paulina B. (2006). Inhibition of inducible nitric oxide synthase and cyclooxygenase-2 expression by flavonoids isolated from *Tanacetum microphyllum*. Int. Immunopharmacol..

[264] Castillo Q.A., Triana J., Eiroa J.L., Padrón J.M., Plata G.B., Abel-Santos E.V., Báez L.A., Rodríguez D.C., Jiménez M.A., Pérez-Pujols M.F. (2015). Flavonoids from *eupatorium illitum* and their antiproliferative activities. Pharmacogn. J..

[265] Saepou S., Pohmakotr M., Reutrakul V., Yoosook C., Kasisit J., Napaswad C., Tuchinda P. (2010). Anti-HIV1 Diterpenoids from Leaves and Twigs of *Polyalthia sclerophylla*. Planta Med..

[266] Filho A.A.d.S., Resende D.O., Fukui M.J., Santos F.F., Pauletti P.M., Cunha W.R., Silva M.L.A., Gregório L.E., Bastos J.K., Nanayakkara N.P.D. (2009). In vitro antileishmanial, antiplasmodial and cytotoxic activities of phenolics and triterpenoids from *Baccharis dracunculifolia D. C*. (Asteraceae). Fitoterapia.

