# Supplementary material for: Stevia Genus: Phytochemistry and Biological Activities Update
Source: Molecules. 2021 May 6;26(9):2733. doi: 10.3390/molecules26092733 (PMC8125113; doi:10.3390/molecules26092733)
Supplement: Supplementary file 1 [file molecules-26-02733-s001.zip › molecules-1194022-supplementary.pdf]

Supplementary Materials

Stevia genus: phytochemistry and biological activities update

Jimena Borgo [1,2,3], Laura C. Laurella [1,2], Florencia Martini [1,3], Cesar A. N. Catalán [4], and Valeria P. Sülsen [1,2,3,*]

[1] CONICET—Universidad de Buenos Aires, Instituto de Química y Metabolismo del Fármaco (IQUIMEFA), Junín 956 2° piso, 1113 Buenos Aires, Argentina; jimeborgo@gmail.com (J.B); lclposdoc@gmail.com (L.C.L); vsulsen@ffyb.uba.ar (V.P.S)
[2] Cátedra de Farmacognosia, Facultad de Farmacia y Bioquímica, Universidad de Buenos Aires, Junín 956 2° piso, 1113 Buenos Aires, Argentina
[3] Cátedra de Química Medicinal, Facultad de Farmacia y Bioquímica, Universidad de Buenos Aires, Junín 956 1° piso, 1113 Buenos Aires, Argentina; flormartini1@gmail.com (F.M.)
[4] CONICET—Universidad Nacional de Tucumán, Instituto de Química del Noroeste—CONICET (INQUINOA), Ayacucho 471, San Miguel de Tucumán T4000INI, Argentina; ccatalan@fbqf.unt.edu.ar (C.A.N.C)

*Correspondence: vsulsen@ffyb.ub.ar (V.P.S)

Table S1. Compounds isolated from *Stevia* species since 1998

Species	Comp. N°	Type of compound	Common name	Structure	Reported activity	Ref.
Stevia connata	**1**	Longipinene	Longipinane-7β,8α,9α-triol-1-one 7-angelate-8-methylbutyrate	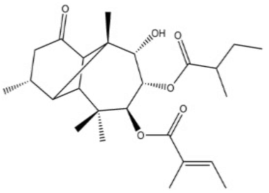	--	[18]
	**2**	Longipinane	Longipinane-7β,8α,9α-triol-1-one 8,9-diangelate	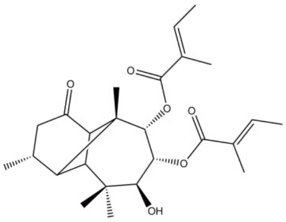	--	[18]
	**3**	Longipinene	Longipinane-7β,8α,9α-triol-1-one 7,9-diangelate	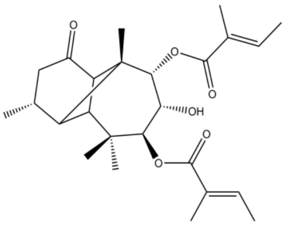	--	[18]
	**4**	Longipinene	Longipinane-7β,8α,9α-triol-1-one 7,8-diangelate	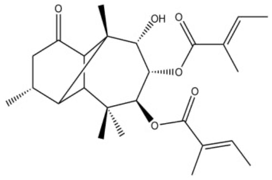	--	[18]
	**5**	Longipinene	Longipin-2-ene-7β,8α,9α-triol-1-one 7,8-diangelate	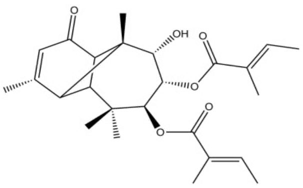	--	[18]
	**6**	Longipinene	Longipin-2-ene-7β,8α,9α-triol-1-one 8,9-diangelate	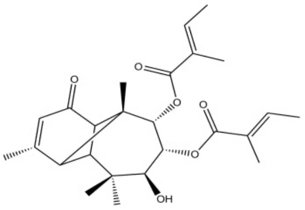	--	[18]
	**7**	Longipinene	Longipin-2-ene-7β,8α,9α-triol-1-one 8-angelate-9-methylbutyrate	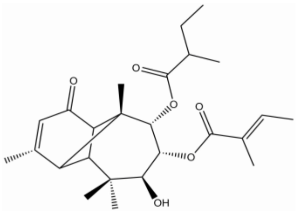	--	[18]

	**8**	Longipinene	Longipin-2-ene-7β,8α,9α-triol-1-one 7-angelate-8-methylbutyrate	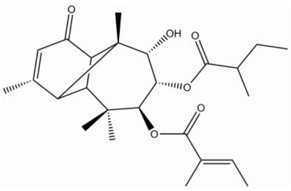	--	[18]
	**9**	sterol	Stigmasterol	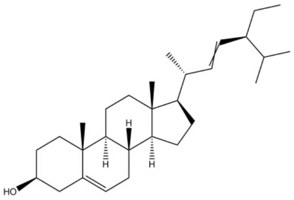	--	[18]
S. subpubescens	**10**	Diterpene	(4S,5S,6R,8S,9R,10S,13R)-9,13-Epoxy-14-labdene-6,19-diol	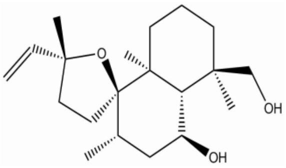	--	[19]
	**11**	Diterpene	(4S,5S,8S,9R,10S,13R)-9,13-Epoxy-14-labden-19-ol	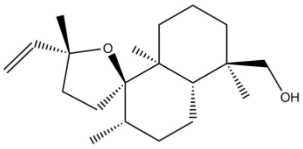	--	[19]
	**12**	Diterpene	(4S,5S,8S,9R,10S,13R)-9,13-Epoxy-14-labden-19-ol-6-one	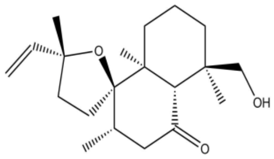	--	[19]
	**13**	Diterpene	(4S,5R,8S,9R,10S,13R)-9,13-Epoxy-14-labden-19-oic acid	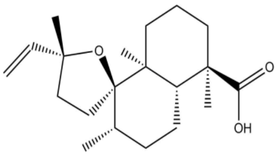	--	[19]
	**14**	Diterpene	3,4β-epoxy-5β,10β-cis-17α,20α-clerod-13(14)-en-15,16-olide	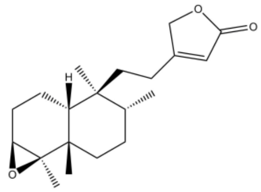	--	[19]
S. viscida	**15**	Triterpene	8,14-seco-oleana-8(26),13-dien-3β-ol	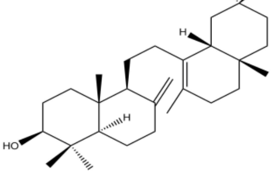	--	[20]

S. eupatoria	**16**	Triterpene	8,14-seco-oleana-8(26),13-dien-3β-acetate	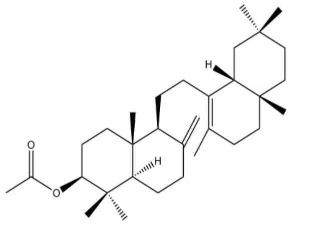	--	[20]
S. pilosa	**17**	Longipinene	(4R,5S,7S,8S,9S,10R,11R,200S)-7-angeloyloxy-9-hydroxy-8-(α-methylbutyryloxy)-longipin-2-en-l-one	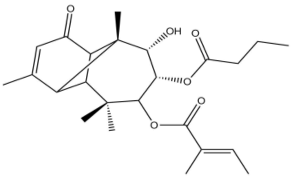	--	[21]
	**18**	Longipinene	(4R,5S,7S,8R,10R,11R,200S)-7-angeloyloxy-8-(a-methylbutyryloxy)- longipin-2-en-l-one	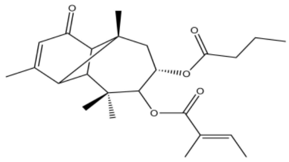	--	[21]
	**19**	Longipinene	--	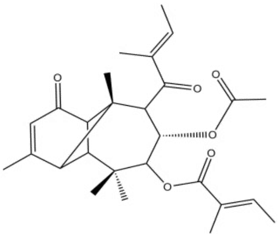	--	[21]
	**20**	Longipinene	--	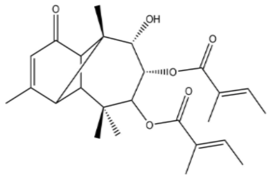	--	[21]
	**21**	Longipinene	--	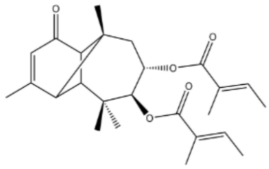	--	[21]
	**22**	Longipinene	--	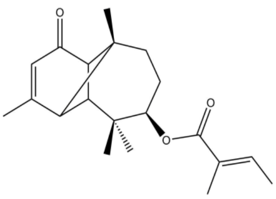	--	[21]

S. monardifolia	**23**	Longipinene	7β,8α-diangeloyloxylongipin-2-en-1-one	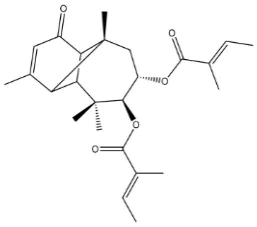	--	[22]
	**24**	Longipinene	7β,8α-diangeloyloxylongipinan-1-one	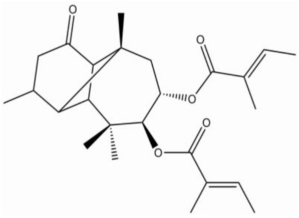	--	[22]
	**25**	Longipinene	7β-angeloyloxy-8α-isovaleroyloxylongipin-2-en-1-one	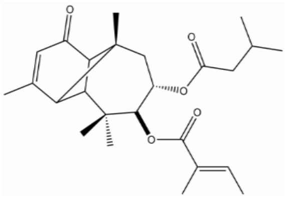	--	[22]
S. tomentosa	**26**	Sesquiterpene lactone	1,5:3,4-diepoxyguaia-10(14)-en-12,8-olide	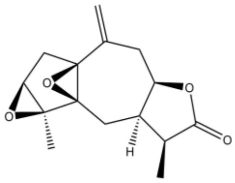	--	[23]
S. phlebophylla	**27**	Diterpene glycoside	16β-hydroxy-17-acetoxy-ent-kauran-19-oic acid(6-O-β-D-xylopyranosyl-β-D-glucopyranosyl) ester	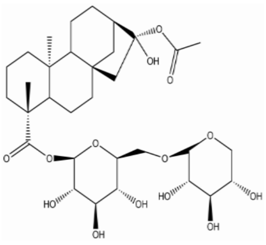	--	[24]
S. satureiifolia var. satureiifolia	**28**	Flavonoid	Eupatorin	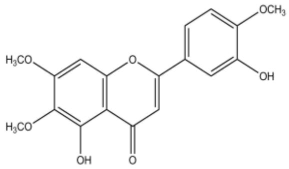	Tripanocidal. Leishmanicidal	[25]
	**29**	Flavonoid	Cirsimaritin	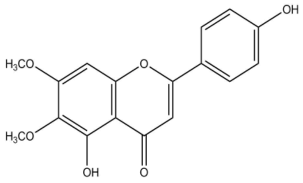	Tripanocidal. Leishmanicidal	[25]

	**30**	Flavonoid	5-desmethylsinensetin	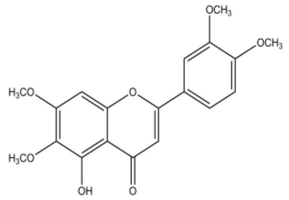	Tripanocidal. Leishmanicidal	[25]
S. serrata	**31**	Sesquiterpene	Chamazulene	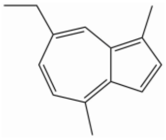	Antinociceptive	[26]
	**32**	Sesquiterpene	(E)-nerolidol	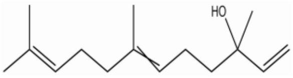	Antinociceptive	[26]
	**33**	Sesquiterpene	caryophyllene oxide	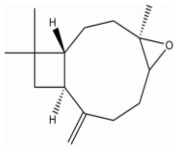	Antinociceptive	[26]
	**34**	Sesquiterpene	germacrene D	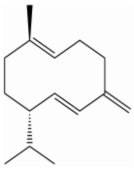	Antinociceptive	[26]
S. urticifolia	**35**	Flavonoid	Hispidulin	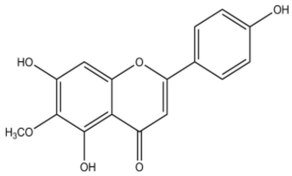	Antioxidant. Antiproliferative	[27]
	**36**	Flavonoid	Nepetin	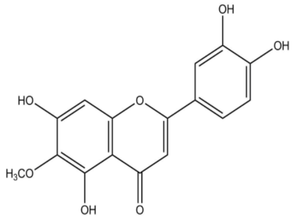	Antioxidant. Antiproliferative	[27]
	**37**	Flavonoid	Quercetin	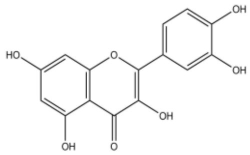	Antioxidant. Antiproliferative	[27]
	**38**	Flavonoid heteroside	Avicularin or Quercetin-3-O-α-L-arabinofuranoside	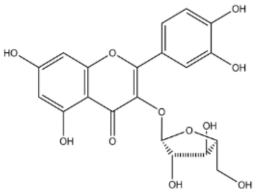	Antioxidant. Antiproliferative	[27]
S. subpubescens var. subpubescen	**39**	Flavonoid	4′-*O*-methylsakuranetin	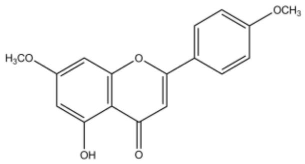	Antiinflammatory	[28]

s	**40**	Flavonoid	Sakuranetin	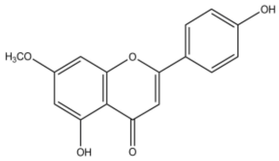	Antiinflammatory	[28]
	**41**	Flavonoid	3,7,4′-O-trimetilkaempterol	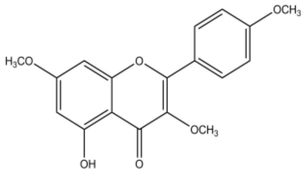	Antiinflammatory	[28]
	**42**	Flavonoid	Ayanin	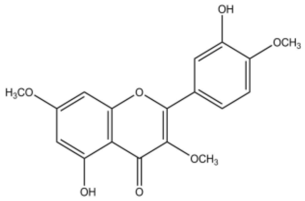	Antiinflammatory	[28]
	**43**	Flavonoid	Ermanin	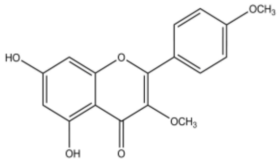	Antiinflammatory	[28]
	**44**	Flavonoid heteroside	Hyperin	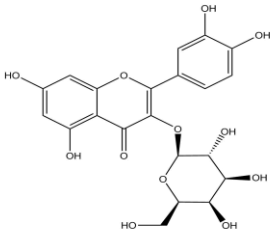	Antiinflammatory	[28]
	**45**	Labdane	Cistenolic acid	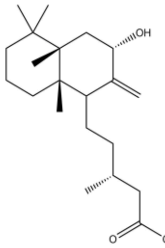	Antiinflammatory	[28]
	**46**	Labdane	Labdanolic acid	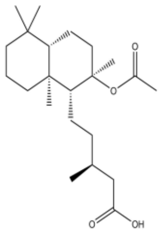	Antiinflammatory	[28]
	**47**	Coumarin	Scoparone	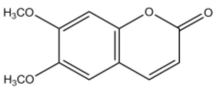	Antiinflammatory	[28]
	**48**	Glucose ester of o-coumaric acid	Melilotoside	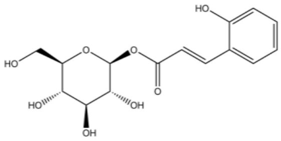	--	[28]

	**49**	glycoside	L-chiro inositol	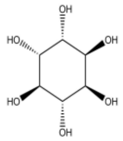	--	[28]
S. jorullensis	**50**	Sesquiterpene lactone	11β,13-dihydrocostunolide	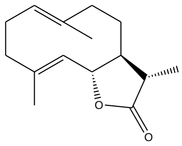	--	[29]
	**51**	Sesquiterpene lactone	11β,13-dihydroreynosin	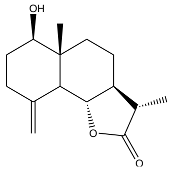	--	[29]
	**52**	Sesquiterpene lactone	1β-dihydroxycolartin	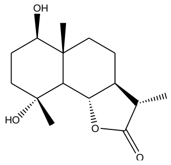	--	[29]
	**53**	phenolic	Chlorogenic acid	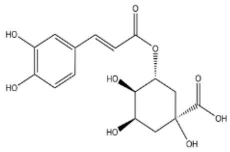	--	[29]
	**54**	sterol	β-sitosterol	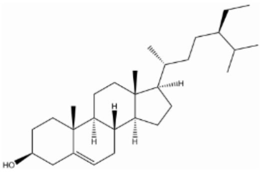	--	[29]
	**55**	Steryl glycoside	β-sitosterylglucopyranoside	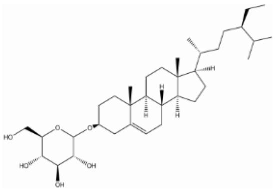	--	[29]
	**56**	Steryl glycoside	Stigmasterylglucopyranoside	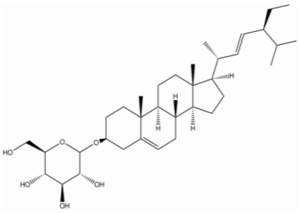	--	[29]
S. lucida	**57**	Sesquiterpene lactone	Alantolactone	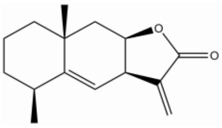	Anti-*Trypanosoma cruzi.* Anti-*T. brucei.* Antineoplastic. Antimicrobial.	[30, 86, 87, 90]

**58**	Sesquiterpene lactone	Isoalantolactone	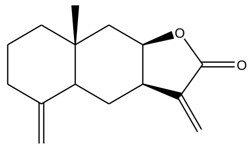	Anti-*Trypanosoma cruzi.* Anti-*T. brucei.* Antineoplastic. Antimicrobial.	[30, 86, 87, 90]
